# Deciphering the role of recurrent FAD-dependent enzymes in bacterial phosphonate catabolism

**DOI:** 10.1016/j.isci.2023.108108

**Published:** 2023-10-04

**Authors:** Erika Zangelmi, Francesca Ruffolo, Tamara Dinhof, Marco Gerdol, Marco Malatesta, Jason P. Chin, Claudio Rivetti, Andrea Secchi, Katharina Pallitsch, Alessio Peracchi

**Affiliations:** 1 Department of Chemistry, Life Sciences and Environmental Sustainability, University of Parma, 43124 Parma, Italy; 2 Institute of Organic Chemistry, Faculty of Chemistry, University of Vienna, 1090 Vienna, Austria; 3 Vienna Doctoral School in Chemistry (DoSChem), University of Vienna, 1090 Vienna, Austria; 4 Department of Life Sciences, University of Trieste, Via Giorgieri 5, 34127 Trieste, Italy; 5 School of Biological Sciences and Institute for Global Food Security, Queen’s University Belfast, 19 Chlorine Gardens, BT9 5DL Belfast, UK

**Keywords:** Enzymology, Bacteriology, Microbial metabolism

## Abstract

Phosphonates—compounds containing a direct C–P bond—represent an important source of phosphorus in some environments. The most common natural phosphonate is 2-aminoethylphosphonate (AEP). Many bacteria can break AEP down through specialized “hydrolytic” pathways, which start with the conversion of AEP into phosphonoacetaldehyde (PAA), catalyzed by the transaminase PhnW. However, the substrate scope of these pathways is very narrow, as PhnW cannot process other common AEP-related phosphonates, notably *N*-methyl AEP (M_1_AEP). Here, we describe a heterogeneous group of FAD-dependent oxidoreductases that efficiently oxidize M_1_AEP to directly generate PAA, thus expanding the versatility and usefulness of the hydrolytic AEP degradation pathways. Furthermore, some of these enzymes can also efficiently oxidize plain AEP. By doing so, they surrogate the role of PhnW in organisms that do not possess the transaminase and create novel versions of the AEP degradation pathways in which PAA is generated solely by oxidative deamination.

## Highlights

• 2-aminoethylphosphonate (AEP) is the most common natural phosphonate
• FAD oxidoreductases are often encoded in bacterial gene clusters for AEP catabolism
• These enzymes serve to degrade the naturally occurring *N*-monomethyl-AEP
• Some of these enzymes also act on AEP, creating a novel AEP catabolic pathway

## Graphical abstract

**Figure:**
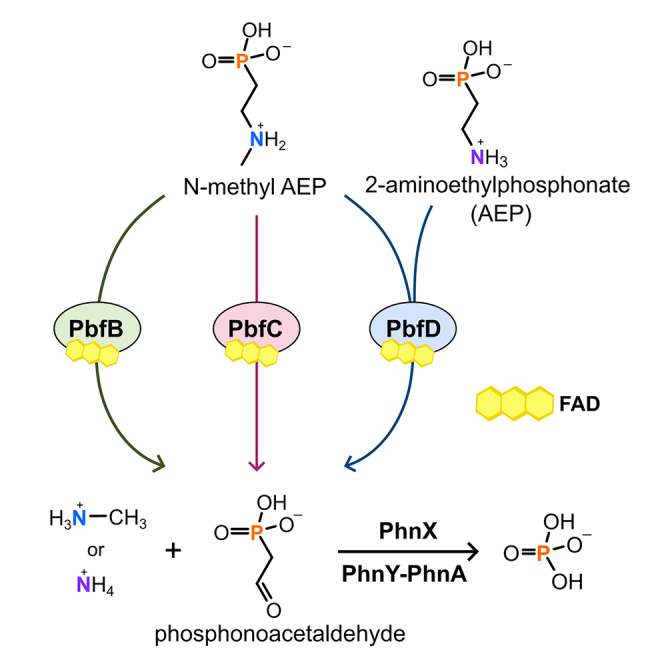

## Introduction

For an organism, the ability to consume multiple distinct but related compounds through a single catabolic pathway represents a straightforward and economical way to exploit different nutrient sources and hence increase organismal fitness under variable nutrient conditions. This is exemplified by the archetypal catabolic pathway, glycolysis, which is commonly described as a route for the degradation of glucose, yet it also catabolizes several other sugars which are funneled into glycolysis by various ancillary enzymes or short “tributary” pathways.^1^^,^^2^^,^^3^ Examples of this kind are widespread also in other, less central catabolic routes. For instance, in *Escherichia coli* a single enzyme has been described that transforms two different dehydrated forms of *N*-acetylneuraminate (sialic acid) into plain sialic acid, to feed the dedicated degradation pathway.^4^

**Figure 1:**
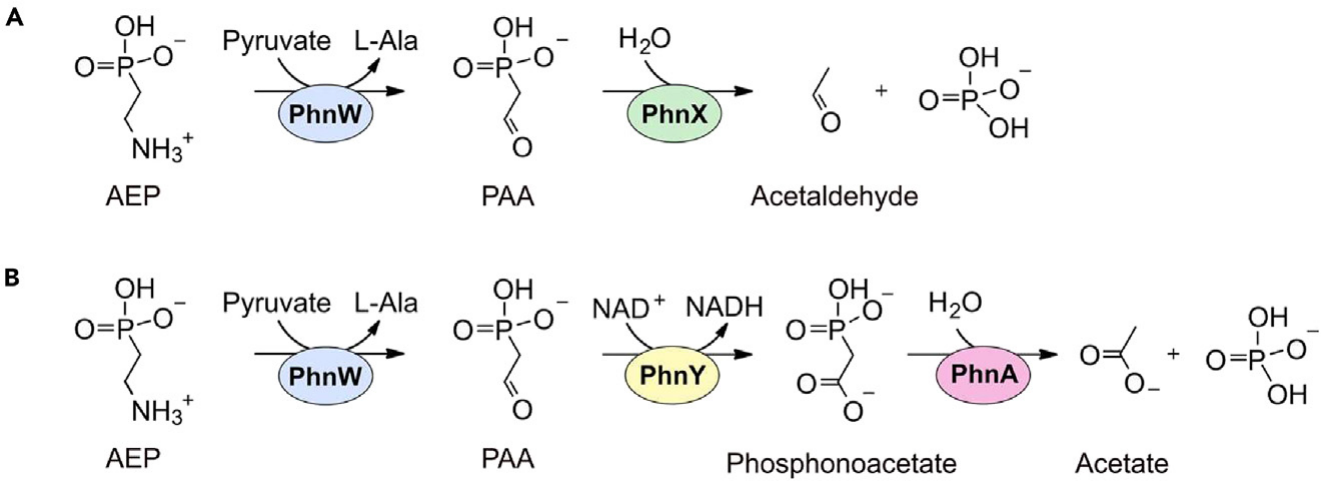
Hydrolytic pathways for the microbial catabolism of AEP (A) In the simplest version of this pathway, the transaminase PhnW converts AEP to PAA, which in turn is cleaved into acetaldehyde and phosphate by PAA hydrolase (PhnX). (B) Variant of the pathway in A where PAA is first converted to phosphonoacetate and then to acetate and phosphate by the enzymes PhnY and PhnA, respectively. It should be noted that AEP can also be degraded through an “oxidative” pathway (totally unrelated to the pathways depicted here), which proceeds through the consecutive reactions of two dioxygenases (PhnY∗ and PhnZ) and yields as final products glycine and phosphate.^17^^,^^18^^,^^19^

Herein, we looked for ancillary enzymes that might expand the versatility of the catabolic pathways for 2-aminoethylphosphonate (AEP; also known as ciliatine), which is the most prevalent natural phosphonate.^5^^,^^6^ Phosphonates are molecules containing a direct C–P bond in place of the more common C–O–P ester linkage^7^; they occur in the environment not only as anthropogenic pollutants^8^ but also because they are produced by a variety of organisms, including mollusks, protozoa, and bacteria.^9^^,^^10^^,^^11^^,^^12^ Many environmental microorganisms are able to degrade phosphonates, especially to retrieve phosphorus in habitats where this element is limiting for growth.^13^^,^^14^^,^^15^ In particular, since AEP is abundant in nature,^5^^,^^6^^,^^14^^,^^16^ a number of bacteria possess specialized “hydrolytic” pathways for the degradation of this compound (Figure 1).

In the most common case, the hydrolytic degradation pathway begins with a reaction catalyzed by the transaminase PhnW, which converts AEP into phosphonoacetaldehyde (PAA)^20^; subsequently, PAA is cleaved into acetaldehyde and inorganic phosphate by the hydrolase PhnX (Figure 1A). In an alternative pathway, PAA is converted to phosphonoacetate by the dehydrogenase PhnY and finally to acetate and phosphate by the hydrolase PhnA^21^^,^^22^ (Figure 1B).

Given the widespread diffusion of both the PhnWX and PhnWYA pathways, it is biologically plausible that accessory enzymes may have evolved to convey into these catabolic routes other naturally occurring, AEP-related phosphonates. As a case in point, we recently reported the identification and characterization of one enzyme that is encoded in over 13% of the bacterial gene clusters containing the *phnWX* (or *phnWYA*) combination.^23^ We termed this enzyme PbfA (for “phosphonate breakdown factor A″) and eventually showed that it is a lyase acting on the natural compound *(R)*-2-amino-1-hydroxyethylphosphonate (*R-*HAEP). The reaction catalyzed by PbfA generates PAA, which can be subsequently processed by PhnX (or by PhnY).^23^

Encouraged by the discovery of PbfA, we performed further genomic analyses, parsing firstly the bacterial gene clusters of the *phnWX* type in search of additional enzyme-coding genes recurring within these clusters. As in the case of PbfA, we relied on the assumption that enzymes able to “funnel” different compounds into the AEP hydrolytic pathway would be genomically associated with *phnW* and *phnX.*

During this analysis, we were struck by the frequent presence, in these clusters, of genes apparently coding for oxidoreductases dependent on flavin adenine dinucleotide (FAD). In terms of sequence, these putative enzymes were rather heterogeneous and fell into at least three distinct subgroups, which we termed PbfB, PbfC, and PbfD. However, irrespective of their subgroup, all these proteins showed a substantial similarity to amine oxidoreductases, i.e., enzymes that oxidize various primary and secondary amines, leading to the formation of aldehydes or ketones.^24^^,^^25^^,^^26^ By analogy, we hypothesized that the identified FAD-dependent enzymes could oxidize some N-alkylated form of AEP, or perhaps of (*R*)-HAEP; in particular, they could act on *N*-methyl AEP (M_1_AEP), which is another frequently found natural product^27^^,^^28^^,^^29^^,^^30^^,^^31^^,^^32^^,^^33^ but which cannot be processed by PhnW.

Indeed, we present evidence that enzymes from all three groups are able to convert M_1_AEP to PAA, which can be subsequently processed to generate inorganic phosphate. We characterized in detail representative enzymes from the PbfC and PbfD groups, highlighting substantial mechanistic differences between them. In particular, a PbfD enzyme from *Mariniblastus fucicola* was found to oxidize plain AEP and M_1_AEP with comparable efficiencies, suggesting that *in vivo* this enzyme can perform a double duty. By generating PAA not only from M_1_AEP but also from AEP, this oxidoreductase would in effect surrogate the function of the transaminase PhnW, whose gene is absent in the *M. fucicola* gene cluster.

## Results

### Genes for FAD-dependent oxidoreductases are often present in clusters dedicated to AEP degradation

We visually inspected the genomic contexts of the genes implicated in the hydrolytic AEP degradation routes, beginning with the pyridoxal phosphate (PLP)-dependent aminotransferase PhnW, which converts AEP to PAA (Figure 1). Because it is known that PhnW can play a role in both the biosynthesis and degradation of AEP,^21^ we focused on PhnW homologs (>35% identical to the validated enzymes from *Salmonella enterica*^20^ or *Vibrio splendidus*^23^) whose genes clustered with *phnX* or with the *phnY/phnA* duo (*phnWX* and *phnWYA* clusters). Conversely, we excluded from the analysis those cases in which *phnW* was located close to the *pepM* and *ppd* genes, which are known to participate in AEP biosynthesis.^11^^,^^12^^,^^34^

By examining the AEP degradative clusters in many organisms, we noticed that such clusters often included genes apparently coding for FAD-dependent oxidoreductases (Pfam: PF01266). These genes were found associated with clusters of both the *phnWX* and the *phnWYA* type. Furthermore, upon expanding our analysis, we observed that genes for similar FAD-dependent enzymes were sometimes also associated with *phnX* alone (i.e., they occurred in clusters lacking *phnW*). Exemplary cases of different cluster types that include these enzymes are shown in Figure S1.

**Figure 2:**
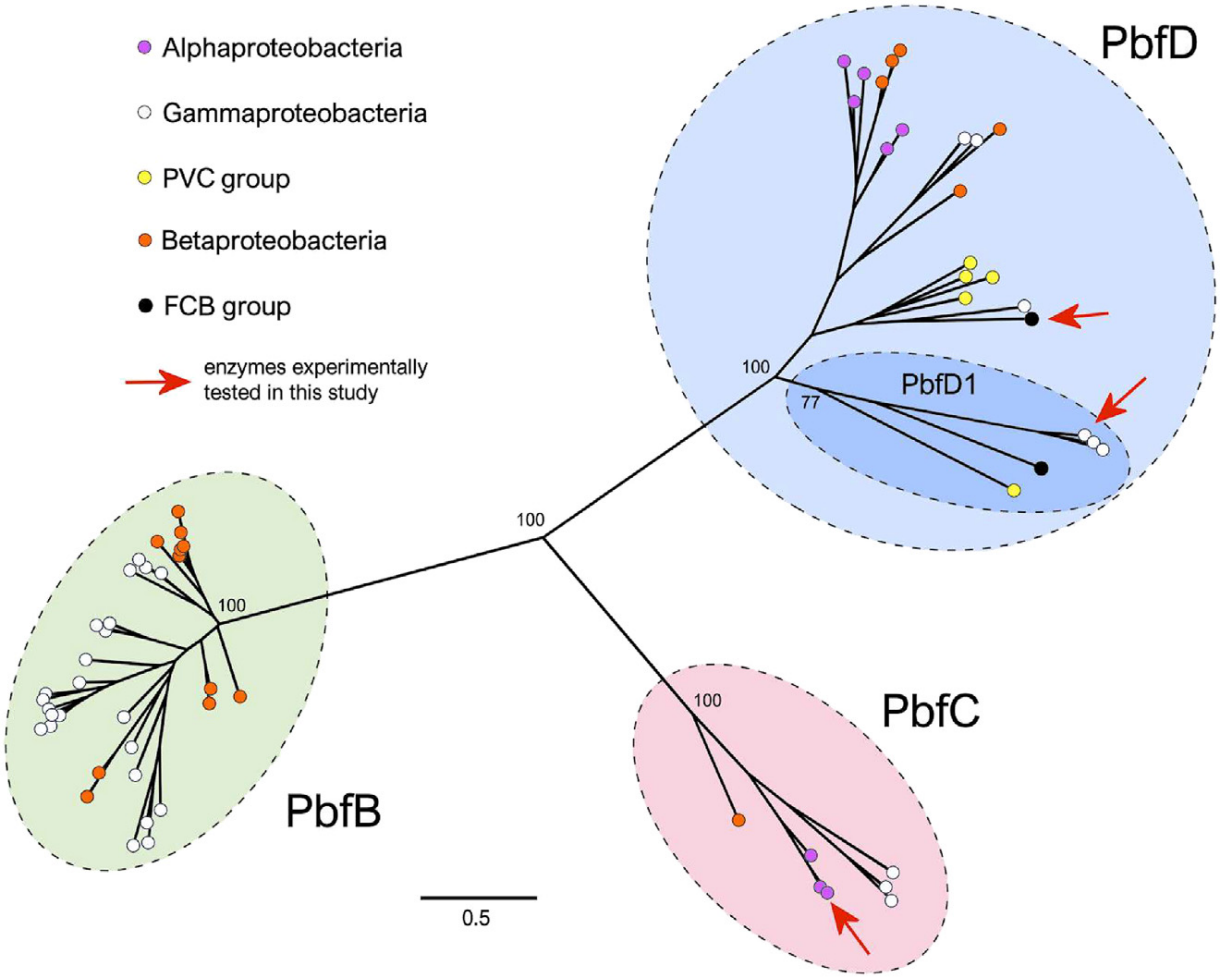
Simplified maximum likelihood unrooted phylogenetic tree of 64 representative FAD-dependent enzymes encoded in gene clusters for the degradation of AEP The tree only reports bootstrap values for the main basal nodes and those supported by bootstrap values <50 were collapsed. Full bootstrap metrics, together with organisms’ names, are reported in Figure S3, whereas the sequence accession IDs are provided in Table S1. The scale bar indicates the number of substitutions per site. Groups of sequences that we labeled PbfB, PbfC, and PbfD are highlighted in light green, light red and light blue, respectively. The monophyly of all three clades was extremely well supported (bootstrap values = 100), in line with the high sequence divergence observed between groups (i.e., median inter-group p-distances was equal to = 0.81). A subgroup of monophyletic early branching PbfD sequences, termed PbfD1, was also well-supported (bootstrap value = 77) and is signaled by a darker shade of blue. *pbfD1* genes usually clustered with *phnW* and *phnX*, whereas other *pbfD* genes were most commonly associated with *phnX* alone (see Table S1). Related to Figures S1–S3 and Table S1.

Despite sharing the same conserved domain, the encoded protein sequences displayed a remarkable diversity, often retaining a very limited similarity within the FAD-dependent oxidoreductase domain (Figure S2). Phylogenetic inference strongly supported their clustering in three distinct subgroups, which we—in keeping with the nomenclature adopted for PbfA^23^—termed PbfB, PbfC, and PbfD (Figures 2 and S3).

While these three subgroups of FAD-dependent proteins were sequence-wise quite dissimilar, they were consistently related to enzymes that catalyze the oxidative deamination of primary or secondary amines. This type of reaction leads to the formation of intermediate imine species, which spontaneously hydrolyze with the eventual generation of carbonyl groups.^24^^,^^25^^,^^26^ The functionally validated enzymes most similar to PbfB, PbfC, and PbfD are reported in Table 1 and their reactions are shown in Figure S4. The majority of these enzymes are classified as oxidases, indicating that they use molecular oxygen as the physiological electron acceptor and hence produce H_2_O_2_. However, for the mammalian sarcosine dehydrogenase (SarDH) the physiological electron acceptor is a flavoprotein^42^; whereas for the D-amino acid dehydrogenase from *Helicobacter pylori* (DadA) the electron acceptor is presumably a quinone^40^ (Figure S4).

**Table 1:** Percent identities between representative PbfB, PbfC, and PbfD enzymes and the most similar enzymes of known function

	PuuB	MabO	ThiO	SolA	SarDH	DadA	SoxA
PbfB *V. vulnificus*	**31.2%**	28.6%	25.7%	23.1%	24.7%	22.4%	22.8%
PbfC *Azospirillum* sp. *B510*	25.8%	**30.2%**	27.6%	24.5%	26.5%	22.2%	27.7%
PbfD1 *A. baumannii*	23.4%	24.5%	**25.2%**	23.9%	20.6%	22.9%	25.0%
PbfD2 *M. fucicola*	25.7%	23.1%	24.9%	**27.2%**	24.9%	23.8%	22.5%

The enzymes of known function are: PuuB, γ-glutamyl putrescine oxidase from *E. coli*^35^; MabO, 4-methylamino butyrate oxidase from *Arthrobacter nicotinovorans*^36^; ThiO, glycine oxidase from *Bacillus licheniformis*^37^; SolA, N-methyl-L-tryptophan oxidase from *E. coli*^38^; SarDH, sarcosine dehydrogenase from *Rattus norvegicus*^39^; DadA, D-amino acid dehydrogenase from *Helicobacter pylori*^40^; SoxA, sarcosine oxidase from *Bacillus* sp. *B-0618*.^41^ The GenBank accession IDs for these enzymes are found in Table S2 and the reactions they catalyze are shown in Figure S4. Percent identities were evaluated by the pairwise global alignment of the portions of the sequences denoting the PF01266 domain, carried out with Needle (https://www.ebi.ac.uk/Tools/psa/emboss_needle/) with a Blosum30 substitution matrix. Percent identities rarely exceed 30%, indicative of only distant homologies between the oxidoreductases described in this study and the other functionally characterized PF01266 enzymes. A more refined analysis of the evolutionary relationships between the enzymes in this table is provided in Figure S5. Related to Table S2, Figures S4 and S5.

### Hypotheses on the function of the FAD-dependent oxidoreductases

Enzymes from the PbfB, PbfC, and PbfD subgroups were never co-present in the same cluster. This observation, together with the fact that the different FAD-dependent enzymes were invariably similar to amine oxidoreductases, suggested that they all might be performing essentially the same transformation in aminophosphonate degradation.

But what could be the function of these oxidoreductases? As outlined in the introduction, we assumed that these enzymes served to degrade compounds related to AEP. More detailed hypotheses about their actual activity could be formulated based on the following considerations: (i) the substrate of these enzymes should contain a primary or secondary amino group, consistent with the reactions catalyzed by the most closely related enzymes of known function (Figure S4); (ii) the product of the reaction should presumably be either AEP or PAA, in order to enter the PhnWX or PhnWYA pathway.

**Scheme 1:**
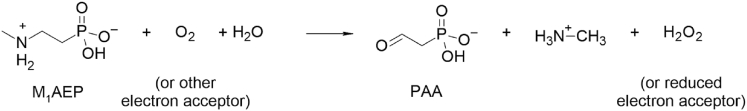
A proposal for the reaction catalyzed by the FAD-dependent enzymes described in this study

Based on these considerations, our initial hypothesis was that PbfB, PbfC, and PbfD enzymes could all catalyze the oxidative deamination of some AEP derivative(s) alkylated on the amino group. In particular, they could act on the *N*-methyl derivative of AEP (M_1_AEP), which is reportedly quite common in nature.^27^^,^^28^^,^^29^^,^^30^^,^^31^^,^^32^^,^^33^ This compound cannot be processed by PhnW, which—like all PLP-dependent transaminases—requires a substrate with a primary amino group. We thus envisaged that the FAD-dependent oxidoreductases might carry out the reaction shown in Scheme 1.

The direct oxidation of M_1_AEP would produce methylamine and PAA, which could be then conveniently hydrolyzed by PhnX. Given the structural similarity of M_1_AEP to plain AEP, we also expected that the FAD enzymes could oxidize AEP with some efficiency, producing PAA with the release of ammonia. This reaction would be redundant with respect to the reaction catalyzed by PhnW, which also generates PAA from AEP (Figure 1); however, in those organisms that do not possess PhnW (e.g., *M. fucicola*—Figure S1), the oxidative deamination of AEP could be not just biologically acceptable, but wholly desirable.

### The PbfC and PbfD enzymes oxidize M_1_AEP

Synthetic genes encoding the PbfB protein from *Vibrio vulnificus*, the PbfC protein from *Azospirillum* sp. B510, and the PbfD proteins from *Acinetobacter baumannii* (henceforth termed PbfD1) and *Mariniblastus fucicola* (henceforth termed PbfD2) were purchased with codon optimization for expression in *E. coli*. The genes were inserted in the bacterial expression vector pET28a and the recombinant His-tagged proteins were expressed and purified from *E. coli* cells as described in the STAR Methods.

All four recombinant proteins appeared to be soluble to some extent; however, despite our efforts, PbfB could not be purified by metal-affinity chromatography from cell extracts, which greatly limited its characterization (see in the following section). On the other hand, we obtained the recombinant PbfC, PbfD1, and PbfD2 enzymes in pure form and proceeded to characterize their catalytic activity toward a number of potential substrates, including some non-phosphonate AEP analogs.

We initially used 96-well plate assays to test the actual ability of the FAD enzymes to oxidize various amino compounds, employing the artificial dye DCPIP as the final electron acceptor and PMS as an electron mediator.^43^ DCPIP is routinely used in the field of FAD-dependent enzymes to measure the activity of both oxidases^44^^,^^45^^,^^46^ and dehydrogenases.^43^^,^^46^^,^^47^ Using this assay, any oxidation reaction of the substrate results in DCPIP turning from blue to colorless, so that the color of the reaction mixture shifts from green to yellow (the yellow color being due to the presence of PMS).

A second type of colorimetric assay served to evaluate the ability of the enzymes to oxidize potential substrates with electron transfer to oxygen. The assay measured the formation of H_2_O_2_, which can be used by horseradish peroxidase to oxidize the chromogenic compound *o*-dianisidine, turning the solution to deep pink. Contrary to the DCPIP assay, this method entailed a single readout taken after a relatively long incubation, thus it could only qualitatively assess the capacity of the enzyme to reduce molecular oxygen.

**Figure 3:**
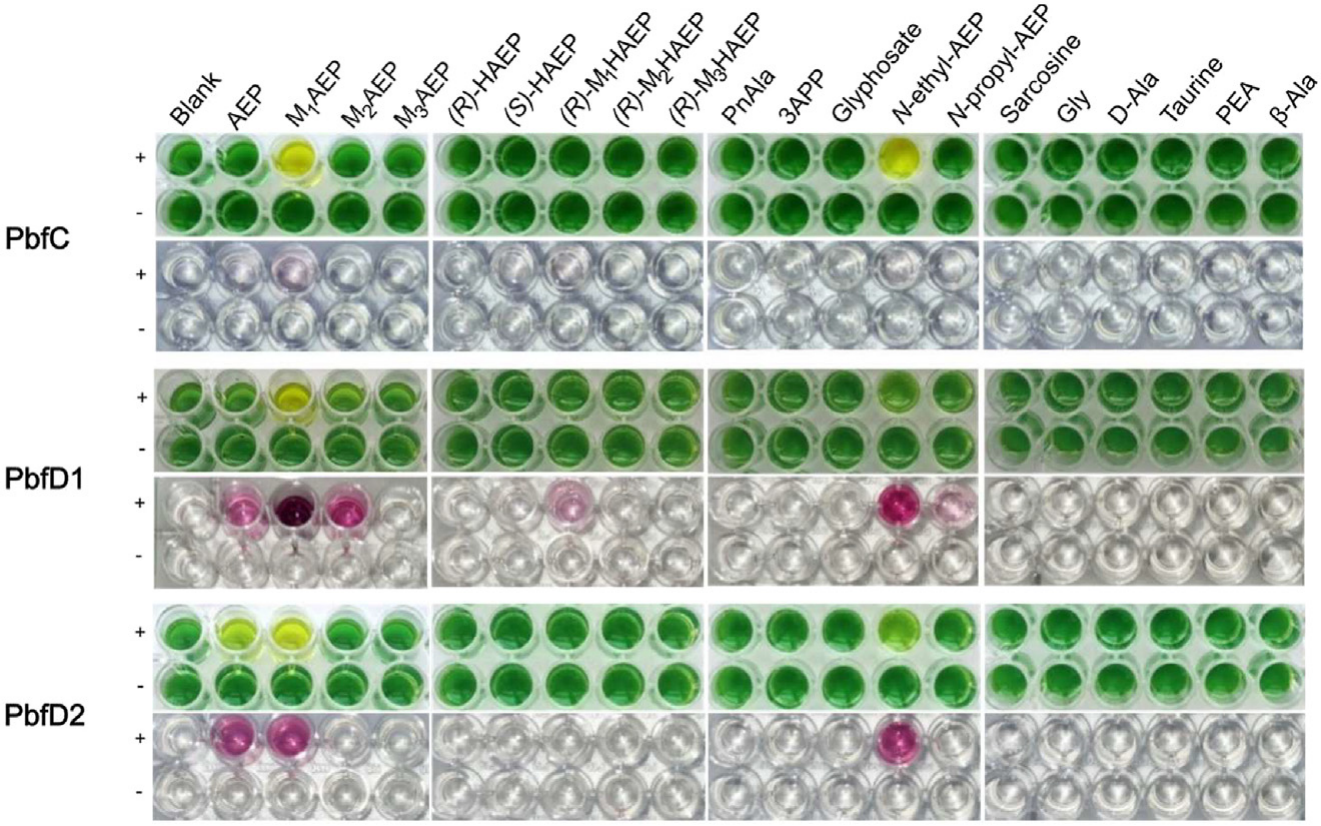
Microtiter assays to test the activity of PbfC, PbfD1, and PbfD2 toward a panel of potential (amino-containing) substrates In the green/yellow wells, the potential substrates were incubated at room temperature in the presence (+) or absence (−) of the oxidoreductases (0.5 μM); the mixture also contained the redox dye DCPIP (which served as the final electron acceptor) and the redox mediator PMS. Reactions were started by adding the enzyme last and photographed at regular intervals—the images in this figure were taken 6 min after adding the enzyme. In wells where the reaction occurred, the solution turned from green to yellow. The transparent/pink wells refer instead to an endpoint microtiter assay aimed at detecting the release of hydrogen peroxide. The reactions were conducted as aforementioned, except that artificial dyes were omitted and molecular oxygen served as the electron acceptor. After 30 min, the wells were supplemented with horseradish peroxidase and *o*-dianisidine and (after another 5 min) sulfuric acid. Wells where hydrogen peroxide had formed turned deep pink. Abbreviations: PnAla, 3-phosphono-DL-alanine; 3APP, 3-aminopropylphosphonate; PEA, phosphoethanolamine. Related to Figures S6 and S7.

The results of these microtiter assays are reported in Figure 3. Consistent with our initial hypothesis, these preliminary assays implied a strong reactivity of all three oxidoreductases with M_1_AEP, as well as some activity toward the closely related compound *N*-ethyl-AEP (which, however, is not known to occur in nature). In particular, PbfC did not react appreciably with other compounds: moreover, activity of this enzyme was readily observed in the DCPIP assay, but barely detectable in the peroxidase-based assay, suggesting that PbfC inefficiently uses molecular oxygen as the electron acceptor.

On the other hand, PbfD1 and PbfD2 enzymes manifested a strong oxidative activity toward M_1_AEP in both the DCPIP-based assay and the peroxidase-*o*-dianisidine assay. These two enzymes also showed a clear reactivity toward AEP. Furthermore, PbfD1, but not PbfD2, appeared to appreciably oxidize *N,N*-dimethyl-2-aminoethylphosphonate (M_2_AEP).

None of the enzymes reacted with non-phosphonate analogs of M_1_AEP (such as sarcosine) or of AEP (e.g., taurine, β-alanine). The enzymes also failed to oxidize 2-hydroxyethyl phosphonate, an analog of AEP that contains a hydroxyl group in place of the amino group (data not shown).

### The PbfC and PbfD enzymes generate PAA

Although the microtiter assays indicated the ability of the FAD enzymes to oxidize M_1_AEP, other details of the hypothesized reaction had to be confirmed.

In principle, the oxidation of M_1_AEP (but not AEP) could generate AEP and formaldehyde, instead of PAA and methylamine. To test for this possibility, we employed a spectrophotometric assay in which the three oxidoreductases were incubated with M_1_AEP for 30 min; afterward, NADH and alcohol dehydrogenase (ADH) were added to detect the oxidation of NADH in case formaldehyde had been produced. The observation that NADH was not appreciably consumed (data not shown) implied that formaldehyde had not been formed (PAA is not a substrate for ADH). Conversely, when the oxidoreductases were incubated for 30 min with M_1_AEP and PhnX, which hydrolyzes PAA to acetaldehyde, and then the reaction mixture was supplemented with ADH and NADH, the latter was quickly oxidized. This observation was fully consistent with the hypothesis that the PbfC and PbfD enzymes produce PAA.

We also demonstrated that the reaction of PbfD1 and PbfD2 with AEP (but not M_1_AEP) released ammonia, as revealed by a coupled assay with GDH (data not shown). Other controls established that phosphate was not released in the reaction of the oxidoreductases with either M_1_AEP or AEP, except when PhnX was also present (BIOMOL Green assay, data not shown).

Finally, the reaction of PbfC, PbfD1, or PbfD2 with M_1_AEP was monitored by ^1^H-NMR, establishing beyond doubt that the reaction products are methylamine and PAA. The results pertinent to the reactions of the three enzymes are summarized in Figure S6. NMR experiments also showed that the reaction of PbfD1 with M_2_AEP generates dimethylamine and PAA (Figure S7).

### The PbfC and PbfD enzymes have different propensities to reduce oxygen

**Figure 4:**
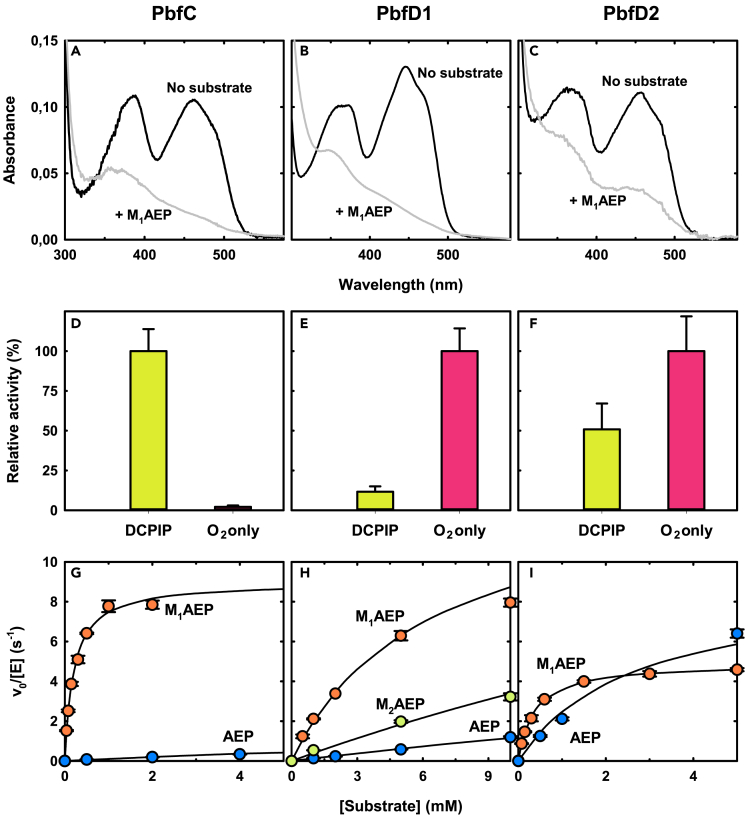
Spectroscopic and functional properties of PbfC, PbfD1, and PbfD2 (A–C) UV-vis absorption spectra of the three enzymes (10 μM) before (black line) and after (gray line) the addition of 20 mM M_1_AEP. Spectra were collected at room temperature under aerobic conditions in 50 mM pyrophosphate buffer pH 8.0 (PbfC) or 50 mM phosphate buffer pH 8.0 (PbfD1 and PbfD2). (D–F) Relative activities of the three enzymes in the presence of 5 mM M_1_AEP, measured at room temperature through the assay based on DCPIP-PMS (lime yellow bar) or through the coupled assay with PhnX and ADH (magenta bar). The error bars represent standard errors for triplicate experiments. Activity in the assay that gave the maximum rate was taken as 100%. Both types of assays were conducted in TEA-HCl buffer, pH 8.0. (G–I) Activity of the three enzymes toward naturally occurring substrates that had shown some reactivity in the microtiter assays (TEA-HCl buffer pH 8.0, 25°C). The activity of PbfC, was measured with the DCPIP reduction assay whereas the activities of PbfD1 and PbfD2 were measured under atmospheric oxygen through the PhnX-ADH coupled assay. Each data point represents a triplicate experiments; bars signal standard errors. Solid lines represent fittings of the data to the Michaelis-Menten equation. The fitting-derived kinetic parameters are reported in Table 2.

The purified PbfC, PbfD1, and PbfD2 enzymes appeared yellow, with their absorption spectra exhibiting two peaks in the visible range, typical of an oxidized flavin cofactor (Figures 4A–4C). The first peak was centered at ∼365 nm for both PbfD1 and PbfD2, but at 387 nm for PbfC. The second peak ranged from ∼447 nm for PbfD1 to ∼462 nm for PbfC (Figures 4A–4C). The cofactor was released in solution when the enzymes were heat-precipitated (10 min at 100°C, in the dark), suggesting that the flavin was not covalently bound. Furthermore, when the proteins were denatured using SDS, the spectrum of the released cofactor was typical of FAD^48^ (and not FMN; see STAR Methods). Addition of an excess of M_1_AEP to the native enzymes caused spectroscopic changes indicative of FAD reduction in all cases (Figures 4A–4C).

To assess the ability of the enzymes to transfer electrons from FAD to different acceptors, we used two continuous spectrophotometric assays. A first assay was based on the reduction of the artificial acceptor dye DCPIP (in the presence of the electron mediator PMS), as described earlier. The second assay was conducted in the absence of artificial electron acceptors and coupled the production of PAA with the consecutive reactions of PhnX (which converts PAA into acetaldehyde) and of alcohol dehydrogenases (that reduces acetaldehyde while consuming NADH).^23^ In this assay, molecular oxygen was assumed to serve as the electron acceptor for the oxidoreductases’ reaction.

Consistent with the findings of the microtiter assays in Figure 3, PbfC showed a very weak activity in the presence of oxygen alone, but was much more active when DCPIP could serve as the electron acceptor. Conversely, PbfD1 and PbfD2 were more active in the coupled assay, suggesting a strong propensity of these enzymes to directly reduce oxygen (Figures 4D–4F).

### Catalytic efficiency and specificity of the PbfC and PbfD enzymes

To establish the catalytic performances of the PbfC and PbfD oxidoreductases toward naturally occurring substrates, we measured the activity of these enzymes using the continuous spectrophotometric assays that worked best for each of them (i.e., the DCPIP reduction assay for PbfC and the coupled PhnX-ADH assay for the PbfD enzymes).The pertinent titrations fitted well to the Michaelis-Menten equation (Figures 4G–4I) and the apparent catalytic parameters are reported in Table 2.

**Table 2:** Apparent catalytic parameters of the FAD-dependent enzymes PbfC, PbfD1, and PbfD2

Enzyme	Substrate	k_cat_/K_M_ (M^−1^ s^−1^)	k_cat_ (s^−1^)	K_M_ (mM)
PbfC	M_1_AEP	45000 ± 2700	9.0 ± 0.2	0.20 ± 0.01
	AEP	110 ± 6	2.2 ± 0.1	20 ± 1.7
PbfD1	M_1_AEP	2300 ± 160	13.2 ± 0.54	5.8 ± 0.62
	AEP	130 ± 6	9.1 ± 1.7	71 ± 16
	M_2_AEP	400 ± 26	22 ± 4.5	54 ± 14
PbfD2	M_1_AEP	13500 ± 550	4.9 ± 0.07	0.36 ± 0.02
	AEP	3400 ± 320	8.9 ± 0.3	2.6 ± 0.32

The kinetic parameters were obtained by fitting the titration data reported in Figure 4 (panels G-I) to the Michaelis-Menten equation. The reported parameters (± SE of the fitting) are rounded to the first two significant figures; they are deemed ‘apparent’ because they were measured in the presence of a fixed concentration of the electron acceptor co-substrate. The activity of PbfC was assessed in the presence of 80 μM DCPIP and 3 mM PMS. Activities of the PbfD enzymes were determined in aerated solutions, i.e., in the presence of ∼0.25 mM O_2_. Related to Figure S8.

The apparent catalytic efficiencies (k_cat_/K_M_ values) of the three enzymes toward M_1_AEP were consistently in the range 2×10^3^ - 5×10^4^ M^−1^s^−1^, whereas K_M_ values ranged from about 0.2 mM to about 5 mM. These performances, while not outstanding, are comparable to those reported for other FAD-dependent oxidoreductases acting on relatively small substrates. For example, glycine oxidase from *B. licheniformis* (ThiO, Table 1) was reported to oxidize glycine with a k_cat_/K_M_ of 340 M^−1^s^−1^ and with a K_M_ of 0.9 mM^37^; whereas the monomeric sarcosine oxidase from *Bacillus* sp. *B-0618* (SoxA, Table 1) oxidized sarcosine with a k_cat_/K_M_ of 10,100 M^−1^s^−1^ and a K_M_ of 4.5 mM.^41^

PbfC showed the highest apparent catalytic efficiency in the oxidation of M_1_AEP and, in agreement with the semi-quantitative results of the microtiter assays, PbfC also showed the highest discrimination toward non-methylated AEP (400-fold difference in catalytic efficiency), which seemed mostly due to a weaker substrate binding (100-fold difference in K_M_). In comparison, PbfD2 showed only a 4-fold difference in k_cat_/K_M_ values between M_1_AEP and plain AEP, while PbfD1 specificity was somewhat intermediate, with an 18-fold discrimination toward AEP.

While the experiments earlier refer to naturally occurring aminophosphonates, we also performed assays using *N*-ethyl-AEP and *N*-propyl-AEP, which are not known to occur in nature. The results, shown in Figure S8, confirm in general a dichotomy between the rather substrate-specific PbfC and the less strict PbfD enzymes.

### Evidence that the PbfB enzymes also oxidize M_1_AEP

As stated previously, we could not obtain the recombinant PbfB from *V. vulnificus* in pure form. Therefore, to test its activity, we resorted to performing assays on the extracts of *E. coli* cells that expressed this enzyme. In this case, however, assays based on the formation of H_2_O_2_ (Figure 3), were deemed unreliable because other enzyme activities in the extracts (catalases, for example) could curb the accumulation of the expected products. Therefore, considering that inorganic phosphate is a much more stable end-product, we opted initially for a microtiter assay based on the BIOMOL Green assay kit.

**Figure 5:**
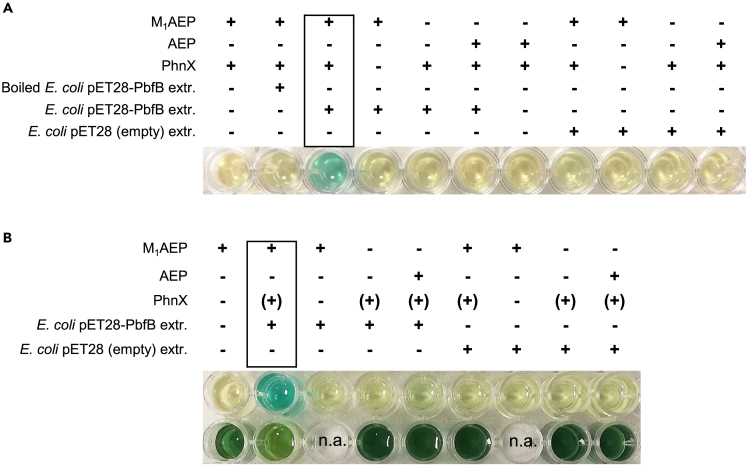
Evidence for M_1_AEP degradation by soluble extracts of *E. coli* cells expressing PbfB (A) Phosphate release assay. Cultures (10 mL) of pGro7/BL21 cells containing either the pET28-PbfB plasmid or an empty pET28 vector were induced with 1 mM IPTG overnight (20°C), then harvested, resuspended in 2 mL of HEPES buffer pH 7.5 and homogenized by sonication. A part of the soluble extract from PbfB-expressing cells was heated for 10 min at 100°C, as a control. Extracts (40 μL) were subsequently incubated in 100 μL (final volume) of 50 mM TEA-HCl buffer pH 8.0 containing 2 mM MgCl_2_ and (when indicated) 4 μM PhnX and 5 mM M_1_AEP or AEP. After 1 h at room temperature, 36 μL of the reaction mixture was transferred into 200 μL of BIOMOL Green reagent. Color development was read after ∼30 min. (B) A partial repetition of the phosphate release assay (upper row) performed on a different batch of cell extract and conducted in parallel with a DCPIP reduction assay (lower row). Wells in the lower row contained 200 μM DCPIP, 3 mM PMS, and (when indicated) 5 mM substrate (M_1_AEP or AEP) and 15 μL cell extract in a total volume of 100 μL. PhnX and MgCl_2_ were consistently omitted for the assays in the lower row.

The rationale was that if the extract contained any activity capable of producing PAA from M_1_AEP, coupling this reaction with the PhnX reaction would result in the eventual accumulation of phosphate. As shown in Figure 5A, this was exactly the case.

Phosphate accumulation was dependent on the simultaneous presence in the reaction mixture of the cell extract, of PhnX and of M_1_AEP, consistent with the rationale of the assay. No accumulation of phosphate was detected when the extract came from *E. coli* cells not expressing PbfB, or when the extract of PbfB-expressing cells had been inactivated by boiling (Figure 5A). No accumulation of phosphate was observed when M_1_AEP was replaced by plain AEP, suggesting a certain substrate specificity of the process. Finally, when cell extracts were used in a DCPIP-reduction assay (analogous to the one depicted in Figure 3) the strongest signal occurred when both the extract from PbfB-expressing cells and M_1_AEP were present (Figure 5B).

Overall, these data were fully consistent with the hypothesis that PbfB from *V. vulnificus* catalyzes the oxidation of M_1_AEP, much like the other oxidoreductases described in this work. The activity detected in extracts of PbfB-expressing cells apparently took place both in the presence and in the absence of electron-accepting dyes (Figure 5B); however, the limits of the assays conducted on whole cell extracts do not allow one to conclude whether PbfB behaves as an oxidase (like the PbfD enzymes) or as a dehydrogenase (like PbfC).

## Discussion

### FAD-dependent enzymes from AEP degradation clusters serve to oxidize M_1_AEP

Different studies have highlighted the role of phosphonate degradation from an ecological standpoint (in the frame of the global biogeochemical P-cycling) and as a tool that bacteria employ for surviving in nutrient-limited ecological niches. At least three types of phosphonate-degrading pathways have been identified so far, schematically distinguished based on the mechanism by which the C-P bond is ultimately cleaved—i.e., through either a hydrolytic, radical, or oxidative reaction.^7^^,^^14^^,^^49^^,^^50^^,^^51^^,^^52^ However, except for the very flexible but complex C-P-lyase system,^52^^,^^53^ these pathways appear to be very narrow in substrate range, being specialized for the degradation of one particular compound.

The hydrolytic pathways for AEP degradation are a case in point. While AEP is indeed the most abundant natural phosphonate,^5^^,^^6^^,^^16^ the known pathways shown in Figure 1 are by themselves unable to process other AEP-related phosphonates that abound in the environment. As noted in the introduction, one evolutionary workaround could be the co-option of accessory enzymes able to convey additional compounds into the AEP hydrolytic pathways.

This study deals with the recurrent presence of genes encoding putative (and only weakly interrelated) FAD enzymes within bacterial clusters for AEP degradation. With “recurrent,” we refer to genes that are repeatedly found in such clusters—but whose presence is neither constant nor limited to a specific cluster type. The optional but frequent occurrence of these genes is very consistent with them encoding ancillary enzymes, acting on compounds related to the main substrate of the pathway. Establishing the precise function of these accessory enzymes, however, is often not straightforward. For example, to convincingly establish the specificity of the oxidoreductases described in this work we had to test a variety of phosphonates^54^ and this required in particular the chemical synthesis of numerous *N*-alkylated derivatives of AEP and *(R)*-HAEP, by either newly developed or adapted literature methods. M_1_AEP and M_2_AEP were obtained by reaction of diethyl 2-bromoethylphosphonate with the corresponding mono- and dialkylated amines, followed by acidic deprotection. M_3_AEP became accessible from diethyl 2-(dimethylamino)-ethylphosphonate by alkylation with methyl iodide following an adapted literature procedure.^55^ The trimethylated analogue (*R*)-M_3_HAEP was obtained from (*R*)-HAEP by methylation, while the (*R*)-M_2_HAEP was accessible by an Eschweiler-Clark approach. The monomethylated derivative (*R*)-M_1_HAEP was accessible from (*R*)-diisopropyl (2-(1,3-dioxoisoindolin-2-yl)-1-hydroxyethyl)phosphonate^18^ by using a tosylated intermediate to control the degree of methylation.

The possible role of some of the FAD oxidoreductases described herein was tentatively hinted at by their genomic annotations, but such annotations, when present, remained vague and unsubstantiated. For example, PbfD1 from *A. baumannii* is annotated in GenBank as a member of the TIGR03364 family (see Table S1), whose description reads “FAD dependent oxidoreductases […] syntenically associated with a family of proposed phosphonatase-like enzymes […] A likely role for this enzyme involves the oxidative deamination of an aminophosphonate differing slightly from AEP, possibly 1-hydroxy-2-aminoethylphosphonate” (that is, HAEP).

We have now provided strong evidence (in the case of PbfB) or complete proof (for the PbfC and PbfD proteins) that these FAD enzymes catalyze the oxidative deamination of the *N*-monomethylated form of AEP (M_1_AEP), generating PAA that can be then hydrolyzed by PhnX to obtain acetaldehyde and inorganic phosphate.

This activity seems biologically well justified: M_1_AEP is frequently found in nature (where its abundance can sometimes approach that of AEP^28^^,^^31^) but it cannot serve as a substrate for PhnW and therefore it cannot directly enter the pathways shown in Figure 1. Thus, collectively, the PbfB, PbfC, and PbfD enzymes serve to funnel M_1_AEP into the hydrolytic AEP degradation pathways, allowing the bacteria to consume different aminophosphonates and presumably improving organismal fitness under different nutrient conditions.

### Functional diversity of the PbfC and PbfD enzymes

Even though all the oxidoreductases described in this work share the ability to oxidize M_1_AEP, their mechanistic features are remarkably distinct. The PbfC enzyme from *Azospirillum* sp. was peculiar in its strong discrimination against plain AEP, which was oxidized ∼400-fold less efficiently than its *N*-methylated counterpart (comparison is based on k_cat_/K_M_ values^56^^,^^57^; Figure 4G and Table 2). This preference even exceeds the calculated maximum discrimination against suboptimal substrates that lack a methyl group (the theoretical limit would be a ∼160-fold discrimination^58^^,^^59^) but in this case the methyl group is directly attached to the nitrogen undergoing reaction and this is expected to change some properties of the amino group, like its pK_a_.

Notably, PbfC proved very inefficient in using O_2_ as the immediate electron acceptor, whereas its reaction proceeded quickly in the presence of the artificial electron acceptor DCPIP. This suggests that PbfC should be formally classified as a dehydrogenase and we propose for this enzyme the systematic name *N*-methyl-2-aminoethylphosphonate dehydrogenase. Physiologically, it is not clear what the electron acceptor could be. NAD^+^ and NADP^+^ did not apparently accept electrons from this enzyme (data not shown), so the most obvious possibilities would be a quinone (in *Azospirillum* species, ubiquinone Q-10 is reportedly the major respiratory quinone^60^) or perhaps some carrier protein, as for the mammalian sarcosine dehydrogenase (SarDH, Figure S4).^42^

In contrast to PbfC, the PbfD enzymes showed a similarly efficient reaction in the presence of either O_2_ or DCPIP-PMS suggesting that these enzymes should be classified as oxidases (even though we cannot rigorously exclude that an electron acceptor better than oxygen may exist *in vivo*). Our data hint that the PbfB-type enzymes can carry out the M_1_AEP oxidation in the absence of acceptor dyes (Figure 5); however, our inability to obtain PbfB in pure form prevents any firm conclusion on the formal classification of this enzyme as an oxidase or dehydrogenase.

A further distinctive feature of PbfD1 and PbfD2 is that they exhibited a less pronounced specificity for M_1_AEP. In particular, PbfD1 was unique in showing an appreciable activity toward *N,N*-dimethyl-AEP (M_2_AEP), through a reaction somehow resembling that of dimethylglycine oxidase (also belonging to the PF01266 group).^61^ The PbfD1-catalyzed oxidation of M_2_AEP was just 6-fold less efficient than the oxidation of M_1_AEP (Table 2) and could perhaps have some physiological relevance. Small amounts of M_2_AEP have been occasionally isolated from marine organisms (e.g., ref.^28^); furthermore, M_2_AEP may conceivably be an intermediate in the biosynthesis of M_3_AEP, which is another AEP analog frequently found in nature. Notably, M_3_AEP itself was not a substrate for any of the oxidoreductases described in this study. Indeed, there are no FAD-dependent enzymes reported to act on quaternary amines, while the only M_3_AEP degradation pathway described to date involves two iron-dependent oxygenases and produces trimethylglycine in addition to phosphate.^62^

More relevant from a biological viewpoint was the ability of PbfD2 from *M. fucicola* to react with AEP only 4-fold slower than with M_1_AEP (Figure 4I and Table 2). Such a finding implies that *in vivo* this enzyme could be performing a double duty, generating PAA not only from M_1_AEP but also from AEP. Through this latter reaction, PbfD2 would in effect surrogate the function of PhnW, whose gene is absent in the *M. fucicola* gene cluster (Figure S1) and apparently from the whole *M. fucicola* genome. Altogether, these observations strongly suggest that enzymes like PbfD2 may contribute to “novel” versions of the hydrolytic pathways, in which PAA is generated by the oxidative deamination of AEP rather than by transamination (see in the following section).

### On the evolution of M_1_AEP degrading enzymes

The heterogeneity of the FAD-dependent enzymes analyzed in this study, both in terms of sequence and mechanistic details, outlines a remarkable instance of convergent evolution. It suggests that evolution has “invented” an ancillary M_1_AEP oxidoreductase at least three times, co-opting different ancestor enzymes to perform the task. PbfB sequences might have emerged last, as they are the less divergent (Figure 2) and their distribution is mostly limited to *Betaproteobacteria* and *Gammaproteobacteria* (Table S1 and Figure S3). On the other hand, PbfC homologs associated with AEP degradation clusters were found in *Alphaproteobacteria, Gammaproteobacteria* (where they were widespread), and *Betaproteobacteria*, but also frequently observed in bacteria belonging to the FCB group and occasionally in Terrabacteria. The PbfD group of enzymes might have been the most ancient to emerge: this is suggested both by the large divergence between the sequences of these enzymes (the two PbfD enzymes tested in this work, for example, show only a ∼30% identity) and by the relatively broad distribution among bacterial phyla. PbfD homologs are encoded in AEP degradation clusters in *Alphaproteobacteria*, *Betaproteobacteria, Gammaproteobacteria*, and bacteria from the FCB and *Planctomycetota, Verrucomicrobiota*, and *Chlamydiota* (PVC) groups (mostly *Planctomycetota*). Notably, PbfD homologs not associated with *phnWX/phnWYA* clusters were also detected in many Terrabacteria (mostly cyanobacteria).

The varying presence of PbfB, PbfC, and PbfD enzymes in closely related organisms is consistent with a significant degree of horizontal gene transfer involving these pathways. For example, *Rhodoferax sediminis* was found to possess *pbfB-phnWYA* genes, while the closely related *Rhodoferax koreense* possesses *pbfD-phnX* instead (Table S1). Additionally, the similarities between genes in Figure 2 (and Figure S3) are not congruent with the phylogeny of the organisms which possess them; e.g., the PbfD1 clade of Figure 2 consists of sequences from diverse phyla, which are more similar to each other than to sequences from similar phyla in the rest of the PbfD group. The horizontal transfer of these accessory genes is consistent with the previously inferred horizontal transfer of the “core” genes of the hydrolytic pathways (*phnW* and *phnX* for example), and it is logical that these would move between organisms as a part of complete clusters rather than separately.^63^

### Biological significance of M_1_AEP degradation

Overall, the oxidoreductases described here occur in all the major clades of PhnA and PhnX-possessing bacteria observed in earlier database searches, i.e., predominantly proteobacteria, with some representatives of the PVC group^14^^,^^64^ (Figure 2 and Table S1). These organisms have very diverse habitat ranges, as they include species from predominantly aquatic (e.g., *Vibrio* spp.^65^) and soil habitats (e.g., *Azospirillum* spp.^66^), as well as cosmopolitan species (e.g., *Acinetobacter* spp.^67^). The inter-genomic distribution of clusters for AEP degradation, together with isolation of AEP-consuming bacteria from both aquatic and terrestrial environments (e.g., refs.^68^^,^^69^^,^^70^) have been taken as evidence that AEP degradation is advantageous across a wide range of habitats. Our data hint that this may also be true for M_1_AEP degradation, although *in vivo* studies will be required to fully establish this point.

The most obvious advantage of AEP degradation is the retrieval of inorganic phosphate. However, previous research has shown that AEP degradation (via the PhnWX or PhnWYA pathways) can sustain marine microbes as a source of nitrogen even when phosphate levels are high.^70^ The degradation of M_1_AEP carried out by PbfC and PbfD enzymes generates methylamine as a product (Figure S6) and in all likelihood this is also true for PbfB. Methylamine is reportedly used as a nitrogen source by a diverse range of *Alpha* and *Gammaproteobacteria*,^71^ suggesting that hydrolytic pathways that include the oxidoreductases could support growth on M_1_AEP as a nitrogen source. While phosphate-insensitive expression of oxidoreductase-containing pathways has yet to be experimentally demonstrated, this would be consistent with these enzymes broadening the range of aminophosphonates which contribute to marine phosphorus, nitrogen, and possibly carbon biogeochemistry.

Finally, it is also possible to hypothesize that some of the oxidoreductase enzymes may be involved in the host-interactions of microbial species. For example, the *Vibrio* spp. that possess *pbfB* (Figure S3) are closely associated, possibly as symbionts, with marine gastropods which synthesize sphingophosphonolipids containing M_1_AEP.^29^^,^^72^ Alternatively, as these *Vibrio* spp. are also pathogens of mussels and other marine invertebrates that produce lipid-bound M_1_AEP, the PbfB enzymes may play a role in the degradation of host lipids during pathogenesis.^32^^,^^73^

### AEP degradation à la carte

While the FAD-dependent oxidoreductases expand the versatility of the hydrolytic pathways for the degradation of AEP, our data also strongly suggest that some of these enzymes participate in novel versions of such pathways, in which PAA is not generated from AEP by transamination, but rather by oxidative deamination.

**Figure 6:**
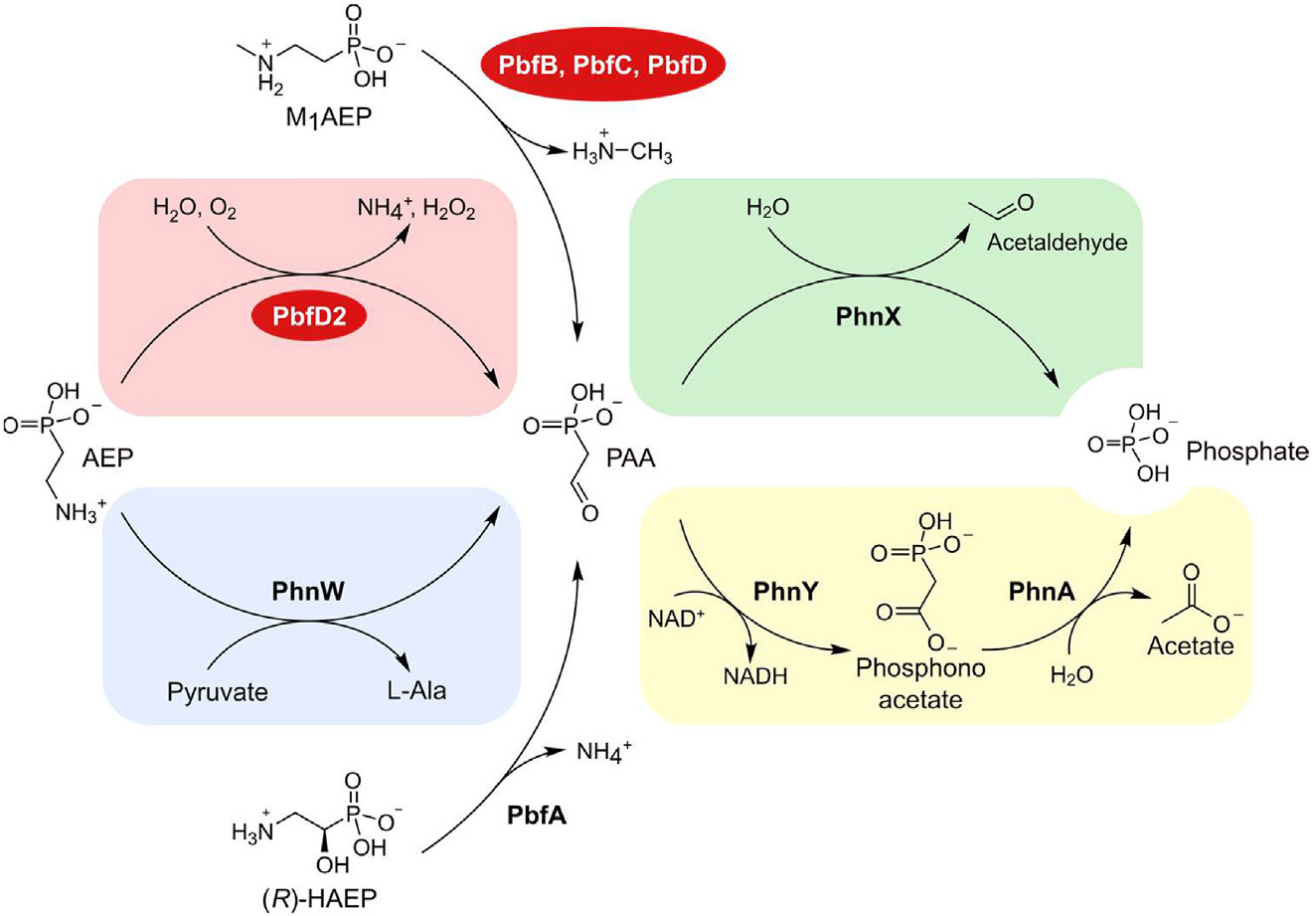
A schematic, updated summary of the different possible pathways for the hydrolytic degradation of AEP and related compounds The newly identified enzymes described in this work are highlighted in red. In the oxidation of M_1_AEP the electron acceptor is not indicated for simplicity; as detailed in this work, the acceptor is O_2_ for PbfD enzymes, but unknown for PbfC and PbfB. The enzyme catalyzing the oxidative deamination of AEP is indicated as PbfD2 by approximation. Indeed, most of the genes of the *pbfD2* subgroup belong to clusters that do not include *phnW*, but there are exceptions; at the same time, a minority of *pbfD* genes outside the *pbfD2* subgroup localizes in clusters that lack *phnW* (see Table S1 for examples).

Figure 6 provides an updated overview of the different possible hydrolytic pathways for the degradation of AEP and related compounds. All of these pathways center on the common intermediate PAA, which can be formed or degraded through different enzymes, thus providing four different pathways for AEP catabolism (Figure 6). The first is the classic PhnWX pathway (reactions highlighted in light blue and light green, Figure 6), as found for example in *Azospirillum* sp. B510, where it is enriched by the activities of PbfA and PbfC (Figure S1). The second is the PhnWYA pathway (in Figure 6, reactions highlighted in light blue and yellow), as found in *Burkholderia multivorans*. A third option is a pathway based on PbfD2 and PhnX (upper set of reactions in Figure 6, highlighted in light red and light green), as observed in *M. fucicola*. Finally, a fourth pathway based on PbfD2, PhnY, and PhnA is also possible, as found in *Luteolibacter luteus* (Figure S1). Notably, there are no enzymes in common between the second pathway and the third, nor between the first and the fourth.

The distribution of the four pathways among different bacteria may be an accident of evolution. Nevertheless, it should also be noted that certain pathways may suit better the lifestyles and metabolic setups of individual bacterial species. For example, a pathway based on PhnWYA requires three enzymes and is unable to process M_1_AEP, but is compatible with an anaerobic lifestyle and it generates only harmless products. On the other hand, a pathway based on PbfD2 and PhnX employs only two enzymes and is more versatile, but it can only function under aerobic conditions and it generates two reactive products (hydrogen peroxide and acetaldehyde) that will need to be further processed by other metabolic enzymes.

### Limitations of the study

The functional characterization described herein was conducted *in vitro*, on recombinant proteins expressed in *E.coli*. It could be useful to complement our results with *in vivo* approaches, encompassing for example the deletion of the *pbfB*, *pbfC*, or *pbfD* genes in organisms that possess them. Such an approach might, however, be limited by several confounding factors and requires an understanding of the conditions under which the AEP degradation gene cluster is expressed in that organism. Furthermore, while our data strongly indicate that PbfB is involved in the processing of M_1_AEP, details about its exact reaction remain uncertain because we did not succeed in purifying the protein. In particular, the limits of assays conducted on whole cell extracts do not allow us to conclude whether PbfB behaves as an oxidase (like the PbfD enzymes) or as a dehydrogenase (like PbfC).

## STAR★Methods

### Key resources table

**Table:** 

REAGENT or RESOURCE	SOURCE	IDENTIFIER
**Bacterial and virus strains**		

*E. coli* Tuner™ (DE3) cells	Novagen (Merck)	70623
*E. coli* Chaperone Competent Cells pGro7/BL21	Takara Bio Inc.	9122

**Chemicals, peptides, and recombinant proteins**		

Alcohol dehydrogenase (baker’s yeast)	Sigma	A7011
L-glutamate dehydrogenase (Bovine liver)	Sigma	G2501
Horseradish peroxidase	Sigma	P-8125
Triethanolamine (TEA)	Sigma	90279
2-Aminoethylphosphonic acid (AEP)	Wako Chemicals	326-22153
3-Phosphono-DL-alanine	Sigma	A-4910
3-aminopropylphosphonic acid	Santa Cruz Biotechnology	sc-251943
Glyphosate	Aldrich	337757
Sarcosine	Fluka	84530
Glycine	Sigma	G8898
D-Alanine	Sigma	A7377
Taurine	Fluka	86330
Beta-Alanine (β-Ala)	Aldrich	14,606-4
Flavin adenine dinucleotide (FAD)	Sigma	F6626
Riboflavin 5′-monophosphate (FMN)	Fluka	83810
NADH	Alfa-Aesar	J61638
BIOMOL® Green kit	ENZO Biosciences	BML-AK111
2,6-Dichlorophenolindophenol Na salt (DCPIP)	Aldrich	119814
Phenazine methosulfate (PMS)	Sigma	P-9625
o-Dianisidine hydrochloride	Sigma	D-3252

**Recombinant DNA**		

Plasmid pET28a-PhnX (*V. splendidus*)	Zangelmi et al.^23^	n.a.
Plasmid pET28a-PbfB (*V. vulnificus*)	This paper	n.a.
Plasmid pET28a-PbfC (*Azospirillum*)	This paper	n.a.
Plasmid pET28a-PbfD1 (*A. baumannii*)	This paper	n.a.
Plasmid pET28a-PbfD2 (*M. fucicola*)	This paper	n.a.

**Software and algorithms**		

Microbesonline	Dehal et al.^74^	http://www.microbesonline.org/
IMGM/M	Chen et al.^75^	https://img.jgi.doe.gov/cgi-bin/m/main.cgi
pBLAST (NCBI)	Altschul et al.^76^	https://blast.ncbi.nlm.nih.gov/Blast.cgi
MUSCLE algorithm	Edgar^77^	https://www.drive5.com/muscle/
IQTREE 1.6.12	Minh et al.^78^	http://www.iqtree.org/release/v1.6.12
ModelFinder	Kalyaanamoorthy et al.^79^	N/A
FigTree 1.4.4	Andrew Rambaut, University of Edinburgh	http://tree.bio.ed.ac.uk/
SigmaPlot 14.0	Systat Software	N/A
MestReNova v12.0.4	Mestrelab Research	N/A

### Resource availability

#### Lead contact

Further information and requests for resources and reagents should be directed to and will be fulfilled by the lead contact, Alessio Peracchi (alessio.peracchi@unipr.it).

#### Materials availability

Materials generated in this study are available upon request to the lead contact.

### Experimental model and study participant details

This study did not include *in vivo* experiments. Our experimental model was constituted by recombinant proteins expressed in *E. coli*.

### Method details

#### Chemical synthesis of phosphonate compounds

Racemic HAEP was synthesized starting from vinylphosphonic acid, as described in the literature^18^; the *(S)-* and *(R)* enantiomers of HAEP were also prepared by methods described in the same publication.^18^

The synthesis of the different *N-*alkylated analogs of AEP and *(R)*-HAEP was accomplished by either newly developed or adapted literature methods, employing the following general procedures.

NMR spectra were recorded on a Bruker BioSpin AV III HD 700 (^1^H: 700.40 MHz, ^13^C: 176.12 MHz), AV III 600 (^1^H: 600.25 MHz, ^13^C: 150.93 MHz, ^31^P: 242.99 MHz), AV NEO 400 (^1^H: 400.27 MHz, ^31^P: 162.03 MHz) or AV NEO NanoBay 400 (^1^H: 400.13 MHz, ^31^P: 161.98 MHz). Chemical shifts were referenced to solvent (residual) peaks of CDCl_3_: *δ* = 7.26 ppm (^1^H NMR), *δ* = 77.00 ppm (^13^C NMR); CD_3_OD: *δ* = 3.31 ppm (^1^H NMR), *δ* = 49.00 ppm (^13^C NMR); HDO: *δ* = 4.79 ppm (^1^H NMR). ^31^P NMR spectra were referenced to external H_3_PO_4_ (85% aqueous solution), *δ* = 0.00 ppm. Chemical shifts (*δ*) are given in ppm and coupling constants (*J*) in Hz. Spectroscopic data for all synthesized compounds are provided in the Supporting Information. Mass spectra were recorded on a Bruker maXis UHR-TOF (instrument type: Qq-TOF), the samples were ionized by electrospray ionization (ESI). IR spectra were recorded on a Bruker Vertex 70 IR spectrometer in ATR mode. Optical rotations were measured at 20°C on a Perkin-Elmer 341 polarimeter in a 1 dm cell. [α]_D_ values are given in 10^-1^ deg cm^2^ g^-1^. Melting points were determined on a Büchi Melting Point-system M-560 instrument or a Reichert-Jung Thermovar hot-stage microscope and are uncorrected. NMR spectra for all synthesized compounds, are provided in the Data S1.

Thin layer chromatography (TLC) was carried out using Merck silica gel 60 F_254_ glass plates (coating 0.25 mm thick) and spots were visualized by UV light (254 nm) and/or dipping the plate in a Cer-Ammonium-Molybdate (CAM) solution [23 g (NH_4_)_6_Mo_7_O_24_ × 4 H_2_O, 1 g Ce(SO_4_)_2_ × 4 H_2_O in 500 mL 10% aqueous H_2_SO_4_], a KMnO_4_ solution (9 g KMnO_4_, 60g K_2_CO_3_, 15 mL 5% aq. NaOH in 900 mL H_2_O) or a ninhydrin solution [0.5% ninhydrin in ethanol (98%v/v)], followed by heating with a heat gun. Chromatographic purifications were either performed manually or carried out on a fully automated Biotage Isolera Prime flash purification system using KP-Sil separation cartridges 10-100 g (depending on the total amount of substance), filled with Merck silica gel 60 (230-400 mesh).

All mentioned chemicals and solvents were bought from ABCR, Acros, Fluka, Sigma-Aldrich or TCI and were used without any further purification, unless stated differently.

#### Synthesis of monoalkylated AEP derivatives

##### Diethyl-(2-bromoethyl)phosphonate (1)

Related to Scheme S1. Diethyl-(2-bromethyl)phosphonate (**1**) has been prepared by an adapted literature procedure.^80^ Triethyl phosphite (5.6 mmol, 0.96 mL, 1 eq.) and 1,2-dibromoethane (22.4 mmol, 1.93 mL, 4 eq.) were stirred 4 h at 160°C followed by evaporation of the volatile components on a rotary evaporator. The crude product was purified by vacuum distillation (3 torr, 80°C), giving diethyl-(2-bromethyl)phosphonate (**1**, 1.152 g, 4.7 mmol, 84%) as a colorless liquid. ^1^H NMR (600.25 MHz, CDCl_3_) *δ* = 4.17-4.06 (m, 4H, 2 × O-CH_2_), 3.53 (td, ^3^*J*_H,H_ 8.5, ^3^*J*_H,P_ 8.4, 2H, CH_2_-Br), 2.38 (td, ^2^*J*_H,P_ 18.5, ^3^*J*_H,H_ 8.5, 2H, CH_2_-P), 1.33 (t, ^3^*J*_H,H_ 7.1, 6H, 2 × CH_3_); ^13^C NMR (150.93 MHz, CDCl_3_) *δ* = 62.02 (d, ^2^*J*_C,P_ 6.5, 2 × O-CH_2_), 30.79 (d, ^1^*J*_C,P_ 134.6, CH_2_-P), 23.83 (s, CH_2_-Br), 16.41 (d, ^3^*J*_C,P_ 6.0, 2 × CH_3_); ^31^P NMR (162.03 MHz) *δ* = 25.57 (s); HRMS calculated for [C_6_H_14_O_3_PBr+Na]^+^: 266.9762, found: 266.9759; IR (ATR) *v* = 2981, 2907, 1273, 1016, 955, 786, 509 cm^-1^.

All three monoalkylated phosphonic acids (**5**-**7**) were obtained following the same two-step procedure from **1** as outlined below (**procedure A**, followed by **procedure B**). For **procedure A**, either methyl amine (to obtain **2**), ethyl amine (to obtain **3**) or *n*-propyl amine (to obtain **4**) was used as monoalkylamine component. Methylamine was used as aqueous solution (40% w/w in water), thus the total volume of added ethanol was reduced to 4.7 mL.

##### Procedure A

The respective monoalkylamine (12 eq.) was added at 0°C to a solution of diethyl-(2-bromoethyl)phosphonate (**1**, 4.7 mmol, 1.152 g, 1 eq.) in dry EtOH (10 mL), and the resulting mixture was stirred for 3 h at room temperature. The solvent was removed under reduced pressure to give diethyl 2-alkylamino-ethylphosphonates (**2**–**4**, partially as their hydrobromide), in sufficient purity for the next step.

##### Diethyl 2-methylamino-ethylphosphonate (2, partially as its hydrobromide)

Related to Scheme S1. ^1^H NMR (700.40 MHz, CDCl_3_) *δ* = 4.14-4.03 (m, 4H, 2 × O-CH_2_) 2.85 (dt, ^3^*J*_H,P_ 15.0, ^3^*J*_H,H_ 7.3, 2H, CH_2_-N), 2.42 (s, 3H, N-CH_3_), 1.96 (dt, ^2^*J*_H,P_ 18.4, ^3^*J*_H,H_ 7.3, 2H, CH_2_-P), 1.94 (s, 1H, NH), 1.31 (t, ^3^*J*_H,H_ 7.1, 6H, 2 × O-CH_2_-CH_3_); ^13^C NMR (176.12 MHz, CDCl_3_) *δ* = 61.56 (d, ^2^*J*_C,P_ 6.6, 2C, 2 × O-CH_2_), 45.28 (d, ^2^*J*_C,P_ 3.5, 1C, CH_2_-N), 35.9 (s, NCH_3_) 26.17 (d, ^1^*J*_C,P_ 139.6, C-P), 16.40 (d, ^3^*J*_C,P_ 6.0, 2C, 2 × O-CH_2_-CH_3_); ^31^P NMR (162.03 MHz, CDCl_3_) *δ* = 30.49 (s); HRMS calculated for [C_7_H_18_NO_3_P+H]^+^: 190.1103, found: 190.1100; IR (ATR) *v* = 3312, 2980, 2794, 1230, 1021, 788, 538 cm^-1^.

##### Diethyl 2-ethylamino-ethylphosphonate (3, partially as its hydrobromide)

Related to Scheme S1. ^1^H NMR (600.25 MHz, CDCl_3_) *δ* = 4.17-4.03 (m, 4H, 2 × O-CH_2_), 2.91 (td, ^3^*J*_H,P_ 14.8, ^3^*J*_H,H_ 7.3, 2H, CH_2_-CH_2_-P), 2.66 (q, ^3^*J*_H,H_ 7.1, 2H, N-CH_2_-CH_3_), 1.98 (dt, ^2^*J*_H,P_ 18.2, ^3^*J*_H,H_ 7.3, 2H, CH_2_-P), 1.65 (brs, 1H, NH), 1.32 (t, ^3^*J*_H,H_ 7.1, 6H, 2 × CH_3_-CH_2_-O), 1.11 (t, ^3^*J*_H,H_ 7.1, 3H, N-CH_2_-CH_3_); ^13^C NMR (150.93 MHz, CDCl_3_) *δ* = 61.59 (d, ^2^*J*_C,P_ 6.5, 2C, 2 × O-CH_2_), 43.73 (s, N-CH_2_-CH_3_), 43.15 (d, ^2^*J*_C,P_ 3.6, CH_2_-CH_2_-P), 26.46 (d, ^1^*J*_C,P_ 139.3, CH_2_-P), 16.44 (d, ^2^*J*_C,P_ 6.0, 2C, 2 × CH_3_-CH_2_-O), 15.13 (s, N-CH_2_-CH_3_); ^31^P NMR (161.98 MHz) *δ* = 27.07 (s); HRMS calculated for [C_8_H_20_NO_3_P+H]^+^: 210.1254, found: 210.1267.

##### Diethyl 2-propylamino-ethylphosphonate (4, partially as its hydrobromide)

Related to Scheme S1. ^1^H NMR (600.25 MHz, CDCl_3_) *δ* = 4.15-4.03 (m, 4H, 2 × O-CH_2_), 2.89 (td, ^3^*J*_H,P_ 14.8, ^3^*J*_H,H_ 7.3, 2H, CH_2_-CH_2_-P), 2.56 (t, ^3^*J*_H,H_ 7.3, 2H, N-CH_2_-CH_2_-CH_3_), 1.97 (dt, ^2^*J*_H,P_ 18.3, ^3^*J*_H,H_ 7.3, 2H, CH_2_-P), 1.64 (brs, 1H, NH), 1.49 (sex, ^3^*J*_H,H_ 7.3, N-CH_2_-CH_2_-CH_3_), 1.31 (t, ^3^*J*_H,H_ 7.1, 6H, 2 × CH_3_-CH_2_-O), 0.91 (t, ^3^*J*_H,H_ 7.4, 3H, N-CH_2_-CH_2_-CH_3_); ^13^C NMR (150.93 MHz, CDCl_3_) *δ* = 61.57 (d, ^2^*J*_C,P_ 6.4, 2C, 2 × O-CH_2_), 51.48 (s, N-CH_2_-CH_2_-CH_3_), 43.31 (d, ^2^*J*_C,P_ 3.5, CH_2_-CH_2_-P), 26.53 (d, ^1^*J*_C,P_ 139.1, CH_2_-P), 23.10 (s, CH_2_-CH_2_-N), 16.46 (d, ^3^*J*_C,P_ 6.0, 2C, 2 × CH_3_-CH_2_-O), 11.75 (s, N-CH_2_-CH_2_-CH_3_); ^31^P NMR (161.98 MHz, CDCl_3_) *δ* = 27.53 (s); HRMS calculated for [C_9_H_22_NO_3_P+H]^+^: 224.1410, found: 224.1418.

##### Procedure B

The respective diethyl 2-alkylamino-ethylphosphonate (**2**-**4**) was dissolved in hydrochloric acid (6 M in H_2_O, 12 mL) and stirred at 120°C for 6 h. The crude product was concentrated *in vacuo* and purified by ion exchange chromatography (Dowex 50W × 8 / H^+^-form) using H_2_O as eluent. The product containing fractions (ninhydrin positive) were pooled and the solvent was removed under reduced pressure to yield (2-alkylamino-ethyl phosphonates **5**-**7**, which could be crystallized from H_2_O/EtOH to give colorless needles.

##### 2-*N*-Methylamino-ethylphosphonic acid (M_1_AEP; 5, 98% over two steps)

Related to Scheme S1. m.p. 279-282°C; ^1^H NMR (700.40 MHz, D_2_O) *δ* = 3.24 (dd, ^3^*J*_H,P_ 16.0, ^3^*J*_H,H_ 7.6, 2H, CH_2_-N), 2.75 (d, ^3^*J*_H,H_ 1.9, 3H, CH_3_), 2.03-1.96 (m, 2H, CH_2_-P); ^13^C NMR (176.12 MHz, D_2_O) *δ* = 44.85 (s, CH_2_-N), 32.46 (s, CH_3_), 24.90 (d, ^1^*J*_C,P_ 131.7, CH_2_-P); ^31^P NMR (161.98 MHz, D_2_O) *δ* = 18.23 (s); HRMS calculated for the dimer [C_6_H_20_N_2_O_6_P_2_+H]^+^: 279.0869, found: 279.0854; anal. calc. for C_3_H_10_NO_3_P × 0.1 H_2_O: C 25.57%, H 7.30%, N 9.94%, O 35.20%, P 21.98%, found: C 25.82%, H 7.44%, N 9.91%, O 34.15%, P 21.79%; IR (ATR) *v* = 3102, 2694, 2385, 1681, 1586, 1133, 918, 443 cm^-1^.

##### 2-*N*-Ethylamino-ethylphosphonic acid (*N*-ethyl-AEP; 6, 85% over two steps)

Related to Scheme S1. m.p. 287-288°C; ^1^H NMR (600.25 MHz, D_2_O) *δ* = 3.27-3.21 (m, 2H, CH_2_-CH_2_-P), 3.13 (q, ^3^*J*_H,H_ 7.3, 2H, CH_2_-CH_3_), 2.03-1.94 (m, 2H, CH_2_-P), 1.30 (t, ^3^*J*_H,H_ 7.3, 3H, CH_3_); ^13^C NMR (150.93 MHz, D_2_O) *δ* = 42.82 (s, CH_2_-CH_2_-P), 42.56 (s, CH_2_-CH_3_), 25.09 (d, ^1^*J*_C,P_ 131.6, CH_2_-P), 10.50 (s, CH_3_); ^31^P NMR (162.02 MHz, D_2_O) *δ* = 18.16 (s); HRMS calculated for [C_4_H_12_NO_3_P+Na]^+^: 176.0447, found: 176.0444; anal. calc. for C_4_H_12_NO_3_P: C 31.38%, H 7.90%, N 9.15%, O 31.35%, P 20.23%, found: C 31.15%, H 8.11%, N 9.21%, O 31.53%, P 19.03%; IR (ATR) *v* = 2993, 2857, 2733, 2433, 2338, 1711, 1139, 1020, 916 cm^-1^.

##### 2-*N*-Propylamino-ethylphosphonic acid (*N*-ethyl-AEP; 7, 90% over two steps)

Related to Scheme S1. m.p. 274°C (decomp.); ^1^H NMR (600.25 MHz, D_2_O) *δ* = 3.28-3.28 (m, 2H, CH_2_-CH_2_-P), 3.04 (t, ^3^*J*_H,H_ 7.5, 2H, N-CH_2_-CH_2_-CH_3_), 2.03-1.95 (m, 2H, CH_2_-P), 1.49 (sex, ^3^*J*_H,H_ 7.5, N-CH_2_-CH_2_-CH_3_), 0.99 (t, ^3^*J*_H,H_ 7.5, 3H, N-CH_2_-CH_2_-CH_3_); ^13^C NMR (150.93 MHz, D_2_O) *δ* = 48.92 (s, N-CH_2_-CH_2_-CH_3_), 43.28 (d, ^2^*J*_C,P_ 3.5, CH_2_-CH_2_-P), 25.00 (d, ^1^*J*_C,P_ 131.5, CH_2_-P), 19.16 (s, CH_2_-CH_3_), 10.09 (s, CH_3_); ^31^P NMR (162.02 MHz, D_2_O) *δ* = 18.26 (s); HRMS calculated for [C_5_H_14_NO_3_P+Na]^+^: 190.0604, found: 190.0601; anal. calc. for C_5_H_14_NO_3_P: C 35.93%, H 8.44%, N 8.38%, O 28.72%, P 18.53%, found: C 35.62%, H 8.61%, N 8.42%, O 29.09%, P 17.30%; IR (ATR) *v* = 2967, 2761, 2599, 2460, 2325, 1710, 1496, 1146, 1014 cm^-1^.

#### Synthesis of Di- and rimethylated AEP derivatives

##### Diethyl 2-*N,N*-dimethylamino-ethylphosphonate (8, partially as hydrobromide)

Related to Scheme S2. **8** was obtained from **1** and dimethyl amine (12 eq.) following **procedure A**. ^1^H NMR (700.40 MHz, CDCl_3_) *δ* = 4.13-4.04 (m, 4H, 2 × O-CH_2_) 2.73-2.65 (m, 2H, CH_2_-N), 2.32 (s, 6H, 2 × N-CH_3_), 2.04-1.97 (m, 2H, CH_2_-P), 1.30 (t, ^3^*J*_H,H_ 7.1, 6H, 2 × O-CH_2_-CH_3_); ^13^C NMR (176.12 MHz, CDCl_3_) *δ* = 61.70 (d, ^2^*J*_C,P_ 6.4, 2C, 2 × O-CH_2_), 52.57 (s, CH_2_-N), 44.44 (s, 2C, 2 × NCH_3_) 23.86 (d, ^1^*J*_C,P_ 139.3, C-P), 16.39 (d, ^3^*J*_C,P_ 6.0 Hz, 2C, 2 × O-CH_2_-CH_3_); ^31^P NMR (162.03 MHz, CDCl_3_) *δ* = 29.45 (s); HRMS calculated for [C_8_H_20_NO_3_P+H]^+^: 210.1259, found: 210.1250; IR (ATR) *v* = 3471, 2979, 2820, 2768, 1234, 1022, 952 cm^-1^.

##### 2-*N,N*-Dimethylamino-ethylphosphonic acid (M_2_AEP, 9, 71% over two steps)

Related to Scheme S2. **9** was obtained from **8** following **procedure B**. m.p. 144-147°C; ^1^H NMR (600.25 MHz, D_2_O) *δ* = 3.38-3.31 (m, 2H, N-CH_2_), 2.92 (s, 6H, 2 × CH_3_), 2.09-1.99 (m, 2H, P-CH_2_); ^13^C NMR (150.93 MHz, D_2_O) *δ* = 53.73 (s, N-CH_2_), 42.38 (s, 2C, 2 × CH_3_), 23.72 (d, ^1^*J*_C,P_ 130.2, CH_2_-P); ^31^P NMR (162.02 MHz, D_2_O) *δ* = 17.64 (s); HRMS calculated for the dimer [C_8_H_24_N_2_O_6_P_2_+H]^+^: 307.1182, found: 307.1182; anal. calc. for C_4_H_12_NO_3_P × 0.25 H_2_O: C 35.93%, H 8.44%, N 8.38%, O 28.72%, P 18.53%, found: C 35.62%, H 8.61%, N 8.42%, O 29.09%, P 17.30%; IR (ATR) *v* = 3224, 2642, 2360, 1499, 1148, 1042, 903, 473 cm^-1^.

##### Diethyl 2-*N,N,N*-trimethylammonium-ethylphosphonate iodide (**10**)

Related to Scheme S2. **8** was dissolved in ethyl acetate (10 mL), washed with an aqueous solution of sodium hydroxide (20%, 3 × 5 mL), the organic phase was dried (MgSO_4_), and concentrated *in vacuo*. The resulting residue was used for the synthesis of diethyl 2-(*N,N,N*-.trimethylammonium)-ethylphosphonate iodide (**10**; 330 mg, 0.94 mmol, 84%) following a literature procedure^55^ m.p. 155-156°C; ^1^H NMR (700.40 MHz, D_2_O) *δ* = 4.26-4.17 (m, 4H, 2 × O-CH_2_) 3.65-3.59 (m, 2H, CH_2_-N), 3.17 (s, 9H, 3 × N-CH_3_), 2.58-2.49 (m, 2H, CH_2_-P), 1.35 (t, ^3^*J*_H,H_ 7.1, 6H, 2 × O-CH_2_-CH_3_); ^13^C NMR (176.12 MHz, D_2_O) *δ* = 64.13 (d, ^2^*J*_C,P_ 6.7, 2C, 2 × O-CH_2_), 60.22 (d, ^2^*J*_C,P_ 2.6, CH_2_-N), 52.57 (t, ^1^*J*_C,N_ 3.4, 3C, 3 × N-CH_3_. Note that ^14^N has a spin of 1 and it exists in three spin states +1, 0, -1. Thus, coupling to ^14^N nuclei can lead to triplets. This coupling is rarely observed in nuclei with spin ½. Here it is observed because of the symmetric electron distribution in the ammonium group, giving no electric field gradient around the nucleus. 19.67 (d, ^1^*J*_C,P_ 139.7, C-P), 15.52 (d, ^3^*J*_C,P_ 5.8, 2C, 2 × O-CH_2_-CH_3_); ^31^P NMR (161.98 MHz, D_2_O) *δ* = 27.24 (t, ^3^*J*_P,N_ 7.0); HRMS calculated for [C_9_H_23_INO_3_P-I]^+^: 224.1410, found: 224.1410; anal. calc. for C_9_H_23_INO_3_P × 0.1 H_2_O: C 30.63%, H 6.63%, N 3.97%, O 14.05%, P 8.78%, found: C 30.47%, H 6.64%, N 4.16%, O 13.82%, P 8.44%; IR (ATR) *v* = 2980, 1739, 1480, 1240, 1020, 969, 821, 558 cm^-1^.

##### 2-*N,N,N*-Trimethylammonio-ethylphosphonic acid (M_3_AEP, 11)

Related to Scheme S2. Diethyl 2-*N,N,N*-trimethylammonium-ethylphosphonate iodide (**10**, 330 mg, 0.94 mmol, 1 eq.) was dissolved in H_2_O (1 mL), conc. HCl (0.8 mL, 9.4 mmol, 10 eq.) was added and the mixture was stirred at 110°C for 12 h. The solvent was removed under reduced pressure and the crude product was purified by ion exchange chromatography (Dowex Monosphere-550A / OH^–^-form). For this purpose, the product was applied to the ion exchange resin, then the column was washed with H_2_O (3 column volumes), then the product was eluted with an aqueous HCOOH solution (5% v/v). Finally the solvent was removed *in vacuo* to give 2-*N,N,N*-trimethylammonio-ethylphosphonic acid (**11**; 177 mg, 0.79 mmol, 84%). The solid residue can be crystallized from EtOH to give colorless crystals. m.p. 248-250°C; ^1^H NMR (600.25 MHz, D_2_O) *δ* = 3.55-3.50 (m, 2H, CH_2_-N), 3.15 (s, 9H, 3 × N-CH_3_), 2.17-2.09 (m, 2H, CH_2_-P); ^13^C NMR (150.93 MHz, D_2_O) *δ* = 62.77 (d, ^2^*J*_C,P_ 2.8, CH_2_-N), 52.50 (t, ^1^*J*_C,N_ 4.1, 3C, 3 × N-CH_3_) 22.73 (d, ^1^*J*_C,P_ 127.7, C-P); ^31^P NMR (162.02 MHz, D_2_O) *δ* = 16.83 (t, ^3^*J*_P,N_ 6.7); HRMS calculated for the dimer [C_10_H_28_N_2_O_6_P_2_+H]^+^: 335.1495, found: 335.1493; anal. calc. for C_5_H_14_NO_3_P × H_2_O: C 32.43%, H 8.71%, N 7.56%, O 34.56%, P 16.73%, found: C 32.15%, H 8.79%, N 7.65%, O 34.68%, P 16.08%; IR (ATR) *v* = 2980, 1739, 1480, 1240, 1020, 969, 821, 558 cm^-1^.

#### Synthesis of Di- and Trimethylated derivatives of *(R)-*HAEP

##### (*R*)-1-Hydroxy-2-(*N,N*-dimethylammonio)-ethylphosphonic acid [(*R*)-18, (*R*)-M_2_HAEP]

Related to Scheme S3. (*R*)-1-Hydroxy-2-aminoethylphosphonic acid^18^ [(*R*)-**13**; 141 mg, 1.00 mmol, 1 eq.] was dissolved in formic acid (conc., 0.38 mL, 10.00 mmol, 10 eq.), formaldehyde (37% in H_2_O, 0.60 mL, 6 mmol, 6 eq.) was added and the mixture was refluxed for 18 h. The solvent was removed under reduced pressure and the crude residue was purified by ion exchange chromatography (Dowex 50W × 8 / H^+^-form) with H_2_O as eluent. The product containing fractions (ninhydrin positive) were pooled and the solvent was removed *in vacuo* to yield (*R*)- 1-hydroxy-2-(dimethylammonio)-ethylphosphonic acid [(*R*)-**18**, 86 mg, 0.51 mmol, 51%] as colorless solid which can be recrystallized from H_2_O/EtOH. m.p. 257°C (decomp.); ^1^H NMR (600.25 MHz, D_2_O) *δ* = 4.16-4.08 (m, 1H, CH), 3.44-3.35 (m, 2H, CH_2_), 3.00 (s, 3H, CH_3_), 3.00 (s, 3H, CH_3_); ^13^C NMR (150.93 MHz, D_2_O) *δ* = 63.10 (d, ^1^*J*_C,P_ 153.6, CH), 59.06 (d, ^2^*J*_C,P_ 10.9, CH_2_), 44.60 (s, CH_3_), 41.01 (s, CH_3_); ^31^P NMR (162.02 MHz, D_2_O) *δ* = 14.31 (s); HRMS calculated for the dimer [C_8_H_24_N_2_O_8_P_2_+H]^+^: 339.1081, found: 339.1080; anal. calc. for C_4_H_12_NO_4_P × H_2_O: C 25.67%, H 7.54%, N 7.49%, O 42.75%, P 16.55%, found: C 25.51%, H 7.70%, N 7.40%, O 42.35%, P 16.03%; IR (ATR) *v* = 3165, 3006, 2619, 1458, 1085, 922, 560, 540 cm^-1^; $[{\alpha ]}_{D}^{20}$ = –48.3 (*c* 1.09, H_2_O).

##### (*R*)-1-Hydroxy-2-(*N,N,N*-trimethylammonio)-ethylphosphonic acid [(*R*)-**19**, (*R*)-M_3_HAEP]

Related to Scheme S3. (*R*)-**13** (479 mg, 3.40 mmol, 1 eq.) was dissolved in MeOH (45 mL) and K_2_CO_3_ (2.347 g, 16.98 mmol, 5 eq.) was added. After 10 minutes, methyl iodide (1.928 g, 13.58 mmol, 0.85 mL, 4 eq.) was added and stirring was continued for 48 h. The solvent was removed *in vacuo* and the residue was dissolved in H_2_O (3.4 mL). Subsequently, the pH was adjusted to 1 with conc. H_2_SO_4_ and the resulting mixture was stirred for 1 h. The solvent was removed under reduced pressure, the residue was dissolved in water and washed Et_2_O (3 × 4 mL). The aqueous phase was concentrated to dryness, dissolved in a minimal amount of water and applied to an ion exchange chromatography resin (Dowex Monosphere-550A / OH^–^-form). An aqueous solution of formic acid (5% v/v) was used as eluent. The product containing fractions were pooled and concentrated. The obtained amorphous solid can be recrystallized from EtOH/H_2_O to give (*R*)-1-hydroxy-2-(*N,N,N*-trimethylammonio)-ethylphosphonic acid [(*R*)-**19**, 501 mg, 2.72 mmol, 80%] as colorless needles. m.p. 269-272°C; ^1^H NMR (600.25 MHz, D_2_O): *δ* = 4.34 (ddd, ^2^*J*_H,P_ 11.4, ^3^*J*_H,H_ 10.1, ^3^*J*_H,H_ 1.3, 1H, CH-P), 3.65 (ddd, ^2^*J*_H,H_ 14.2, ^3^*J*_H,P_ 4.6, ^3^*J*_H,H_ 1.3, 1H, CH_2_), 3.58 (ddd, ^2^*J*_H,H_ 14.2, ^3^*J*_H,H_ 10.1, ^3^*J*_H,P_ 3.0, 1H, CH_2_), 3.24 (s, 9H, 3 × CH_3_); ^13^C NMR (150.93 MHz, D_2_O): *δ* = 68.06 (dt^i^, ^2^*J*_C,P_ 12.7, ^1^*J*_C,N_ 2.3, CH_2_), 64.17 (d, ^1^*J*_C,P_ 151.7, CH), 53.97 (s, CH_3_), 53.95 (s, CH_3_), 53.92 (s, CH_3_) ppm; ^31^P NMR (161.98 MHz, D_2_O): *δ* = 14.05 (t^i^, ^3^*J*_P,N_ 6.2); HRMS calculated for [C_5_H_14_NO_4_P+H]^+^: 184.0739, found: 184.0730; anal. calc. for C_5_H_14_NO_4_P × 2.5 H_2_O: C 26.32%, H 8.39%, N 6.14%, O 45.57%, P 13.57%, found: C 26.37%, H 8.45%, N 6.06%, O 45.20%, P 13.44%; IR (ATR): *v* = 3160, 2337, 1599, 1478, 1248, 1084, 967, 914 cm^-1^; $[{\alpha ]}_{D}^{20}$ = –29.0 (*c* 0.91, D_2_O).

#### Synthesis of a Monomethylated Derivative of *(R)-*HAEP

##### (*R*)-Diisopropyl 1-hydroxy-2-aminoethylphosphonate [(*R*)-**14**]

Related to Scheme S4. Hydrazine-monohydrate (4.06 mmol, 0.2 mL, 2 eq.) was added to a solution of (*R*)-diisopropyl (2-(1,3-dioxoisoindolin-2-yl)-1-hydroxyethyl)phosphonate^5^ [(*R*)-**12**, 2.03 mmol, 721 mg, 1eq.] in ethanol (10 mL) and the mixture was stirred at 80°C for 2 h. The reaction mixture was filtered, concentrated *in vacuo* and the resulting residue was purified by column chromatography (Al_2_O_3_; EtOH; Note that the compound could not be visualized using the ninhydrin stain described in the **general experimental section** in combination with Al_2_O_3_-coated TLC plates. Thus, SiO_2_-coated TLC plates were used to identify the product-containing fractions) giving (*R*)-diisopropyl 2-amino-1-hydroxyethyl-phosphonate [(*R*)-**14**, 349 mg, 1.6 mmol, 80%] as a colorless oil. ^1^H NMR (600.25 MHz, CD_3_OD) *δ* = 4.78-4.69 (m, 2H, 2 × O-CH), 3.80 (ddd, ^3^*J*_H,H_ 9.1, ^2^*J*_C,P_ 7.5, ^3^*J*_H,H_ 3.6, 1H, CH-P), 2.95 (ddd, ^2^*J*_H,H_ 13.5, ^3^*J*_C,P_ 7.6, ^3^*J*_H,H_ 3.6, 1H, CH_2_), 2.84 (ddd, ^2^*J*_H,H_ 13.5, ^3^*J*_H,H_ 9.1, ^3^*J*_C,P_ 7.8, 1H, CH_2_); 1.36-1.33 (m, 12H, 4 × CH_3_); ^13^C NMR (150.93 MHz, CD_3_OD) *δ* = 73.15 (d, ^2^*J*_C,P_ 7.5, CH-O), 72.98 (d, ^2^*J*_C,P_ 7.3, CH-O), 69.74 (d, ^1^*J*_C,P_ 164.8, CH-P), 43.79 (d, ^2^*J*_C,P_ 6.2, CH_2_), 24.38 (d, ^3^*J*_C,P_ 2.5, CH_3_), 24.36 (d, ^3^*J*_C,P_ 2.5, CH_3_), 24.23 (d, ^3^*J*_C,P_ 4.7, 2C, 2 × CH_3_); ^31^P NMR (162.02 MHz, CD_3_OD) *δ* = 21.84 (s); HRMS calculated for [C_8_H_20_NO_4_P+H]^+^: 226.1208, found: 226.1203; IR (ATR) *v* = 3286, 2979, 2934, 1467, 1375, 1212, 975, 567 cm^-1^.

##### (*R*)-Diisopropyl 1-hydroxy-2-tosylamido-ethylphosphonate [(*R*)-**15**]

Related to Scheme S4. (*R*)-**14** (0.67 mmol, 152 mg, 1 eq.) was dissolved in dry THF (5 mL), then triethylamine (2.7 mmol, 0.38 mL, 4 eq.) and tosyl chloride (0.74 mmol, 141 mg, 1.1 eq.) were added dropwise in a row at 0°C. After stirring for 20 h at room temperature, CH_2_Cl_2_ (5 mL) was added and the mixture was washed with brine. The layers were separated and the aqueous phase was extracted with CH_2_Cl_2_ (3 × 5 mL). The combined organic layers were dried (MgSO_4_), filtered and concentrated *in vacuo*. The crude product was purified by MPLC [R_f_ (*n*-heptane:EtOAc 1:2) = 0.69; *n*-heptane/EtOAc, 16 → 100 % EtOAc], to give (*R*)-diisopropyl 1-hydroxy-2-(tosylamido)-ethylphosphonate [(*R*)-**17**; 214 mg, 0.56 mmol, 84%] as a colorless, crystalline solid; m.p. 118-119°C. ^1^H NMR (600.18 MHz, CDCl_3_) *δ* = 7.75 (d, ^3^*J*_H,H_ 8.2, 2H, 2 × CH_ar_), 7.31 (d, ^3^*J*_H,H_ 8.2, 2H, 2 × CH_ar_), 5.43 (dd, *J* 7.9, 4.0, 1H, NH) 4.75-4.66 (m, 2H, 2 × O-CH) 3.85 (qd, ^3^*J*_H,H_; ^2^*J*_H,P_ 7.1, ^3^*J*_H,H_ 4.0, 1H, CH-P), 3.71 (t, *J* 6.0, 1H, OH), 3.35 (tdd, ^2^*J*_H,H_ 12.1, ^3^*J*_H,P_ 8.2, ^3^*J*_H,H_ 4.0, 1H, CH_2_), 3,14 (dtd, ^2^*J*_H,H_ 12.1, ^3^*J*_H,H_ 7.1, ^3^*J*_H,P_ 4.2, 1H, CH_2_), 2.42 (s, 3H, Ph-CH_3_), 1.62 (s, 1H, OH), 1.31 (d, ^3^*J*_H,H_ 5.9, 3H, CH_3_), 1.31 (d, ^3^*J*_H,H_ 5.6, 3H, CH_3_) , 1.30 (d, ^3^*J*_H,H_ 6.0, 3H, CH_3_) , 1.26 (d, ^3^*J*_H,H_ 6.2, 3H, CH_3_); ^13^C NMR (150.93 MHz, CDCl_3_) *δ* = 143.57 (s, S-C_ar._), 136.73 (s, C_ar._-CH_3_), 129.76 (s, 2C, 2 × CH_ar._), 127.15 (s, 2C, 2 × CH_ar._), 72.10 (d, ^2^*J*_C,P_ 7.0, CH-O), 72.06 (d, ^2^*J*_C,P_ 6.1, CH-O), 66.71 (d, ^1^*J*_C,P_ 162.5 Hz, CH-P), 44.47 (d, ^2^*J*_C,P_ 5,3 Hz, CH_2_) 24.07 (d, ^3^*J*_C,P_ 2.9 Hz, 2C, 2 × CH_3_), 23.88 (d, ^3^*J*_C,P_ 5.0 Hz, CH_3_), 23.81 (d, ^3^*J*_C,P_ 4.6 Hz, CH_3_), 21.5 (s, Ph-CH_3_); ^31^P NMR (162.02 MHz, CDCl_3_) *δ* = 19.75 (s); HRMS calculated for [C_15_H_26_NO_6_PS+Na]^+^: 402,1116, found: 402,1108; anal. calc. for C_15_H_26_NO_6_PS: C 47.49%, H 6.91%, N 8.45%, O 25.30%, P 8.16%, S 3.69%, found: C 47.38%, H 6.87%, N 8.28%, P 7.95%, S 3.68%; IR (ATR) *v* = 3470, 3433, 3092, 2983, 2904, 1738, 981 cm^-1^; $[{\alpha ]}_{D}^{20}$ = –26.0 (*c* 0.60, acetone).

##### (*R*)-Diisopropyl 1-hydroxy-2-(*N*-methyl-*N*-tosyl-amido)-ethylphosphonate [(*R*)-**16**]

Related to Scheme S4. First, potassium carbonate (0.53 mmol, 73 mg, 2 eq.), then methyl iodide (0.79 mmol, 50 μl, 3 eq.) was added to a solution of (*R*)-**15** (0.26 mmol, 100 mg, 1 eq.) in dry DMF (1 mL). After stirring at room temperature for 18 h, the reaction mixture was quenched by addition of H_2_O (2mL). The organic phase was separated, and the aqueous phase was extracted with Et_2_O (3 × 3 mL). The combined organic layers were dried (MgSO_4_), filtered and concentrated *in vacuo*. The residue was purified by MPLC [*R*_f_ (*n*-heptane:EtOAc 1:4) = 0.34; *n*-heptane/EtOAc, 20 → 100 % EtOAc], giving (*R*)-diisopropyl 1-hydroxy-2-(*N*-methyl-*N*-tosyl-amido)-ethylphosphonate [(*R*)-**16**, 85 mg, 0.22 mmol, 83%] as a colorless solid; m.p. 110-111°C; ^1^H NMR (600.18 MHz, CDCl_3_) *δ* = 7.68 (d, ^3^*J*_H,H_ 8.1, 2H, 2 × CH_ar._), 7.33 (d, ^3^*J*_H,H_ 8.1, 2H, 2 × CH_ar._), 4.77 (dhept, ^3^*J*_H,P_ 12.4, ^3^*J*_H,H_ 6.2, 2H, 2 × O-CH) 4.06 (ddd, ^3^*J*_H,H_ 9.3, ^2^*J*_H,P_ 7.8, ^3^*J*_H,H_ 3.1 Hz, 1H, CH-P), 3.38-3.27 (m, 2H, CH_2_), 2.88 (s, 3H, N-CH_3_), 2.79 (brs, 1H, OH), 2.44 (s, 3H, Ph-CH_3_), 1.37-1.34 (m, 12H, 4 × CH-CH_3_); ^13^C NMR (150.92 MHz, CDCl_3_) *δ* = 143.71 (s, C_ar._-S), 134.45 (s, C_ar._-CH_3_), 129.82 (s, 2C, 2 × CH_ar._), 127.43 (s, 2C, 2 × CH_ar._), 71.95 (d, ^2^*J*_C,P_ 7.5, O-CH), 71.74 (d, ^2^*J*_C,P_ 6.7, O-CH), 67.25 (d, ^1^*J*_C,P_ 162.1, CH-P), 52.00 (d, ^2^*J*_C,P_ 8.9, CH_2_), 36.73 (s, N-CH_3_), 24.13 (d, ^3^*J*_C,P_ 3.4, 2C, 2 × CH_3_), 24.00 (d, ^3^*J*_C,P_ 4.1, CH_3_), 23.97 (d, ^3^*J*_C,P_ 4.1, CH_3_), 21.54 (s, Ph-CH_3_); ^31^P NMR (162.02 MHz, CDCl_3_) *δ* = 19.34 (s); IR (ATR) v = 3267, 2985, 2922, 2853, 1278, 945, 525 cm^-1^; $[{\alpha ]}_{D}^{20}$ = –25.2 (*c* 1.04, acetone). Ee-determination was accomplished by ^31^P NMR spectroscopy in the presence of a chiral solvating agent ((+)-(*R*)-*tert*-butylphenylphosphinothioic acid)^81^^,^^82^ (161.98 MHz, CDCl_3_): *δ* = 97.17 (∫ 0.7082, chiral solvating agent), 19.49 [∫ 0.2903, complex of chiral solvating agent with (*R*)- **16**], 19.67 [∫ 0.0015, complex of chiral solvating agent with (*S*)- **16**] ≙ ee ≥ 99%.

##### (*R*)-1-Hydroxy-2-(*N*-methylammonio)ethylphosphonic acid [(*R*)-M_1_HAEP; (*R*)-**17**]

Related to Scheme S4. (*R*)-**16** (172 mg, 0.44 mmol) was allowed to react in a manner similar to that described in **general procedure B** to give (*R*)-1-hydroxy-2-(methylammonio)ethylphosphonic acid [(*R*)-**17**, 56 mg, 23 mmol, 83%]. m.p.. 261-264°C; ^1^H NMR (600.25 MHz, D_2_O) *δ* = 4.02 (td, ^3^*J*_H,H_; ^2^*J*_H,P_ 10.5, ^3^*J*_H,H_ 3.1, 1H, CH), 3.36 (ddd, ^2^*J*_H,H_ 13.1, ^3^*J*_H,P_ 4.8, ^3^*J*_H,H_ 3.1, 2H, CH_2_), 3.24 (ddd, ^2^*J*_H,H_ 13.1, ^3^*J*_H,H_ 10.5, ^3^*J*_H,P_ 6.1, CH_2_), 2.79 (s, 3H, CH_3_); ^13^C NMR (150.93 MHz, D_2_O) *δ* = 64.39 (d, ^1^*J*_C,P_ 155.0, CH), 50.72 (d, ^2^*J*_C,P_ 9.3, CH_2_), 32.77 (s, CH_3_); ^31^P NMR (162.02 MHz, D_2_O) *δ* = 14.59 (s); HRMS calculated for the dimer [C_6_H_21_N_2_O_8_P_2_+H]^+^: 311.0768, found: 311.0766; anal. calc. for C_3_H_10_NO_4_P: C 23.23%, H 6.50%, N 9.03%, O 41.26%, P 19.97%, found: C 23.02%, H 6.52%, N 8.82%, O 41.20%, P 20.05%; IR (ATR) *v* = 3194, 2994, 2702, 2521, 2460, 1632, 956, 482 cm^-1^; $[{\alpha ]}_{D}^{20}$ = –38.3 (*c* 0.84, H_2_O).

#### Bioinformatics analyses

An initial visual inspection of bacterial AEP degradation gene clusters was performed using tools publicly available at the Department of Energy Integrated Microbial Genomes (IMG) database^75^ and at the MicrobesOnline website.^74^ This preliminary analysis revealed the frequent presence of genes encoding putative FAD-dependent oxidoreductases in clusters for the hydrolytic degradation of AEP. The FAD enzymes belonged to at least three subgroups, exemplified by the representative sequences from *Vibrio vulnificus* (PbfB, GenBank: WP_049798008), *Azospirillum sp. B510* (PbfC, GenBank: WP_012976454) and *Acinetobacter baumannii* (PbfD, GenBank: WP_079548425).

Homologs of the three oxidoreductases were identified using a BLASTp search^76^ against complete bacterial genomes available at the IMG. Only sequences that shared significant similarity to PbfB, PbfC or PbfD and whose genes were found within the expected genomic context (*i.e*. AEP degradation gene clusters) were considered for further analysis.

The phylogenetic relationships among PbfB, PbfC and PbfD, as well as between these enzymes and other functionally characterized oxidoreductases sharing a PF01266 domain, were investigated as follows.

Based on genomic searches, we assembled an initial set encompassing all the sequences containing a PF01266 domain that we identified within AEP degradation gene clusters (260 sequences). This initial set was reduced to 64 representative proteins, primarily by removing all sequences showing a pairwise identity higher than 90% with CD-HIT.^83^ The list of these 64 representative sequences (dataset A) is provided in Table S1. These sequences were aligned with MUSCLE^77^ and the resulting multiple sequence alignment (MSA) was trimmed according to the boundaries of the PF01266 domain. Alignment positions that were poorly informative in terms of phylogeny, (e.g., sites where >50% of sequences in the alignment showed gaps) were removed with Gblocks.^84^ Following this curation step, the MSA was used as an input for a Maximum Likelihood analysis with IQ-TREE v.1.6.12.^78^ Radial unrooted trees were graphically depicted with the FigTree software.

A second, larger dataset (dataset B) comprising a total of 80 sequences was subsequently prepared by adding a custom selection of oxidoreductases of known function, which shared with PbfA, PbfB and PbfC the presence of the PF01266 domain. In addition to the 64 sequences from dataset A, dataset B contained: (i) two monomeric sarcosine oxidases (SoxA); (ii) two D-amino acid dehydrogenases (DadA); (iii) two γ-*N*-methyl aminobutyrate oxidases (MabO); (iv) two glycine oxidases (ThiO); (v) two γ-glutamylputrescine oxidases (PuuB); (vi) two *N*-methyl-L-tryptophan oxidases (SolA); (vii) two mitochondrial sarcosine dehydrogenases (SarDH); (viii) two mitochondrial dimethylglycine dehydrogenases (DMGDH). The accession IDs of these additional sequences are provided in Table S2. Dataset B was processed as described for dataset A, removing additional C-terminal domains prior to the preparation of the MSA.

ModelFinder^79^ identified the WAG+F+R5 and LG+R5 molecular models of evolution as the most appropriate ones for dataset A and B, respectively, based on the Bayesian Information Criterion. The reliability of the two phylogenetic trees, with particular reference to the monophyly of the PbfA, PbfB and PbfC clades, was tested with 1000 ultrafast bootstrap replicates.^85^

#### Plasmid constructs

The synthetic codon-optimized genes of the FAD-dependent enzymes PbfB (GenBank: WP_049798008), PbfC (GenBank: WP_012976454), PbfD1 (GenBank: WP_079548425) and PbfD2 (GenBank: WP_075082418) were purchased from Proteogenix (Schiltigheim, France) or from BaseGene BV (Leiden, the Netherlands) and cloned into the NdeI/NotI restriction sites of a pET28a-N-His-tag vector.

#### Expression and purification of recombinant PbfC

Related to Figure S9. PbfC from *Azospirillum* was overexpressed in *E. coli* Tuner (DE3) cells. A 10-mL bacterial preculture grown overnight at 37°C was used to inoculate 1L of Luria-Bertani broth (LB) containing kanamycin (50 μg/mL) until OD_600_ reached 0.7-0.8. The temperature was then lowered to 20°C and protein expression was induced by adding 0.25 mM isopropyl-β-D-1-thiogalactopyranoside (IPTG). 20 hours after induction, the cells were harvested by centrifugation. The cell pellets were washed once with phosphate-buffered saline solution (PBS), resuspended, centrifuged again, and finally stored at −20°C.

For protein purification, pellets were resuspended in **buffer A** (50 mM sodium pyrophosphate pH 8.5, 150 mM NaCl), supplemented with 1 mM phenylmethylsulfonyl fluoride (PMSF), 1 mM benzamidine, 10% glycerol and 10 μM FAD. The cell suspension was sonicated and centrifuged (26,200 × g for 40 minutes at 4°C).

After sonication, the cell lysate was cleared by centrifugation (26,200 × g for 40 minutes at 4°C). The supernatant was recovered and continuously looped (flow rate: 1 mL/min) on a 5 mL HisTrap™ Fast Flow column (Cytiva) for 2 hours at 4°C through a peristaltic pump. Afterwards, the loaded column was mounted onto an AKTA Pure System FPLC apparatus and proteins were eluted with a gradient of buffer A supplemented with 208 mM imidazole. Fractions containing PbfC were pooled and extensively dialyzed against buffer A, supplemented with 10% glycerol and 10 μM FAD, then concentrated by an Amicon® Ultra-2 3K device (Merck) to 3 mL. The protein was further loaded into a 5 mL loop of AKTA Pure System FPLC and purified by size exclusion chromatography (SEC) with a HiPrep™ 16/60 Sephacryl S-300 HR (Cytiva) column, equilibrated with the same buffer as above (buffer A plus 10% glycerol and 10 μM FAD). The protein was finally concentrated by Amicon centrifugation and subdivided into aliquots (about 0.5 mL each) to be stored at −80°C. Protein purity was assessed higher than 95% by SDS-PAGE and the final yield was ≈3 mg of purified PbfC per liter of bacterial culture. A SDS-PAGE of purified PbfC (side by side with purified PbfD1 and PbfD2) is shown in Figure S9.

#### Expression and purification of recombinant PbfD1

Related to Figure S9. PbfD1 from *Acinetobacter baumannii* was expressed in *E. coli* Tuner (DE3) cells (EMD Biosciences), which were grown in self-inducing medium (LB containing glucose 0.5 g/L and lactose 2 g/L). A 10-mL bacterial preculture grown overnight at 37°C was used to inoculate 1L of self-inducing medium containing kanamycin (50 μg/mL). The culture was kept under agitation at 20°C for up to 24 h, then the cells were harvested by centrifuging at 7,200 × g for 10 min at 4°C.

The pellet was resuspended in **buffer B** (50 mM sodium phosphate pH 7.5, 200 mM NaCl) and centrifuged again. The supernatant was discarded, while the pellets were stored at −20°C.

For protein purification, the pelleted cells were thawed on ice and resuspended in buffer B supplemented with 1 mM PMSF, 1 mM benzamidine, 10% glycerol and 10 μM FAD. Lysozyme (1 mg/mL final) was added to the cell suspension, which was then incubated at 4°C under agitation for 30 minutes and finally sonicated. At the end of sonication, the lysate was centrifuged at 26,200 × g for 40 minutes (at 4°C) and the supernatant was used for the next purification step.

PbfD1 purification exploited a 5 mL HisTrap™ Fast Flow column (Cytiva). The cleared lysate was continuously looped (flow rate: 2 mL/min) on the column for 2 hours at 4°C, after which the column was transferred to an AKTA Pure System FPLC apparatus.

The protein was eluted with a gradient of buffer B supplemented with 250 mM imidazole. PbfD1 fractions were pooled and extensively dialyzed against a storage buffer (50 mM TEA-HCl pH 7.5, 200 mM NaCl, 10% glycerol). The protein was subdivided into aliquots (about 0.5 mL each) to be stored at −80°C. Protein purity was assessed higher than 95% by SDS-PAGE and the final yield was ≈100 mg of purified PbfD1 per liter of bacterial culture.

#### Expression and purification of recombinant PbfD2

Related to Figure S9. Production of PbfD2 in a partially soluble form was achieved by expressing the protein in *E. coli* Chaperone Competent pGro7/BL21 cells (Takara) and exploiting D-sorbitol as a carbon source in the growth medium. This reportedly promotes the activation of an osmotic stress response helpful to increase protein solubility.^86^

A 10-mL bacterial preculture grown at 37°C overnight was used to inoculate 1 L of self-inducing medium (LB containing glucose 0.5 g/L and lactose 2 g/L) supplemented with kanamycin (50 μg/mL), chloramphenicol (20 μg/mL), arabinose (0.5 mg/mL, to allow induction of chaperonins by the pGro7/BL21 cells) and D-sorbitol (5 mg/mL). The culture was kept under agitation at 20°C for up to 24 h, then the cells were harvested by centrifugation at 7,200 × g for 10 min (4°C). After removing the supernatant, the pellet was resuspended in buffer B, then centrifuged again, discarding the supernatant. The pellets were then stored at −20°C waiting to proceed with lysis and purification.

For protein purification, pellets were thawed and resuspended in buffer B, supplemented with 1 mM PMSF, 1 mM benzamidine, 10% glycerol, 0.05% Triton® X-100 and 10 μM FAD. After adding lysozyme 1 mg/mL, the cell suspension was incubated in a cold room under agitation for 30 minutes.

The suspension was frozen in liquid N_2_ and thawed quickly at 37°C in a thermostated water bath. The freezing-thawing procedure was repeated two more times, after which the suspension was sonicated briefly. At the end of sonication, the lysate was centrifuged at the maximum speed (26,200 × g for 40 minutes at 4°C); the pellet was discarded and the supernatant was retained for further processing.

PbfD2 purification exploited also in this case a 5 mL HisTrap™ Fast Flow column (Cytiva). The lysate was continuously looped (flow rate: 2 mL/min) on the column for 2 hours at 4°C, after which the column was transferred to the FPLC apparatus.

The column was first washed with 30 mL of chaperone-removal buffer (50 mM Na phosphate pH 7.5, 100 mM KCl, 10% glycerol, 500 mM sucrose, 20 mM MgCl_2_, 5 mM ATP) to displace the chaperonins bound to nickel resin; subsequently, PbfD2 was eluted with a gradient of buffer B supplemented with 250 mM imidazole. PbfD2 fractions were pooled and concentrated by an Amicon® Ultra-2 3K device (Merck), then extensively dialyzed against a buffer containing 50 mM TEA pH 7.5, 200 mM NaCl and 10% glycerol. The protein was subdivided into aliquots (about 0.5 mL each) to be stored at −80°C. Protein purity was assessed higher than 80% by SDS-PAGE and the final yield was ≈2.5 mg of purified PbfD2 per liter of bacterial culture.

#### Absorbance measurements and establishment of extinction coefficients for PbfC, PbfD1 and PbfD2

Absorption spectra were recorded with either a Cary 50 (Varian) or with a thermostated V-750 (Jasco Inc.) UV-Vis spectrophotometer and corrected for buffer contribution.

Denaturation of the proteins with sodium dodecyl sulfate, aimed at identifying the type of cofactor (FAD or Flavin mononucleotide, FMN) and at calculating the extinction coefficients of protein-bound flavin, was carried out as described by Aliverti et al.^48^ Briefly, spectra of the enzymes were collected in TEA-HCl 50 mM pH 8.0, then SDS was added to a final concentration of 1% (w/v). The samples were monitored spectroscopically until no further changes were observed in the ∼280 and ∼450 nm regions. In all cases, the final spectra showed an absorption maximum at 450 nm, typical of FAD (rather than the 446 nm maximum of FMN). These spectra were used to calculate the concentration of released cofactor, using ε_450nm_ = 11300 M^−1^cm^−1^ as described.^48^ This information was confronted with the spectra of the enzymes before SDS treatment, allowing us to estimate the extinction coefficients of the protein-bound FAD and hence (assuming a 1:1 enzyme:cofactor ratio) of the active oxidoreductase species. The calculated extinction coefficients (at the respective λ_max_) were: ε_462nm_=10,650 M^-1^ cm^-1^ for PbfC (*Azospirillum sp. B510*); ε_447_=13,340 M^-1^ cm^-1^ for PbfD1 (*A. baumannii*) and ε_456_=11,950 M^-1^ cm^-1^ for PbfD2 (*M. fucicola*). In subsequent experiments, enzyme concentrations were calculated based on these coefficients.

#### Microtiter assays for oxidoreductase activities

The oxidoreductase activity of the recombinant enzymes was first assessed through two assays conducted in 96-well microtiter format. These colorimetric assays served to rapidly screen the ability of the enzymes to oxidize a number of potential substrates (both phosphonates and non-phosphonates), transferring the electrons to either an ‘artificial’ electron acceptor or to molecular oxygen.

In the first assay, the redox dye 2,6-dichlorophenol indophenol (DCPIP) was the final electron acceptor, whereas phenazine methylsulfate (PMS) served as an electron mediator.^43^ In this assay, the oxidoreductases (0.5 μM, final concentration) were incubated in 50 mM TEA-HCl pH 8.0, 200 μM DCPIP and 3 mM PMS, in the presence or absence of the potential substrate (5 mM; 10 mM for racemic mixtures). Reactions were conducted at room temperature in a final volume of 200 μL and started with the addition of the enzyme. The plate was photographed at regular intervals over a time of 30 min.

A single-point plate assay was used to screen for the ability of the recombinant enzymes to use molecular oxygen as the electron acceptor. The plate assay detected the release of hydrogen peroxide, which was revealed by exploiting horseradish peroxidase and the chromogenic substrate *o*-dianisidine.^87^ In each well, the enzyme under examination (0.5 μM, final concentration) was incubated in 50 mM TEA-HCl pH 8.0 with 5 mM substrate (10 mM for racemic mixtures). The reaction was started by the addition of the enzyme. After a 30-min incubation at room temperature, the reaction mixture was supplemented with horseradish peroxidase (3 U) and *o*-dianisidine (1 mM final concentration). After another 5 min, 3.5 M sulfuric acid was added to each well (to reach a final volume of 100 μL) and the plate was photographed.

#### Measurement of phosphate release

Phosphate release was also determined in a microtiter assay, by employing the BIOMOL® Green kit according to the manufacturer’s instructions. Enzymes that had been stored in phosphate or pyrophosphate buffer were dialyzed against a different buffer (50 mM TEA-HCl pH 8.0, 150 mM NaCl, 10% glycerol) before using them in these assays. Likewise, cell extracts to be subjected to this assay were obtained by lysing the *E. coli* cells in phosphate-free buffer (50 mM sodium 4-(2-hydroxyethyl)-1-piperazineethanesulfonate, pH 7.5).

A typical reaction contained 50 mM TEA-HCl buffer pH 8.0, the oxidoreductase under examination (or, in the case of PbfB, which resisted purification, the extracts from *E. coli* cells expressing the recombinant enzyme), 4 μM PhnX, 2 mM MgCl_2_ and the substrate. After a one-hour incubation at room temperature, 18 μL of the reaction mixture (or 36 μL in the case of cell extracts) was transferred into 200 μL of BIOMOL® Green reagent to block the enzyme activity. Color development was assessed after another 30 min, by measuring the absorbance at 600 nm using a Cary 50 UV-Vis spectrophotometer (Varian).

#### Detection of ammonia release from AEP oxidation

To investigate the potential release of ammonia upon the oxidative deamination of AEP (which contains a primary amino group), we utilized GDH to convert α-ketoglutarate to L-glutamate by using ammonia and oxidizing NADH, whose disappearance can be monitored spectroscopically at 340 nm.

For this assay, the oxidoreductase under examination (0.45 μM in the case of PbfD1 and PbfD2; 1.9 μM in the case of PbfC) was incubated in a solution containing 50 mM TEA-HCl buffer pH 8.0, 2 mM AEP and 1 mM α-ketoglutarate. After 30 min, the mixture was supplemented with NADH (∼200 μM final concentration) and finally with GDH (1.7 U/ml final concentration). The disappearance of NADH absorption at 340 nm was monitored by a UV-Vis Cary 50 spectrophotometer. In control experiments where either the oxidoreductase or AEP was omitted, no significant NADH oxidation was observed.

#### NMR measurements

^1^H NMR spectra were recorded with a Bruker Avance-III 400 spectrometer equipped with a CRPN2-DR-BB/1H&19F-5mm-EZ PRODIGY 400 CRYOPROBE in non-spinning mode at room temperature using a gradient-based solvent suppression pulse sequence (1D Excitation Sculpting using 180º water-selective pulses “zgesgp”) for the water signal suppression (spectral width: 6399 Hz, FID size: 32768, relaxation delay: 2 s).The reaction mixture (total volume of 500 μl) contained 30 mM sodium phosphate buffer pH 8.0, 2 mM MgCl_2_, 5 mM substrate, 0.5 μM enzyme, 10% D_2_O and 1 mM of deuterated trimethylsilyl propionic acid, used as internal chemical shift reference (δ=0.00 ppm). NMR spectra were processed and analyzed with MestReNova v12.0.4.

#### Continuous kinetic assay for monitoring oxidase reactions

The oxidation of AEP, M_1_AEP and M_2_AEP (for PbfD1 only) with oxygen as the electron acceptor was monitored by coupling the production of PAA to that of acetaldehyde catalyzed by PhnX and to the oxidation of NADH by alcohol dehydrogenase (ADH).^20^^,^^23^

The reactions were conducted at 25°C in a final volume of 120 μl, containing 50 mM TEA-HCl buffer pH 8.0, 0.25 mM NADH, 2.5 mM MgCl_2_, PhnX (4 μM) and ADH (6 U), in addition to the enzyme under examination and the substrate (AEP, M_1_AEP or M_2_AEP). The disappearance of NADH was monitored spectrophotometrically (ε_340 nm_= 6,220 M^-1^cm^-1^) on a Cary 50 UV-Vis spectrophotometer.

To obtain the kinetic parameters of the oxidases, reactions at different substrate concentrations were repeated in triplicate and the data were nonlinearly least-squares fitted to the Michaelis-Menten equation using Sigma Plot.

#### Continuous spectrophotometric assay for monitoring the PbfC-catalyzed reaction

Since PbfC was very inefficient at utilizing molecular oxygen as the electron acceptor, the oxidative deamination of AEP and M_1_AEP catalyzed by PbfC was typically measured through a continuous kinetic assay based on DCPIP and PMS, similar to the assay reported by Augustin and coworkers.^43^ DCPIP and PMS were prepared and stored as described by Jahn and coworkers.^47^ Experiments to establish kinetic parameters were performed at 25°C on a thermostated V-750 UV-Vis spectrophotometer. Reaction mixtures contained 50 mM TEA-HCl pH 8.0, 80 μM DCPIP, 3 mM PMS, in addition to the aminophosphonate substrate and the enzyme. Although PbfC was able to efficiently reduce DCPIP even in the absence of the electron mediator PMS, at the relatively low DCPIP concentration used in the assay the presence of PMS was instrumental in achieving the maximal reaction rate. Reactions were started by adding the enzyme last, then the decrease in absorbance at 600 nm was followed until at least 1 min of linear reaction was observed. The extinction coefficient of DCPIP at 600 nm (18,400 M^-1^ cm^-1^) had been previously established under the same assay conditions. Each reaction was repeated in triplicate and the kinetic parameters were computed by nonlinear least-squares fitting of the data to the Michaelis-Menten equation using Sigma Plot.

### Quantification and statistical analysis

Maximum likelihood phylogenetic trees shown in Figures 2, S3, and S5 were built from MSA produced with MUSCLE, as described in the section ‘bioinformatics analyses’.

Kinetic parameters for the recombinant enzymes towards given substrates were obtained by nonlinear least-squares fitting of the experimental data (initial rates divided by enzyme concentration vs. substrate concentration) to the Michaelis-Menten equation using SigmaPlot 14.0 (Systat Software, USA). Data were collected in triplicate and the resulting kinetic parameters are reported in Table 2 ± SE of the fitting.

## Data Availability

• All data reported in this paper will be shared by the lead contact upon request. • This paper does not report original code. • Any additional information required to reanalyze the data reported in this paper is available from the lead contact upon request.

## Supplemental information

Document S1. Figures S1–S9, Schemes S1–S4, Tables S1 and S2, and Data S1
