# Supplementary material for: Deciphering the role of recurrent FAD-dependent enzymes in bacterial phosphonate catabolism
Source: iScience. 2023 Oct 4;26(11):108108. doi: 10.1016/j.isci.2023.108108 (PMC10590968; doi:10.1016/j.isci.2023.108108)
Supplement: Document S1. Figures S1–S9, Schemes S1–S4, Tables S1 and S2, and Data S1 [file mmc1.pdf]

iScience, Volume *26*

Supplemental information

Deciphering the role of recurrent FAD-dependent enzymes in bacterial phosphonate catabolism

Erika Zangelmi, Francesca Ruffolo, Tamara Dinhof, Marco Gerdol, Marco Malatesta, Jason P. Chin, Claudio Rivetti, Andrea Secchi, Katharina Pallitsch, and Alessio Peracchi

LIST OF CONTENTS

Supplemental figures

Figure S1 Gene clusters for AEP degradation containing FAD-dependent enzymes

Figure S2 Multiple sequence alignment of PbfB, PbfC and PbfD sequences.

Figure S3 Maximum likelihood phylogenetic tree of the proteins in Table S1

Figure S4 Reactions catalyzed by the enzymes in Table S2

Figure S5 Phylogenetic tree including the proteins in Tables S1 and S2

Figure S6 ^{1}H NMR analysis of the products of M_1AEP oxidation

Figure S7 ^{1}H NMR analysis of the products of M_2AEP oxidation

Figure S8 Reactions of PbfC, PbfD1 and PdfD2 towards AEP and AEP derivatives

Figure S9 SDS-PAGE of the three purified oxidoreductases used in this study

Supplemental schemes

Scheme S1 Synthetic strategy for the synthesis of compounds **5**-**7**.

Scheme S2 Synthetic strategy for the synthesis of compounds **9**-**11**

Scheme S3 Synthesis scheme for the preparation of (*R*)-**19** and (*R*)-**18**

Scheme S4 Overview of the required steps towards (*R*)-**17**

Supplemental tables

Table S1 FAD-dependent enzymes from gene clusters for AEP degradation.

Table S2 Enzymes of known function, most similar to PbfB, PbfC and PbfD.

Supplemental data

Data S1 NMR spectra of all the compounds chemically synthesized for this study

Supplemental contents references

Supplemental Figures

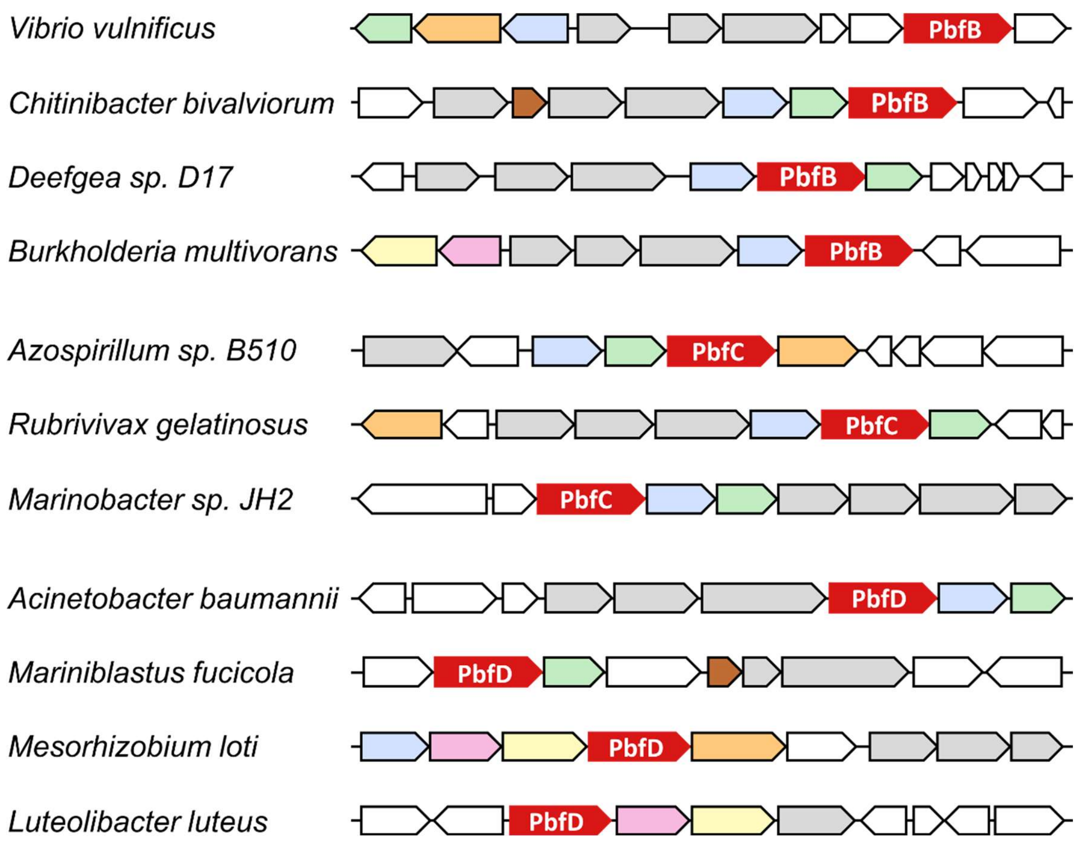

Figure S1 – Recurring presence of genes coding for FAD-dependent oxidoreductases in clusters dedicated to AEP degradation. Related to Figure 2 and Table S1. The genes for the predicted oxidoreductases are shown in red. Other highlighted genes: *phnW* (light blue), *phnX* (light green), *phnA* (pink), *phnY* (yellow) and *pbfA* (orange). Putative phosphonate-related transporter genes are shown in grey. The dark brown genes in the *Chitinobacter bivalviorum* and *M. fucicola* clusters are homologs of *phnZ* (another gene presumably involved in an *(R)*-HAEP degradation process, alternative to the PbfA reaction[1]).

```
PbfB_ : MRNVNATEQSDSSSSHSHPSFWFKQAIEQEQPPSAKPLQGPLETDVLIVGGGYTG :  55
PbfC_ : ---------------------------------MTTTPAIIDRADIVVVGAGILG :  22
PbfD1 : --------------------------------------MNNAQFDLIVVGAGILG :  17
PbfD2 : ----------------------------------------MPEFDVAIIGGGIVG :  15

PbfB_ : LWTAIMLKEQAPE-KQITVIEKGLCGSGASGANGGCMLTWSTKYPTLKRLFGEAH : 109
PbfC_ : LAVAWALGKALGSGGGRSVLVVDRHPPATQATARAAALLTRARGDAATAALVRGT :  77
PbfD1 : LSAAIQAQEQG---LKVCIFEKNAKPVGATRRNFGMVGTSTLTHPEQQ-WRKYAL :  68
PbfD2 : LANAWMASRCN---LSVAVFERDRVASGASVRNFGMVWP--VGQPGE--LSELAM :  63

PbfB_ : AKWLVEQSEQAVLDIEAFCQRHQIDAQLSSKGVYYTATNSAQKGALQPVVAELER : 164
PbfC_ : YAAIAGLEAELDGDLGLRRVGTLHVAASPARVDALRALVAASPDPVDWLDGAGAA : 132
PbfD1 : ETRSFYQRIQAETDISFEQRQGVYLANTALEWQVLNEFAERANSYQIPVHLFSHE : 123
PbfD2 : QSREFWLELQHKANLWVNPCGSLHLAHHQDEQAVLEEFVQQE-GKQREIELIPAS : 117

PbfB_ : LNINSWRHCEQHELATHSGSPRNVDGHYSAIAATVQPAMLARGLRKVAIEMGVQI : 219
PbfC_ : RIAPCLSAEAVERAAFMPLDGFIDPVRLADAYRRSARRSGVRIRDGVAVRAIRVE : 187
PbfD1 : ELVT--------QFSYLNPAQQFQGGLVFEEDYSVEPHVVGQRLLAYAQSQGVEI : 170
PbfD2 : AIEK--------HSPAANPEGLLVGMFSPHELCVNPAVAISQISHWLEETASVSF : 164

PbfB_ : YEHTPMTALAYG-EPAKVTTPQGEIYAQQVVLALNAWMVEQFPQFKRSIVVVSSD : 273
PbfC_ : HGRVAGIDTGDGLIAAPLVVNAAGAWAAGLAWSAG--IGLPQAPVRSQYWITQVR : 240
PbfD1 : YTNACVVQTQYQQGSCQVRLASGETYRANKVLIC---HGEVIDVLYPDLLQSLNL : 222
PbfD2 : FRNTAVTRVDDG----TIKTGAGEKHQAERIVVC---SGSDFETLFPTHFAAAGL : 212

PbfB_ : MVITQPLAPEAFADAGWKVGSSVLDSRIFVHYYRDTVDGRLMLGKGGNHFSYNNA : 328
PbfC_ : RDLFPPDLPALVMPDAGAYARPELGALLFGLRGRRS----LAFDPARLPDDTAGL : 291
PbfD1 : KRCGLQMALT--QPFHQNLNASLYSGLSISRYP--------AFEICPSHAELVK- : 266
PbfD2 : RKCKLQMLATPKQPNEWALGPHLAGGLTLRHYK--------SFESCPTHTALKNR : 259

PbfB_ : VEPMFQRATRYQDLLRRSFDKLFPSLKGEEFAYSWTGGSDRSATGFPFFDHLAGQ : 383
PbfC_ : DLGDSDGGWQTLEEGWQALARLCPALLQVGIAHYVSGLSTYTADGRFVLGPVPEP : 346
PbfD1 : -ASQQGFIKEFGIHILIKQNEFGELIVGDSHEYHSINEAPQFEQREEINEFIQTY : 320
PbfD2 : IANESPLLDEFGIHVMASQNNNGEVILGDSHVYDDDISP---FDSAEIDRLILEE : 311

PbfB_ : SNVFYGFGYSGNGVAQTRMGGKILSSLVLGIENEWSQCGLAKGPLGQFPPEPFRW : 438
PbfC_ : EGLFMATGCCGAGIAASGGIGRAVAASILGAAGGA-------------------- : 381
PbfD1 : CHEKVGLTLPPIQKRWNGYYLTHEHELACITEAEK-------------------- : 355
PbfD2 : LDKLIRLPDFSIERRWHGIYAKHPTRHVLVADPEP-------------------- : 346

PbfB_ : LGAMMVRNAVRRKEEAEDNEQTPWIWDKWLAKLAGPAGKADKLE : 482
PbfC_ : ---DLSPFAPGRLGAVDPFSPALRDACAAARSGKTAG------- : 415
PbfD1 : --NIFLVSAIAGKGMTTGAGFMKDVLEQNIY------------- : 384
PbfD2 : --NCKIVTATGGAGMTLSFGL----AEQIWKHW----------- : 373
```

Figure S2 – Multiple sequence alignment of four FAD-dependent enzymes analyzed in this study. Related to Figure 2, Table S1, Figure S1 and Figure S3. The enzymes are: PbfB from *V. vulnificus*, PbfC from *Azospirillum sp.* B510, PbfD1 from *A. baumannii* and PbfD2 from *M. fucicola*. A green shade highlights residues that are identical in all four proteins, whereas a yellow shading highlights residues that are identical across three sequences. A light blue shade signals residues that are identical only between PbfD1 and PbfD2.

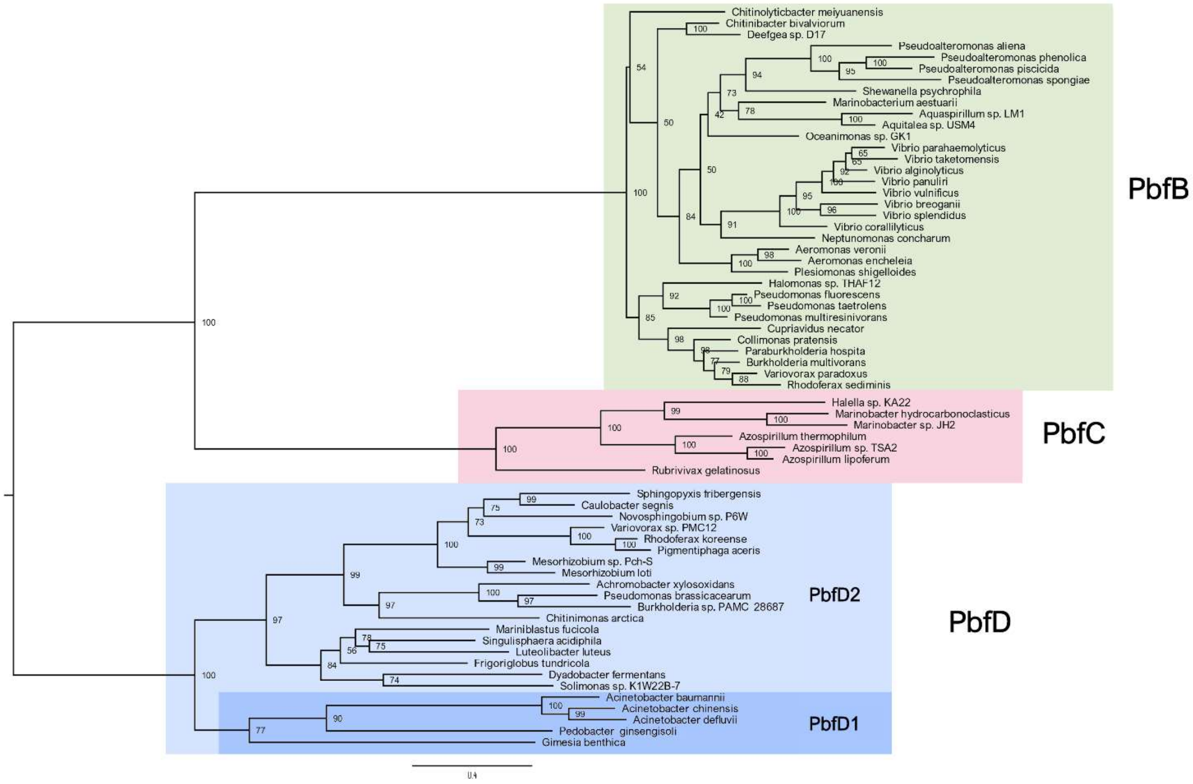

Figure S3 – An alternative representation of the maximum likelihood phylogenetic tree shown in Figure 2 of the main text. Related to Figure 2, Table S1, Figure S1 and Figure S2. The tree was built based on the MSA of 64 FAD-dependent enzymes encoded in gene clusters for the degradation of AEP. Individual enzymes are indicated by the names of the organisms to which they belong, Accession IDs of all sequences are provided in Table S1. Groups of sequences that we labelled PbfB, PbfC and PbfD are highlighted in light green, light red and light blue, respectively. In the case of PbfD, a darker shade of blue signals a subset of enzymes (termed PbfD1 in the text) whose genes usually cluster with *phnW* and *phnX*, whereas the remaining *pbfD* genes (termed *pbfD2*) were most commonly associated with *phnX* alone (see Table S1). Note that while this tree only includes sequences associated to gene clusters for AEP degradation, PbfB, PbfC and PbfD homologs were detected in other bacteria, where they were associated with other genomic contexts. This suggests that the evolutionary origins of these genes may be ancient, predating the split between the major bacterial phyla and the recruitment of these FAD-dependent oxidoreductases by AEP degradation gene clusters. Indeed, the three types of oxidoreductases displayed a markedly different taxonomical range of distribution, even though a complete assessment of their spread is hampered by the frequent occurrence of horizontal gene transfer in prokaryotes [2].

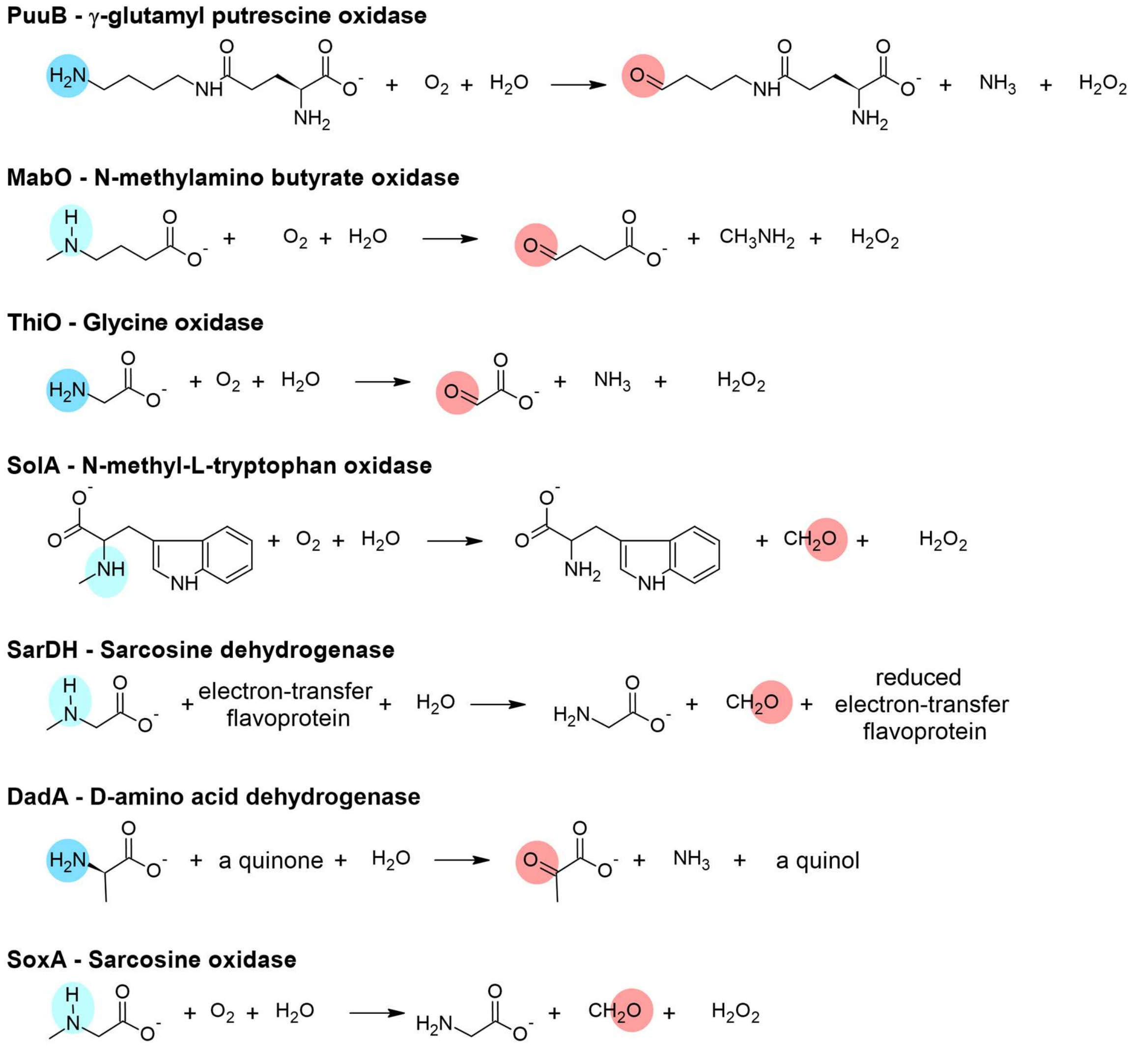

Figure S4 – Reactions catalyzed by the functionally validated FAD enzymes most similar to PbfB, C and D. Related to Table 1 and Table S2. Reacting amino groups are highlighted in light blue (a darker shade signals secondary amines). Carbonyl groups in the products are highlighted in pink.

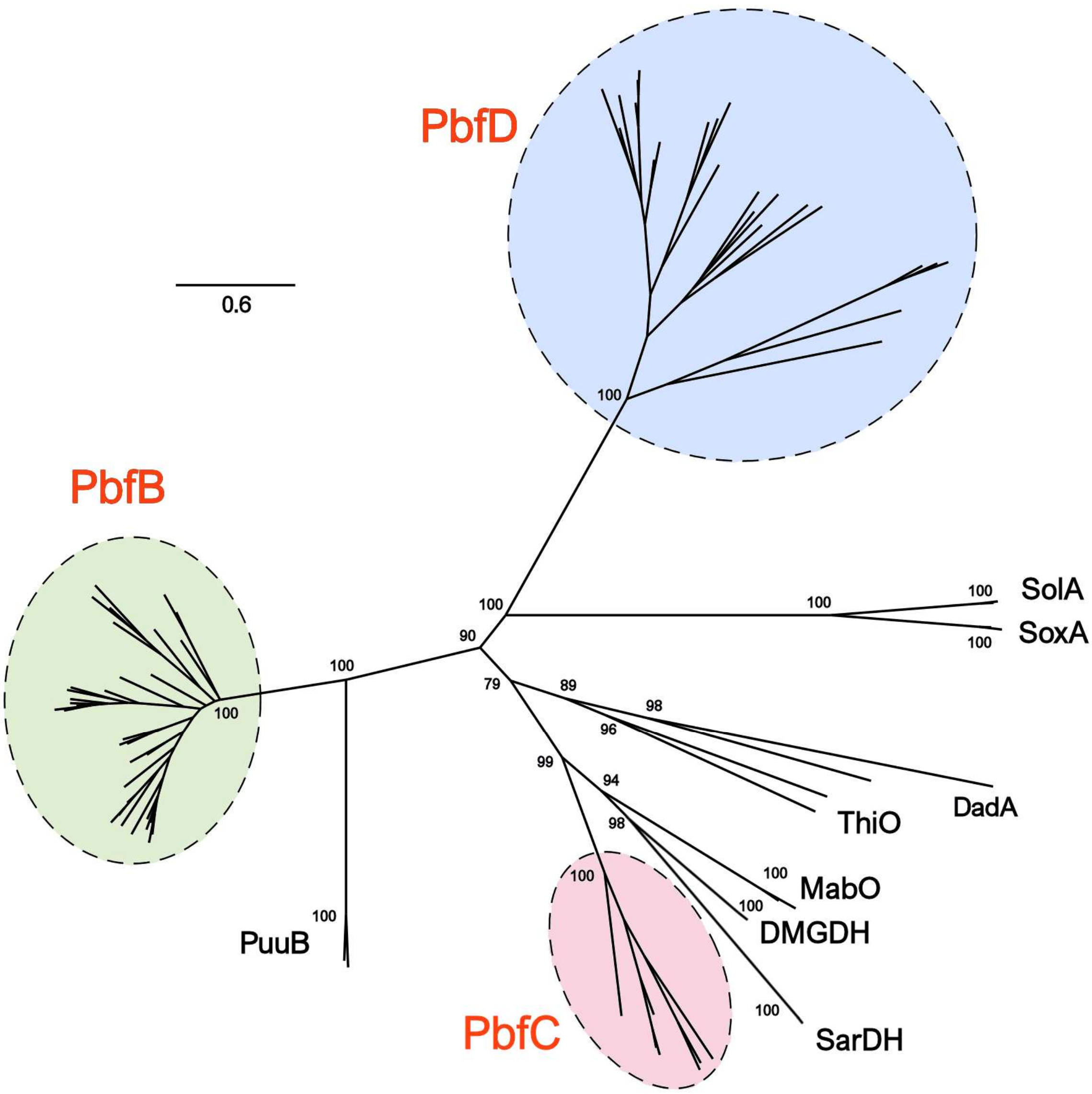

Figure S5 – Phylogenetic relationships between PbfB, PbfC, PbfD and representative members belonging to eight groups of FAD-dependent oxidoreductases of known function. Related to Table 1 and Table S2. See Table S2 for descriptions and accession IDs of these previously characterized oxidoreductases. Only the bootstrap support nodes for the main sequence groups are shown. Although the interpretation of phylogenetic inference is complicated by the existence of other groups of functionally uncharacterized enzymes (which could not be included in the present analysis) this tree clearly shows a tighter relationship between PbfB and γ-glutamyl putrescine oxidase (PuuB), in line with the data reported in Table 1 of the main text. Similarly, the PbfC clade was most closely related with 4-methylamino butyrate oxidase (MabO) and with the mammalian dehydrogenases SarDH and DMDGH, even though these three types of enzymes were much larger than PbfC, displaying additional C-terminal domains in addition to the PF01266 domain. PbfD sequences were largely divergent from all the other enzymes with known function, being most closely related with the oxidases SolA and SoxA.

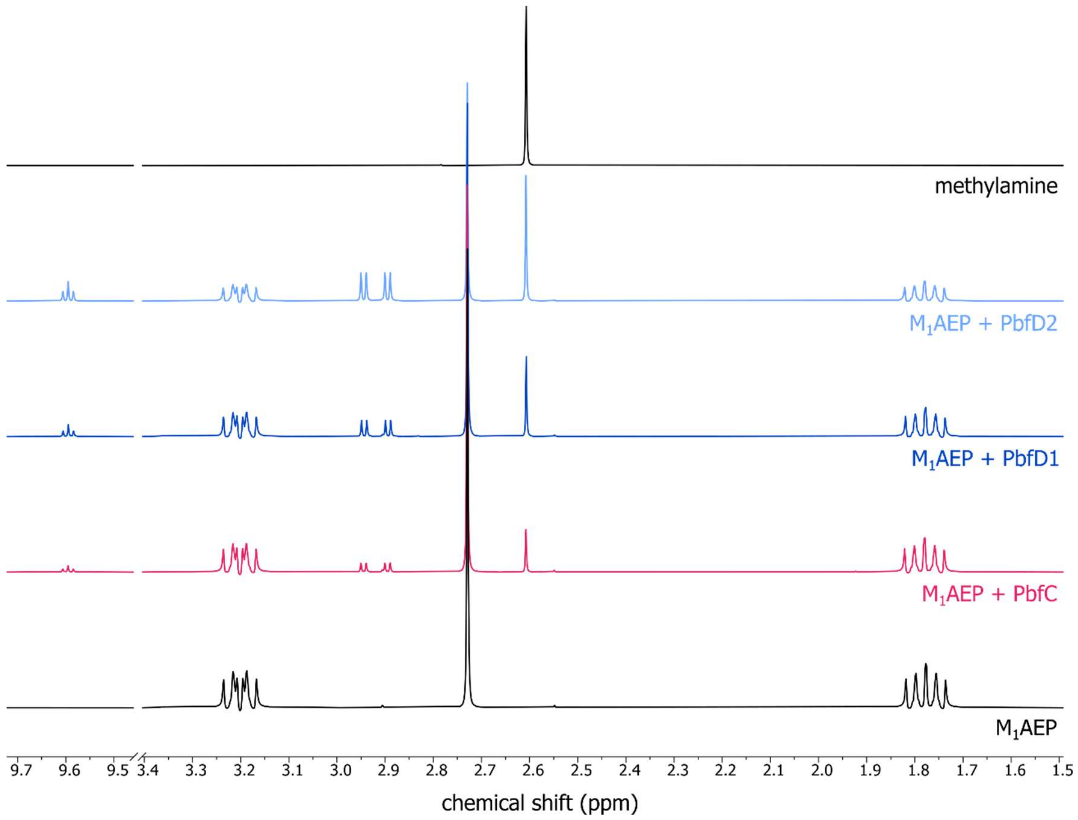

Figure S6 – ^{1}H NMR analysis of the products generated by the oxidoreductases upon the oxidation of M_1AEP. Related to Figure 3. The spectrum of M_1AEP (bottom, black line, 400 MHz, 10% D_2O in water) is compared with the spectra obtained after a 1-hour incubation at room temperature in the presence of either PbfC (red line), PbfD1 (dark blue line) or PbfD2 (light blue line). Reaction conditions are described in the Methods. The new peak appearing at 2.61 ppm upon incubation of M_1AEP with the enzymes is assigned to methylamine, as shown by comparison with a methylamine standard (top spectrum). New peaks at 2.90-2.94 ppm and at 9.6 ppm are attributed to PAA based on published data[3] and on the direct comparison to the spectrum of PAA generated upon transamination of AEP by PhnW or upon deamination of *R*-HAEP by PbfA[4].

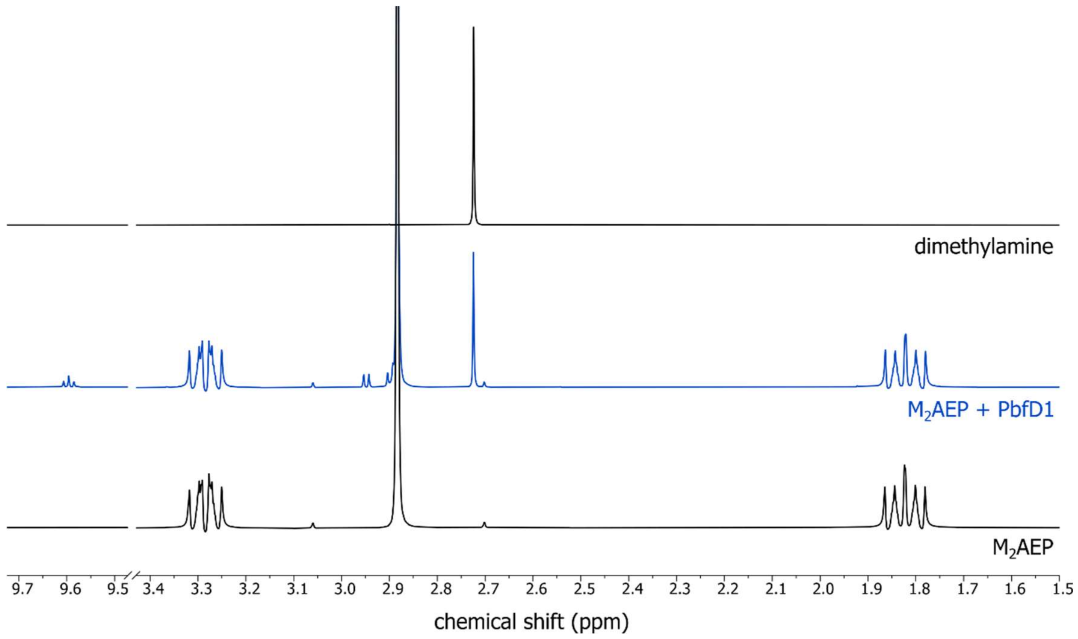

Figure S7 – Analysis of the products generated by PbfD1 upon the oxidation of M_2AEP. Related to Figure 3. The ^{1}H NMR spectrum of M_2AEP (bottom, black line, 400 MHz, 10% D_2O in water) is compared with the spectrum obtained after a 1-hour incubation at room temperature in the presence of PbfD1 (middle, blue line). The top spectrum, provided as a reference, is that of a dimethylamine standard.

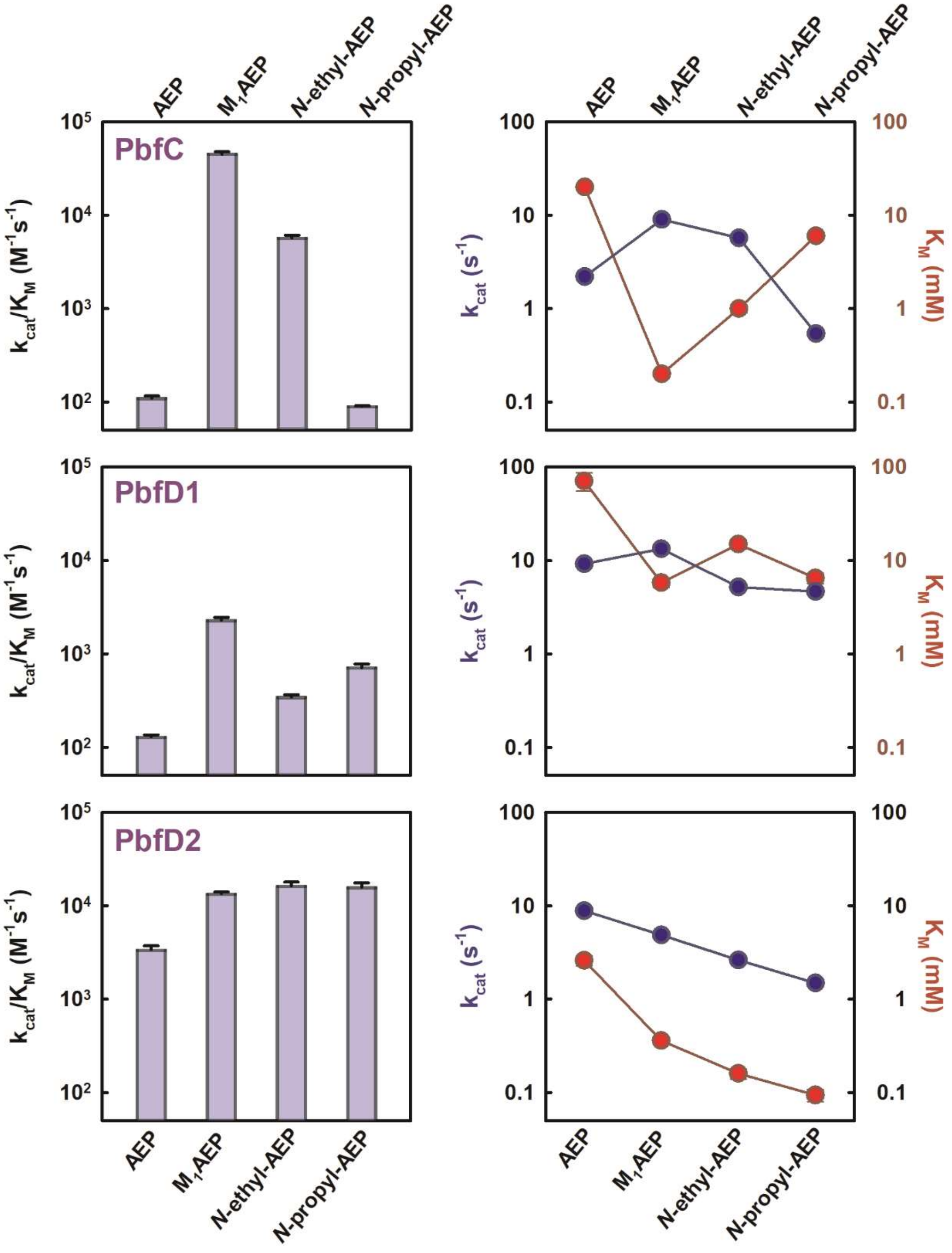

Figure S8 – Apparent catalytic parameters of PbfC (top row), PbfD1 (middle) and PdfD2 (bottom) towards AEP and different N-monoalkylated derivatives. Related to Table 2. The activity of PbfC was measured through the DCPIP assay, as described in the Methods, whereas the activities of PbfD1 and PbfD2 were measured through the coupled assay with PhnX and ADH. The high k_{cat}/K_M of PbfD2 towards *N*-propyl-AEP (bottom row, left) contrasts with the apparent lack of activity observed in the preliminary microtiter assays (Fig. 2 of the main text). We note however that the good k_{cat}/K_M reported here arises from a combination of low K_M and low k_{cat} (bottom row, right). The low k_{cat} was presumably limiting the reaction of PbfD2 with *N*-propyl-AEP under the plate assay conditions.

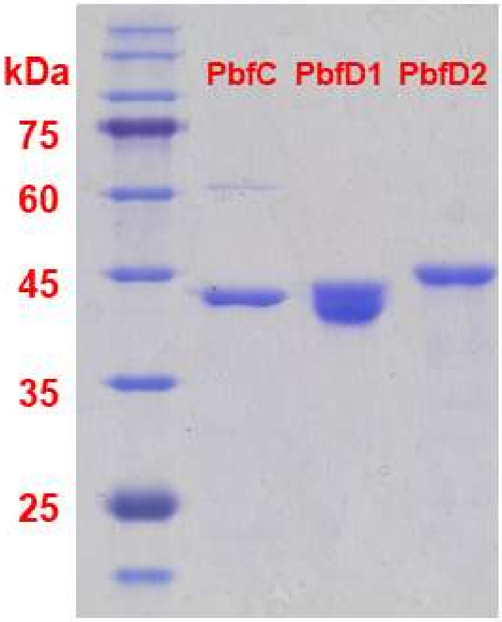

Figure S9 – SDS-PAGE of the three purified oxidoreductases used in this study. Related to STAR Methods. The three enzymes have very similar expected molecular masses (43.3 to 45.6 kDa). PbfD2, despite having the smaller expected mass, showed a slightly lower electrophoretic mobility as compared to the other two oxidoreductases. PbfD1, whose purification yield was much higher than for PbfC or PbfD2, showed up on gel as two close but distinct bands, the lower of which corresponded possibly to a partially digested protein.

Supplemental Schemes

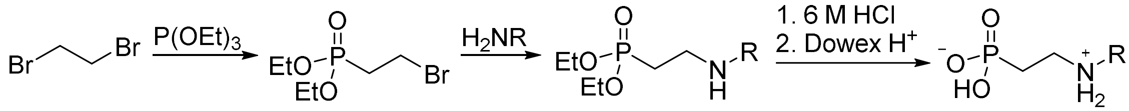

1

2: R = Me
3: R = Et
4: R = *n*Prop

M$_1$-AEP (**5**)**:** R = Me
***N*-ethyl-AEP** (**6**)**:** R = Et
***N*-propyl-AEP** (**7**)**:** R = *n*Prop

Scheme S1 – Synthetic strategy for the synthesis of compounds **5-7**. Related to STAR Methods.

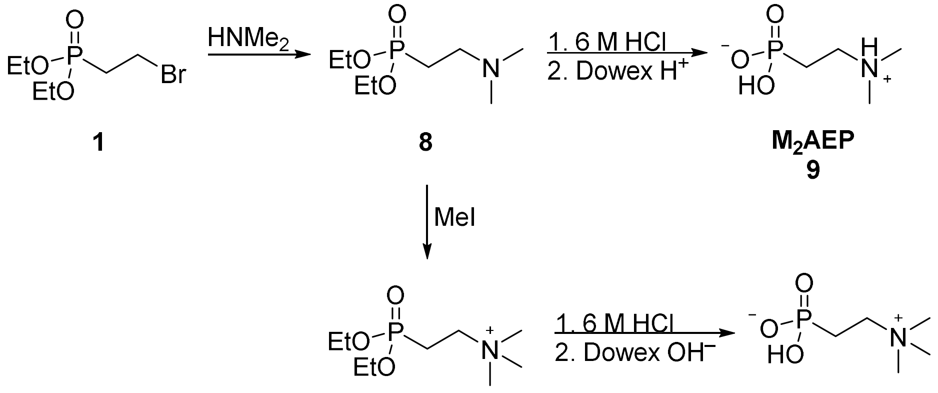

1 **8** **M$_2$AEP 9**

10 as its iodide **M$_3$AEP 11**

Scheme S2 – Synthetic strategy for the synthesis of di- and trimethylated compounds **9-11**. Related to STAR Methods.

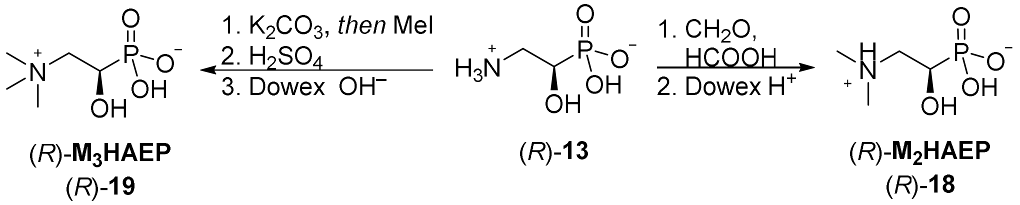

Scheme S3 – Synthesis scheme for the preparation of (*R*)-**19** and (*R*)-**18**. Related to STAR Methods.

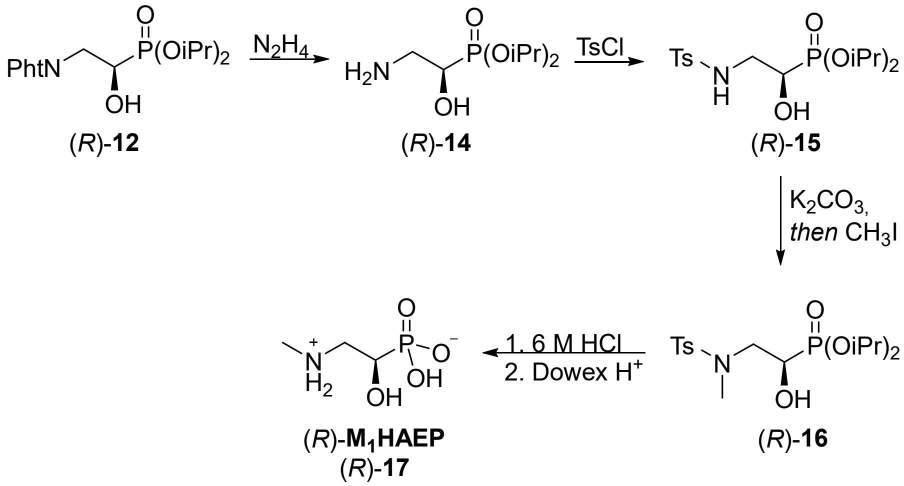

Scheme S4 – Overview of the required steps towards (*R*)-**17**. Related to STAR Methods.

Supplemental Tables

Table S1 - A list of representative FAD-dependent enzymes found within bacterial gene clusters dedicated to AEP degradation. Related to Figure S1, Figure S2, Figure S3 and Figure 2. The sequences were sampled from a larger set of 260 sequences retrieved from genomic analysis of AEP degradation clusters. Enzymes belonging to the PbfB subgroup are highlighted in light green; they were genomically annotated as “FAD-dependent oxidoreductases” in Genbank. Enzymes of the PbfC subgroup are highlighted in pink and were genomically annotated as “FAD-binding oxidoreductases”. Enzymes of the PbfD subgroup are highlighted in light blue; they were genomically annotated as “TIGR03364 family FAD-dependent oxidoreductases”. Enzymes whose activity was experimentally tested in this study are shown in bold. The rightmost column of the table signals (when is the case) the presence in the cluster of homologs of either *pbfA* (which serves to convert *R*-HAEP to PAA[4]) or *phnZ* (which could serve to degrade *R*-HAEP through a different route, generating glycine[1,5,6]).

Organism	GenBank	Group	Cluster type	Other genes in cluster
Vibrio vulnificus	**WP_049798008**	**PbfB**	*phnWX*	*pbfA*
Vibrio splendidus	WP_114635535	PbfB	*phnWX*	*pbfA*
Vibrio panuliri	WP_075714530	PbfB	*phnWX*	*pbfA*
Vibrio parahaemolyticus	WP_023624804	PbfB	*phnWX*	*pbfA*
Vibrio breoganii	WP_065210882	PbfB	*phnWX*	*pbfA*
Vibrio taketomensis	WP_162064033	PbfB	*phnWX*	*pbfA*
Vibrio alginolyticus	WP_158173359	PbfB	*phnWX*	*pbfA*
Vibrio coralliilyticus	WP_040121485	PbfB	*phnWX*	*pbfA*
Variovorax paradoxus	WP_013540189	PbfB	*phnWYA*	*phnZ*
Halomonas sp THAF12	WP_152478592	PbfB	*phnWX*	*phnZ*
Chitinolyticbacter meiyuanensis	WP_148715454	PbfB	*phnWX*	*phnZ*
Chitinibacter bivalviorum	WP_179356922	PbfB	*phnWX*	*phnZ*
Pseudoalteromonas aliena	WP_077538279	PbfB	*phnWX*	*phnZ*
Pseudoalteromonas piscicida	WP_088531662	PbfB	*phnWX*	
Pseudoalteromonas phenolica	WP_058029628	PbfB	*phnWX*	
Pseudoalteromonas spongiae	WP_100915390	PbfB	*phnWX*	
Neptunomonas concharum	WP_138987239	PbfB	*phnWX*	
Aeromonas veronii	WP_005352512	PbfB	*phnWX*	*pbfA*
Aeromonas encheleia	WP_042654121	PbfB	*phnWX*	*pbfA*
Marinobacterium aestuarii	WP_067381910	PbfB	*phnWYA*	
Burkholderia multivorans	WP_069220664	PbfB	*phnWYA*	
Collimonas pratensis	WP_061936987	PbfB	*phnWYA*	*phnZ*
Rhodoferax sediminis	WP_142808290	PbfB	*phnWYA*	*phnZ*
Paraburkholderia hospita	WP_007584212	PbfB	*phnWYA*	
Oceanimonas sp. GK1	WP_014290770	PbfB	*phnWX*	
Pseudomonas fluorescens	WP_108562762	PbfB	*phnWX*	
Pseudomonas taetrolens	WP_048379425	PbfB	*phnWX*	
Pseudomonas multiresinivorans	WP_169939418	PbfB	*phnWX*	
Cupriavidus necator	WP_011301975	PbfB	*phnWYA*	
Plesiomonas shigelloides	WP_192438102	PbfB	*phnWX*	
Aquaspirillum sp. LM1	WP_077298912	PbfB	*phnWX*	*phnZ*
Shewanella psychrophila	WP_077754180	PbfB	*phnWX*	
Aquitalea sp. USM4	WP_131354692	PbfB	*phnWX*	*phnZ*
Deefgea sp. D17	WP_173533283	PbfB	*phnWX*	
Azospirillum sp. B510	**WP_012976454**	**PbfC**	*phnWX*	*pbfA*
Azospirillum thermophilum	WP_109323777	PbfC	*phnWX*	
Azospirillum sp. TSA2s	WP_136702807	PbfC	*phnWX*	*pbfA*
Marinobacter nauticus	WP_014420916	PbfC	*phnWX*	
Marinobacter sp. JH2	WP_133005409	PbfC	*phnWX*	
Hahella sp. KA22	WP_127970665	PbfC	*phnWX*	*phnZ*
Rubrivivax gelatinosus	WP_014429345	PbfC	*phnWX*	*pbfA*

Azonexus hydrophilus	WP_076097175	PbfC	*phnWX*	
Acinetobacter calcoaceticus/baumannii	**WP_079548425**	**PbfD**	*phnWX*	
Acinetobacter chinensis	WP_087514017	PbfD	*phnWX*	*phnZ*
Acinetobacter defluvii	WP_171531132	PbfD	*phnWX*	
Gimesia benthica	WP_155364502	PbfD	*phnWX*	*phnZ*
Pedobacter ginsengisoli	WP_099439046	PbfD	*phnYA*	
Mariniblastus fucicola	**WP_075082418**	**PbfD**	*phnX*	*phnZ*
Chitinimonas arctica	WP_143856386	PbfD	*phnWX*	
Sphingopyxis fribergensis	WP_039578031	PbfD	*phnX*	*phnZ*
Burkholderia sp. PAMC 28687	WP_062003298	PbfD	*phnWYA*	*phnZ*
Variovorax sp. PMC12	WP_106935610	PbfD	*phnX*	*phnZ*
Achromobacter xylosoxidans	WP_013392492	PbfD	*phnX*	*phnZ*
Singulisphaera acidiphila	WP_015244020	PbfD	*phnX*	*phnZ*
Novosphingobium sp. P6W	WP_043978321	PbfD	*phnX*	
Rhodoferax koreense	WP_076200146	PbfD	*phnX*	*phnZ*
Dyadobacter fermentans	WP_015812557	PbfD	*phnX*	*pbfA*
Solimonas sp. K1W22B-7	WP_117291115	PbfD	*phnX*	*phnZ*
Pigmentiphaga aceris	WP_148814907	PbfD	*phnX*	*phnZ*
Pseudomonas brassicacearum	WP_003196189	PbfD	*phnX*	*phnZ*
Frigoriglobus tundricola	WP_171470493	PbfD	*phnX*	*phnZ*
Caulobacter segnis	WP_013080291	PbfD	*phnX*	*phnZ*
Luteolibacter luteus	WP_169454538	PbfD	*phnYA*	
Mesorhizobium sp. Pch-S	WP_129413696	PbfD	*phnWYA*	*pbfA*
Mesorhizobium loti	WP_064987910	PbfD	*phnWYA*	*pbfA*

Table S2 - FAD-dependent enzymes (family PF01266) of known function, most similar to PbfB, PbfC and PbfD. Related to Table 1, Figure S4 and Figure S5. The reactions catalysed by these enzymes are shown in Figure S4. The sequences listed in this table were used to build the phylogenetic tree in Figure S5. Some of these sequences (in bold) were also employed in the context of Table 1 of the main text.

Abbreviation	Enzyme name	Organism	GenBank	Ref.
PuuB	γ-glutamyl putrescine oxidase	***Escherichia coli***	**WP_000134870**	[7]
		Shewanella oneidensis	AAN54342	
MabO	4-methylamino butyrate oxidase	***Arthrobacter nicotinovorans***	**WP_016359432**	[8]
		Glutamicibacter nicotianae	WP_141359319	
ThiO	Glycine oxidase	***Bacillus licheniformis***	**WP_003180677**	[9]
		Pseudomonas putida	WP_010951878	
SolA	N-methyl-L-tryptophan oxidase	***Escherichia coli***	**WP_000872833**	[10]
		Citrobacter tructae	QBX80389	
SarDH	Sarcosine dehydrogenase	***Rattus norvegicus***	**NP_446116**	[11]
		Homo sapiens	AAD53398	
DMGDH	Dimethylglycine dehydrogenase	*Rattus norvegicus*	Q63342	[12]
		Homo sapiens	NP_037523	
DadA	D-amino acid dehydrogenase	***Helicobacter pylori***	**WP_000712537**	[13]
		Chromobacterium violaceum	WP_011135466	
SoxA	Sarcosine oxidase	***Bacillus sp.* B-0618**	**BAA03967**	[14]
		Arthrobacter sp. TE1826	BAA09716	

Supplemental Data

Data S1 - Recorded ^{1}H, ^{13}C and ^{31}P NMR spectra of all the compounds synthesized for this study. Related to the STAR methods and to Schemes S1-S4. All assignments, solvents and device parameters are given in the STAR Methods. The first spectrum shown in each series is the full ^{1}H NMR spectrum. Expansions are depicted where they were regarded as necessary. Then the ^{13}C NMR spectrum is depicted in the same manner, followed by the ^{31}P NMR spectrum. The x-axes and peak labels are is in ppm for ^{31}P NMR spectra, while the peak labels are in Hz for all given^{13}C and ^{1}H NMR spectra. Structures are always given on top of the full ^{1}H NMR spectrum. Integrals are denoted below the x-axes where they are regarded as necessary and the integration range is marked. The numbering of compounds is in accordance with the numbering of substances in the main text and in Schemes S1-S4.

^{1}H NMR of Diethyl-(2-bromethyl)phosphonate (**1**)

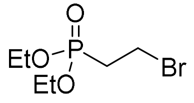

1

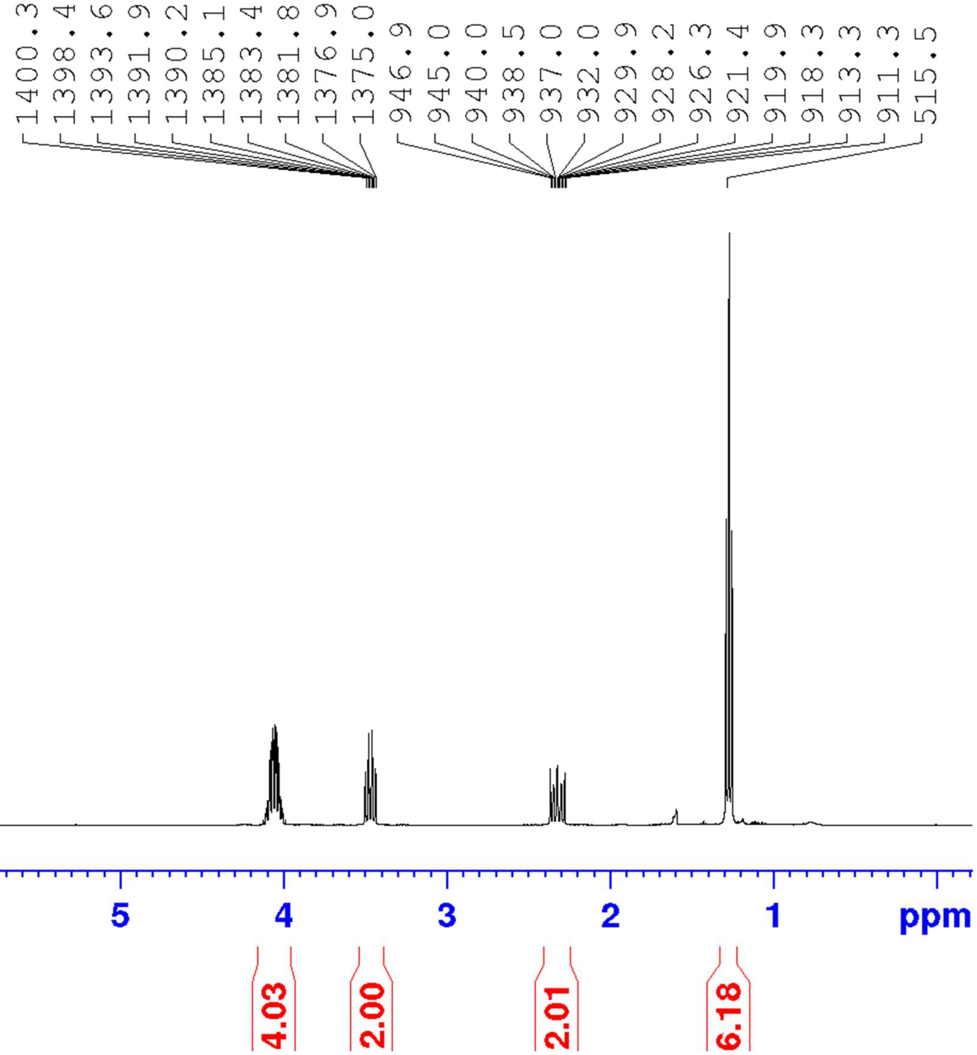

^{13}C NMR of Diethyl-(2-bromoethyl)phosphonate (1)

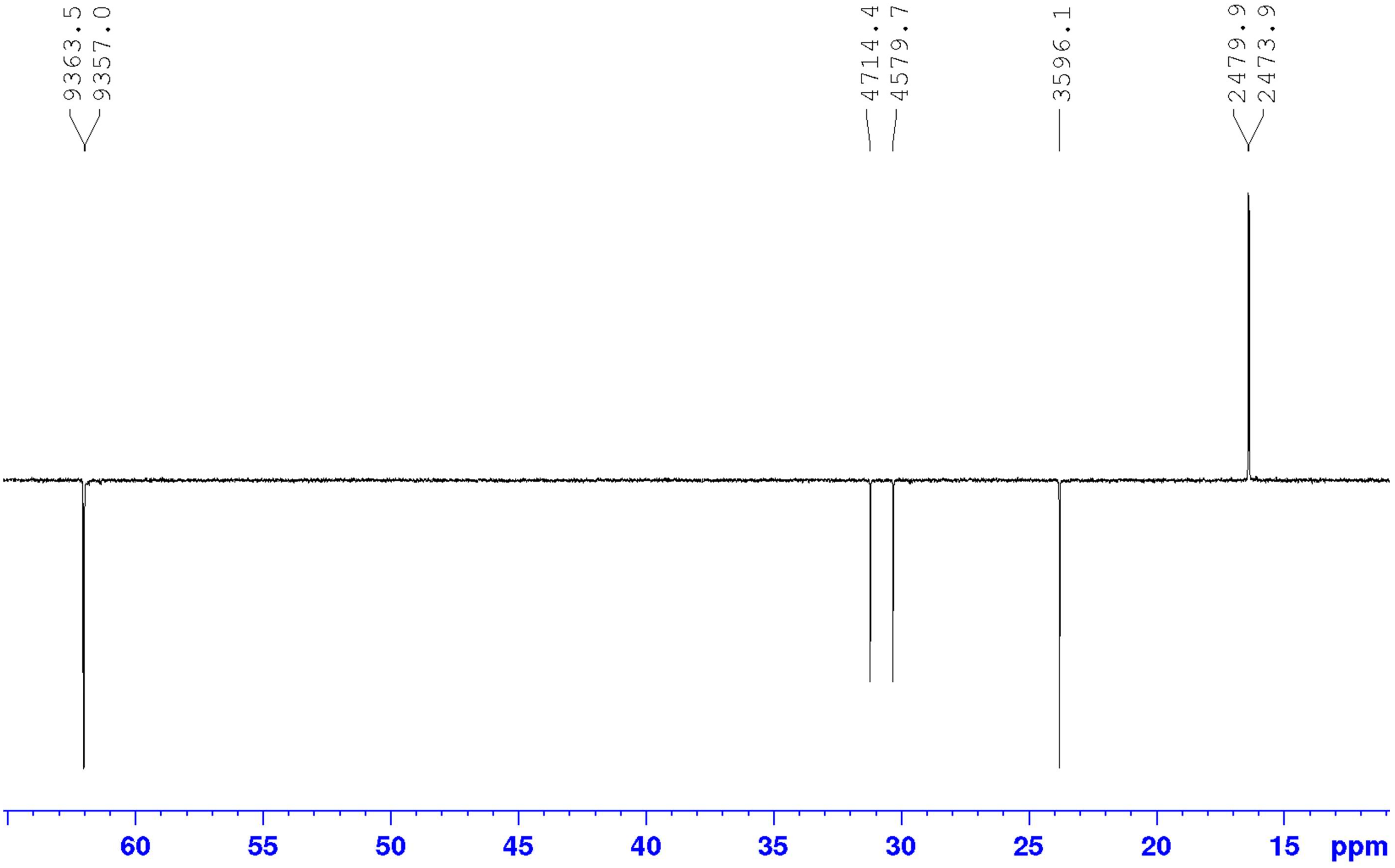

^{31}P NMR of Diethyl-(2-bromoethyl)phosphonate (1)

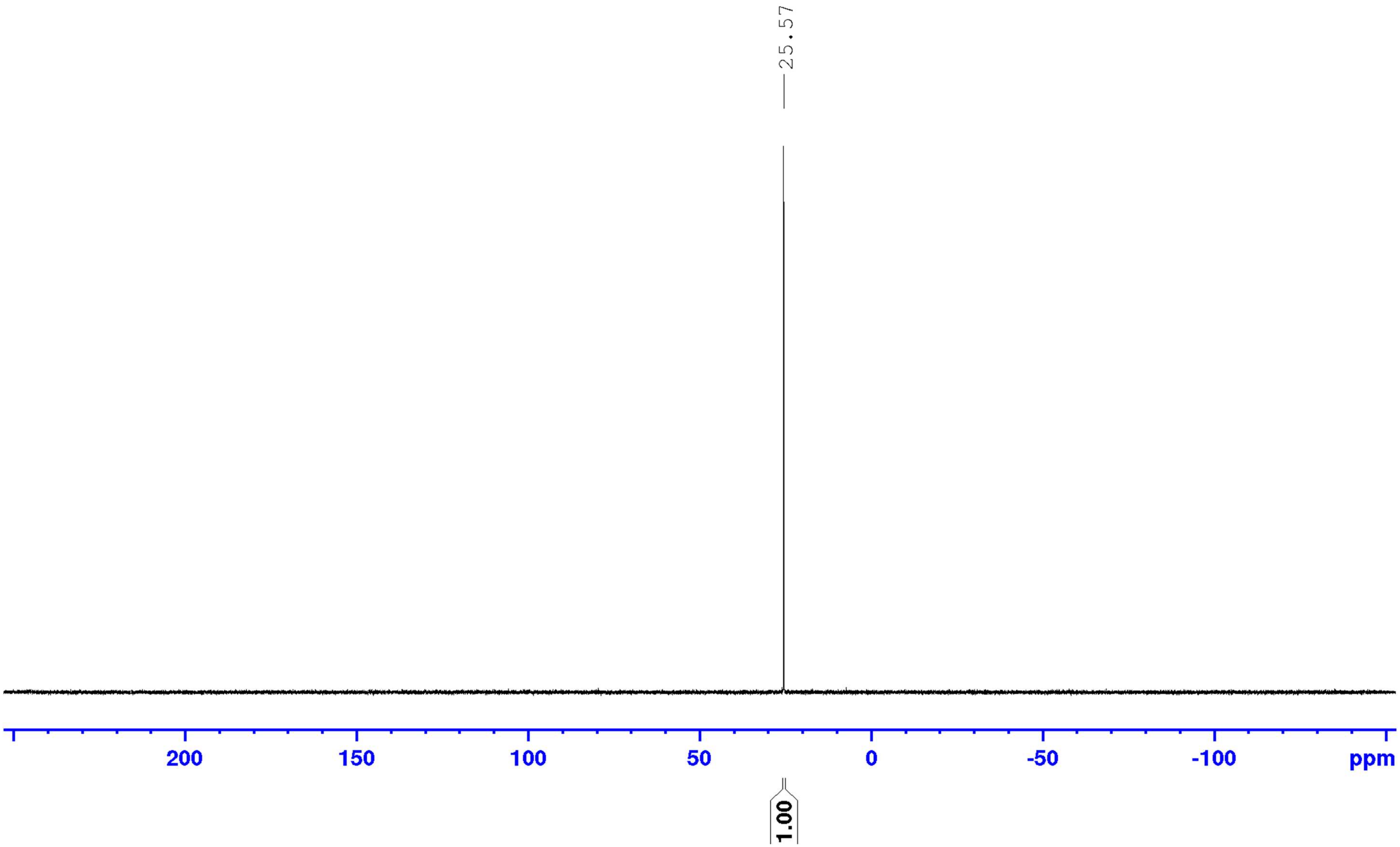

[1]**H NMR of Diethyl 2-methylamino-ethylphosphonate** (**2,** partially as its hydrobromide)

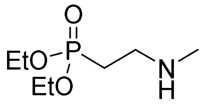

2

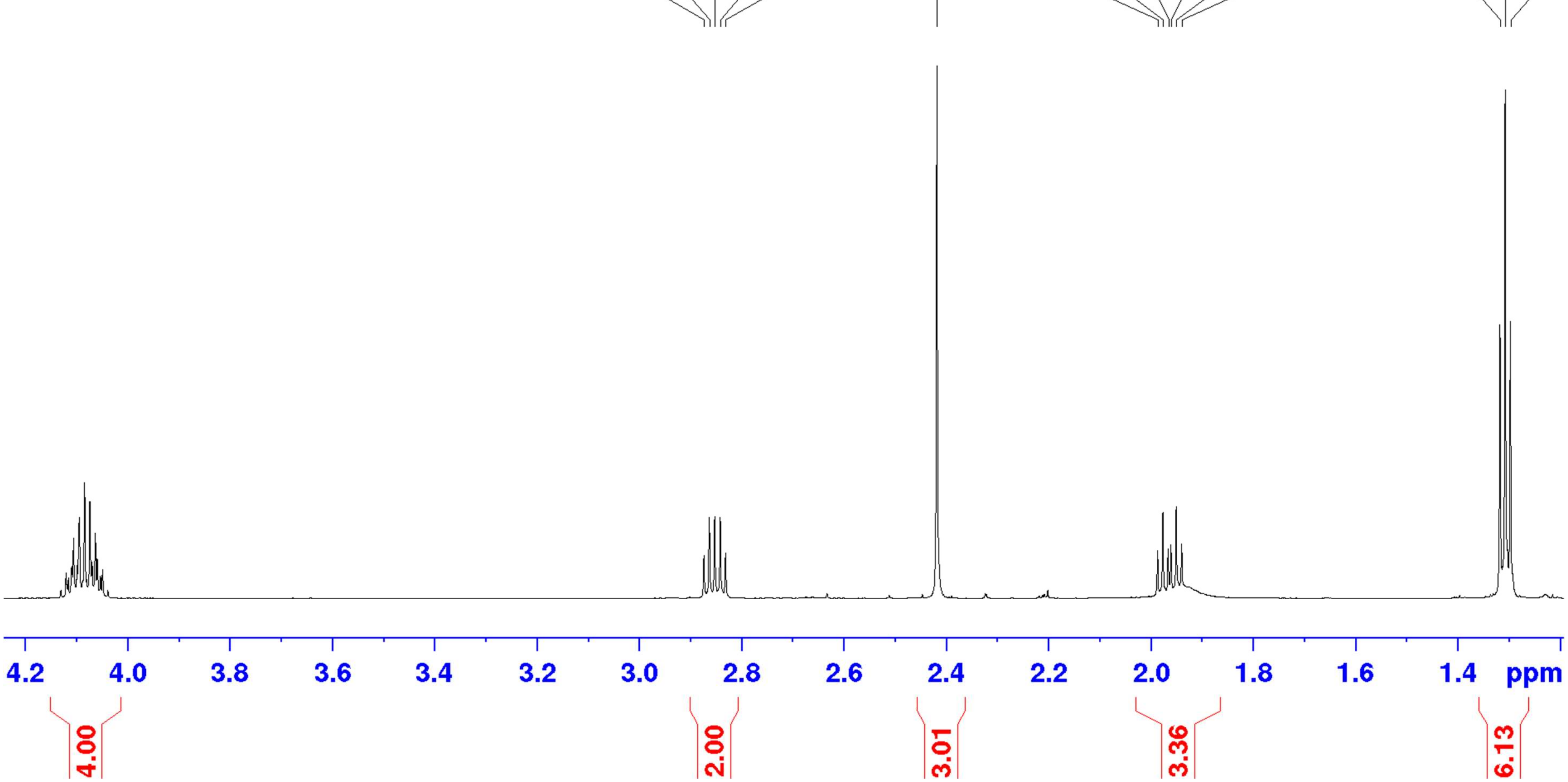

^{13}C NMR of Diethyl 2-methylamino-ethylphosphonate (**2,** partially as its hydrobromide)

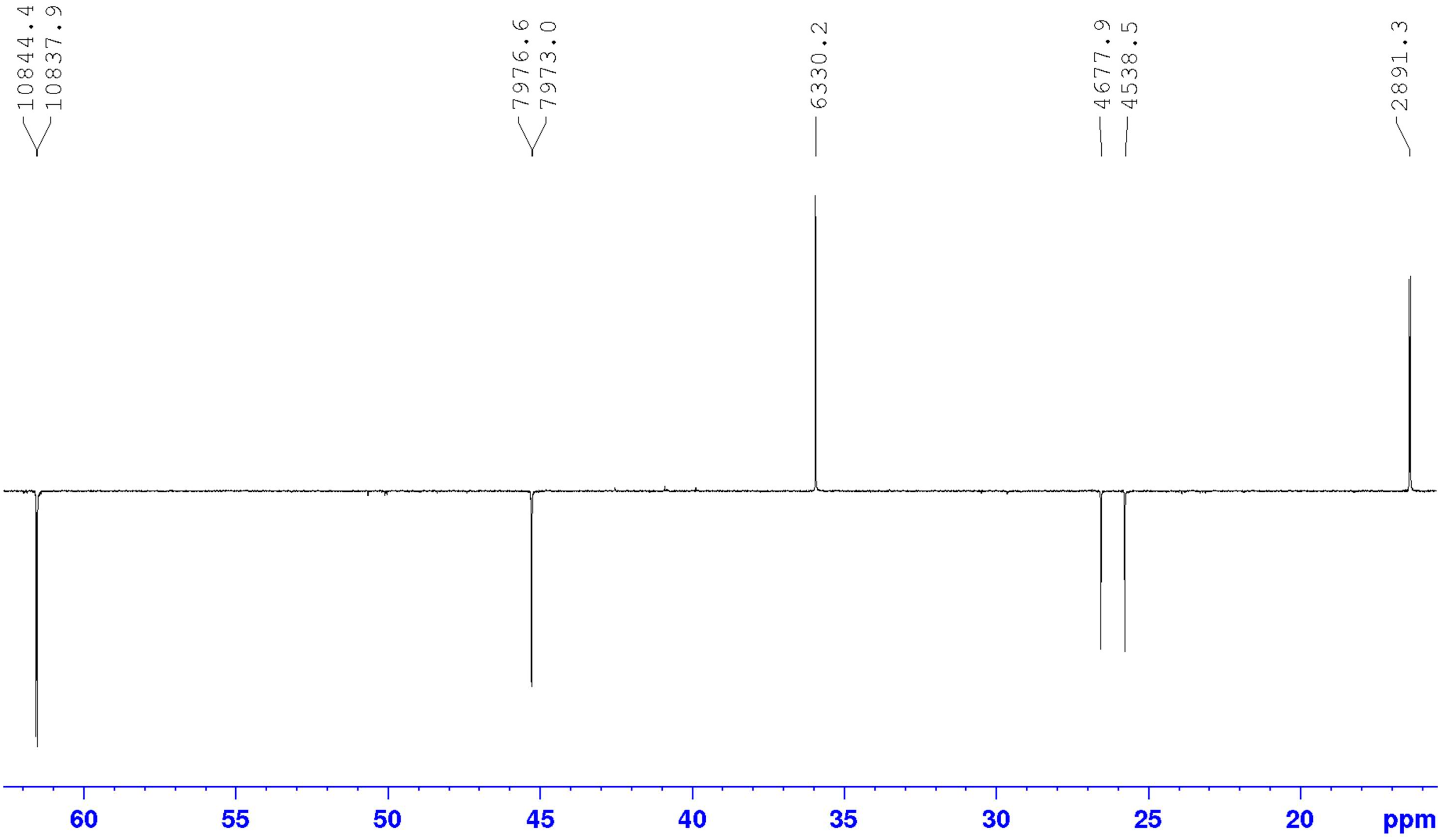

^{31}P NMR of Diethyl 2-methylamino-ethylphosphonate (**2,** partially as its hydrobromide)

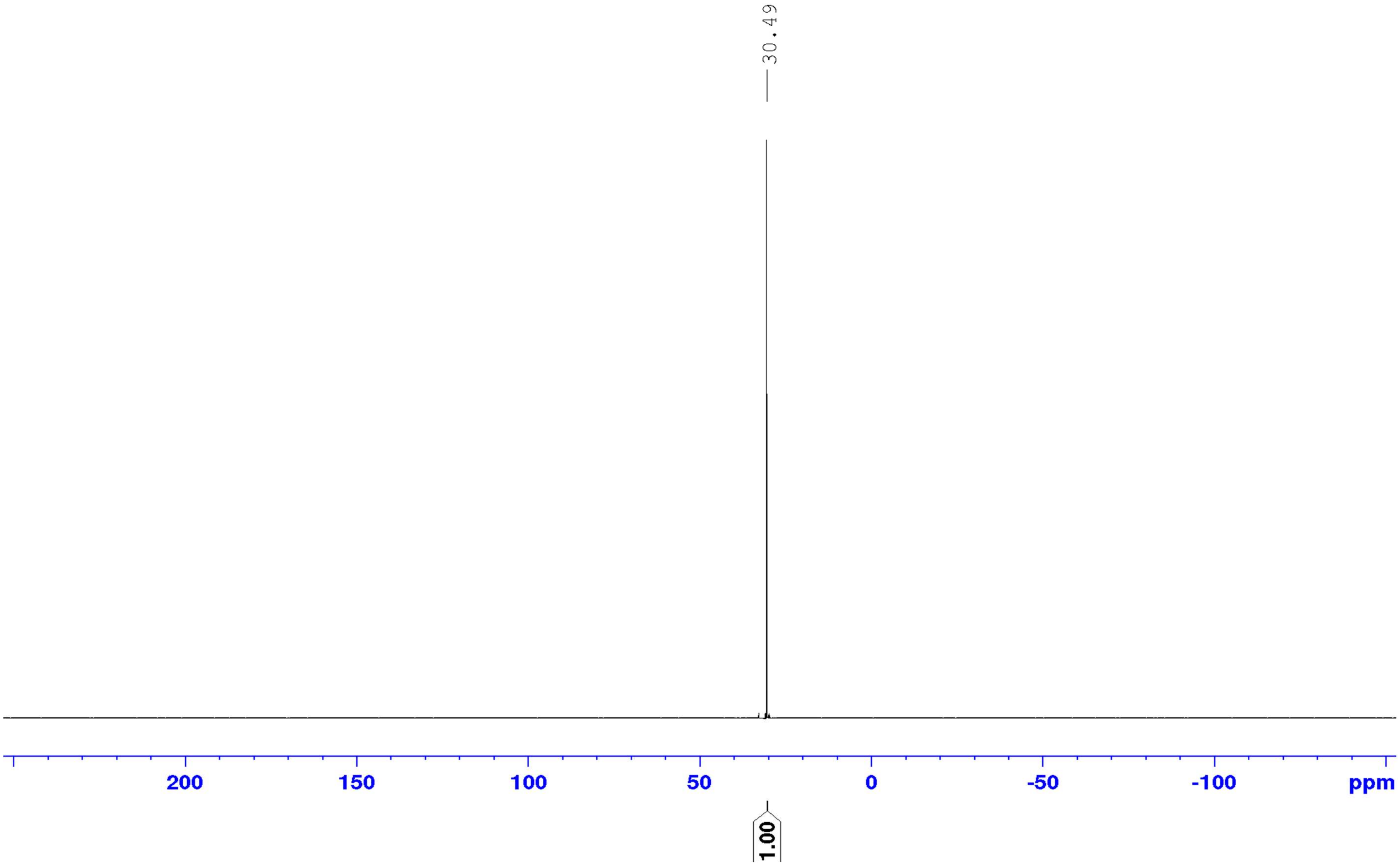

^{1}H NMR of 2-Methylamino-ethylphosphonic acid (5)

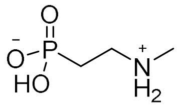

M_1-AEP(5)

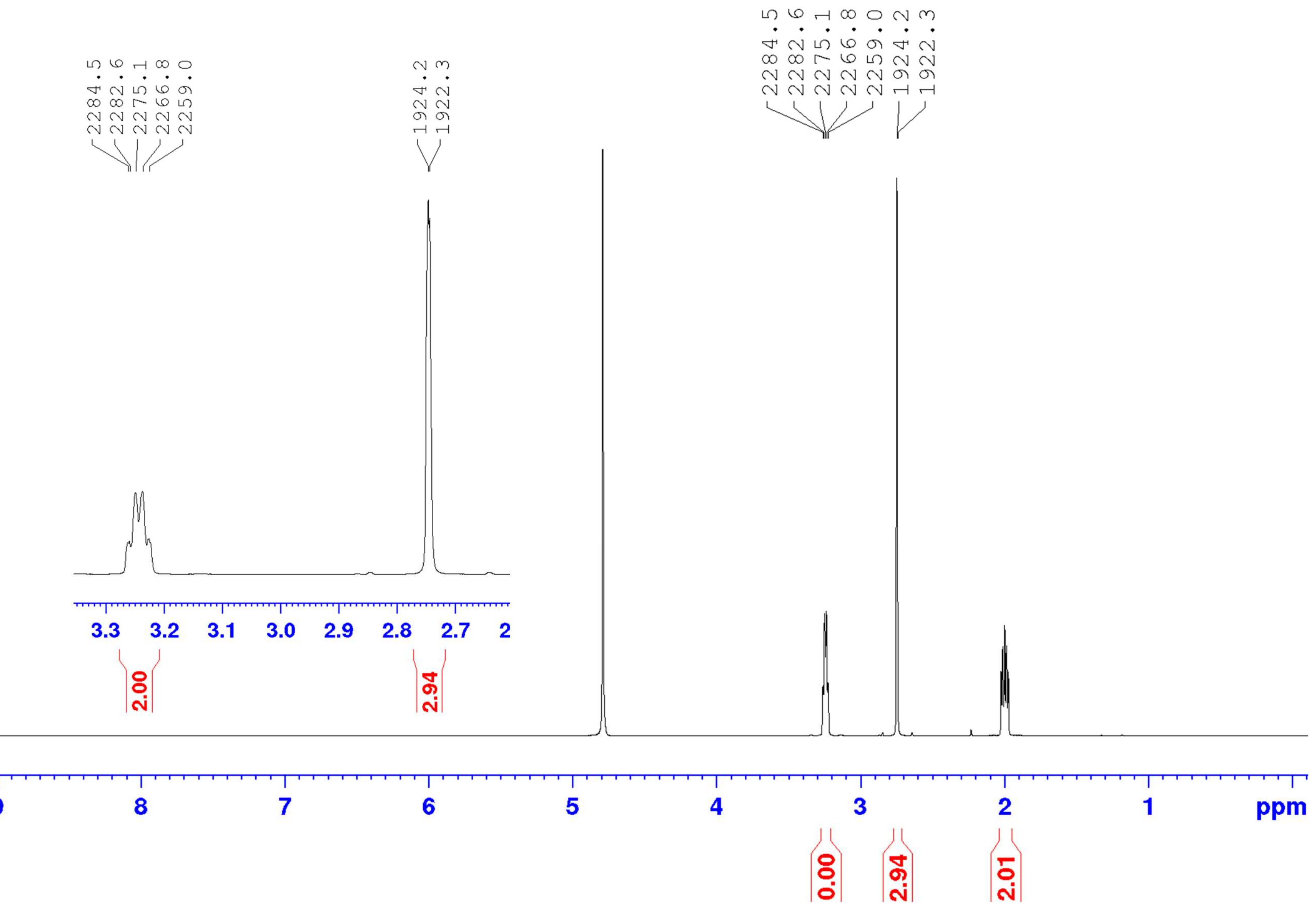

^{13}C NMR of 2-Methylamino-ethylphosphonic acid (5)

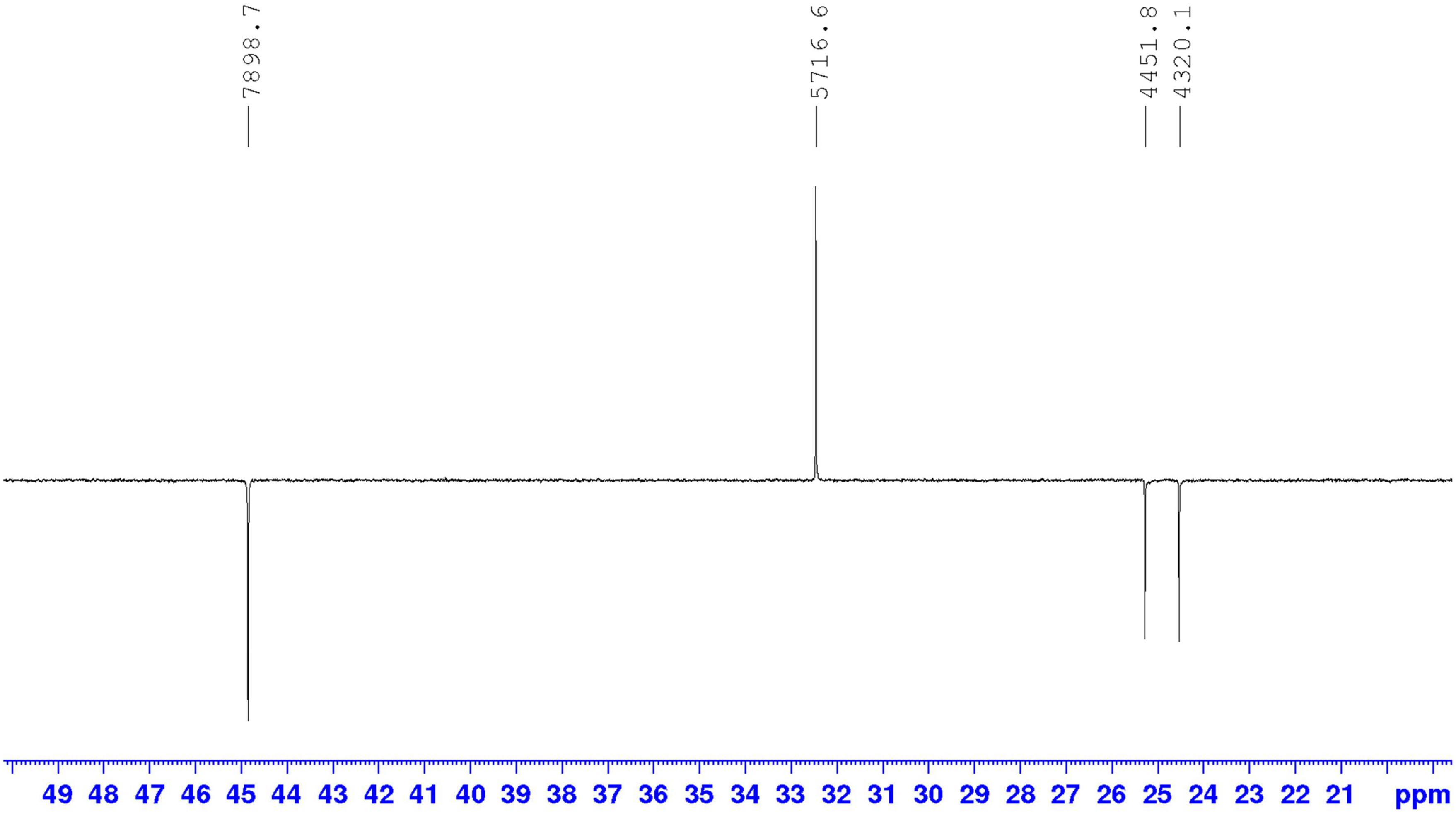

^{31}P NMR of 2-Methylamino-ethylphosphonic acid (5)

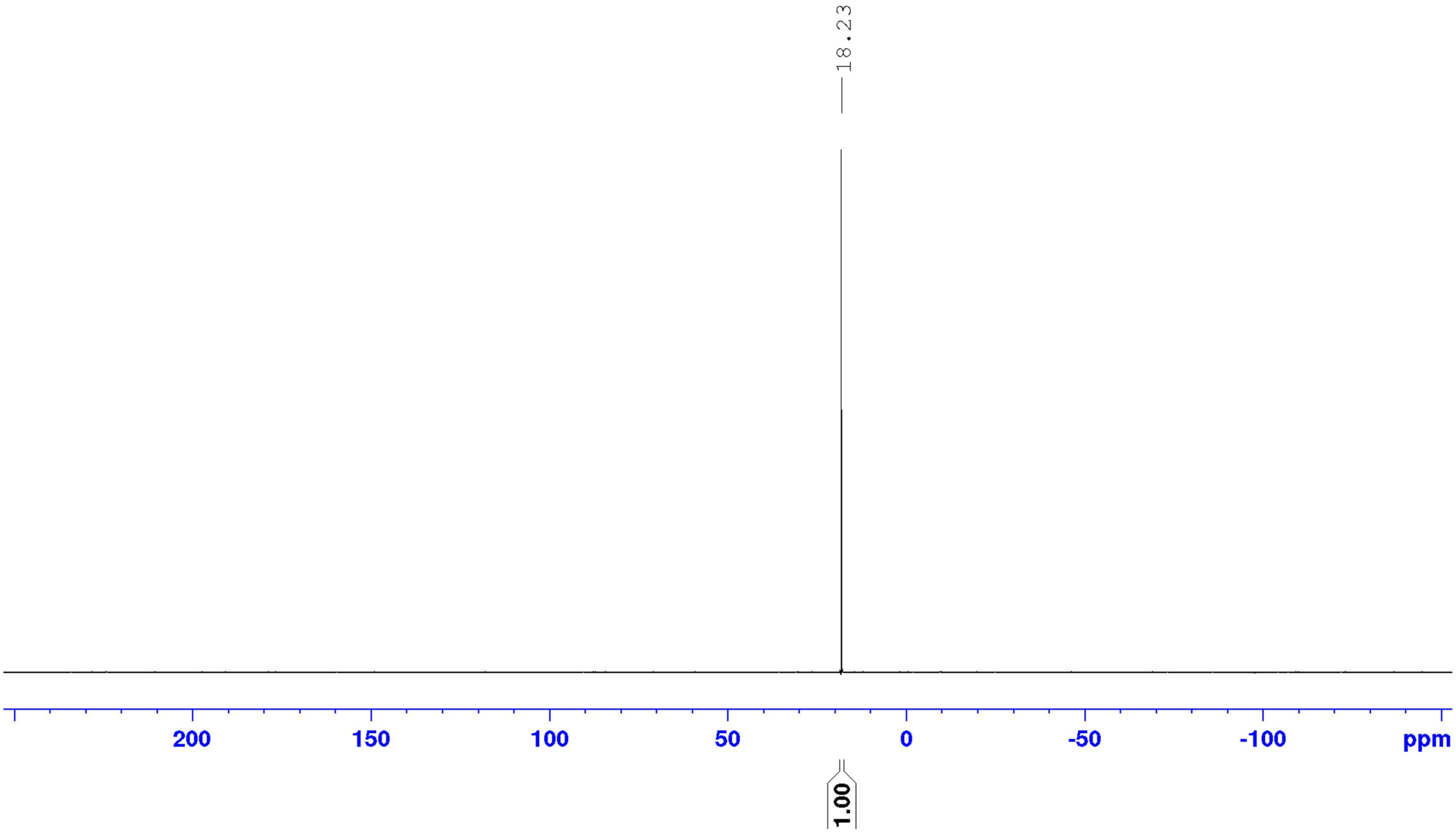

^{1}H NMR of Diethyl 2-ethylamino-ethylphosphonate (**3**, partially as its hydrobromide)

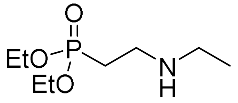

3

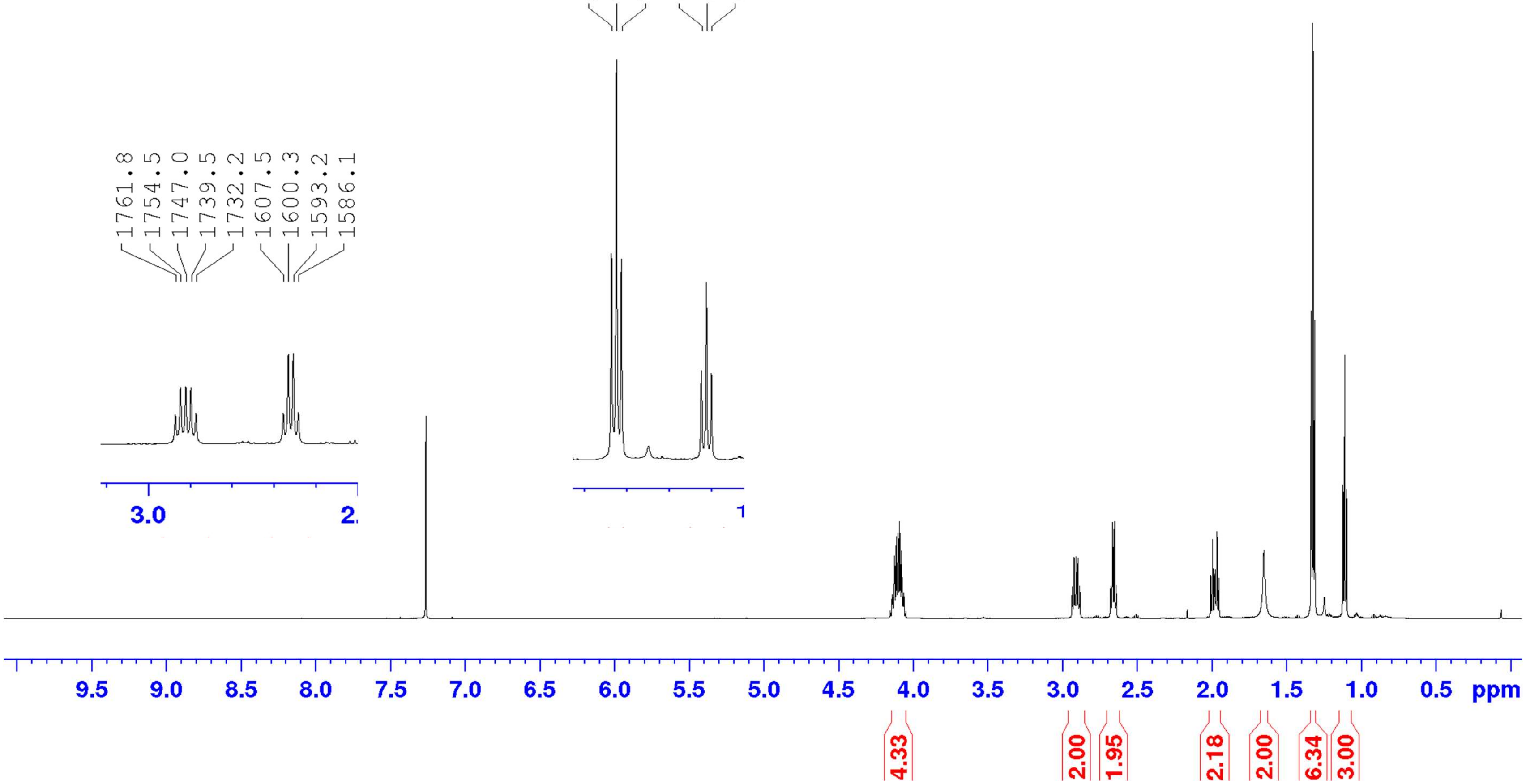

[13]**C NMR of Diethyl 2-ethylamino-ethylphosphonate** (**3**, partially as its hydrobromide)

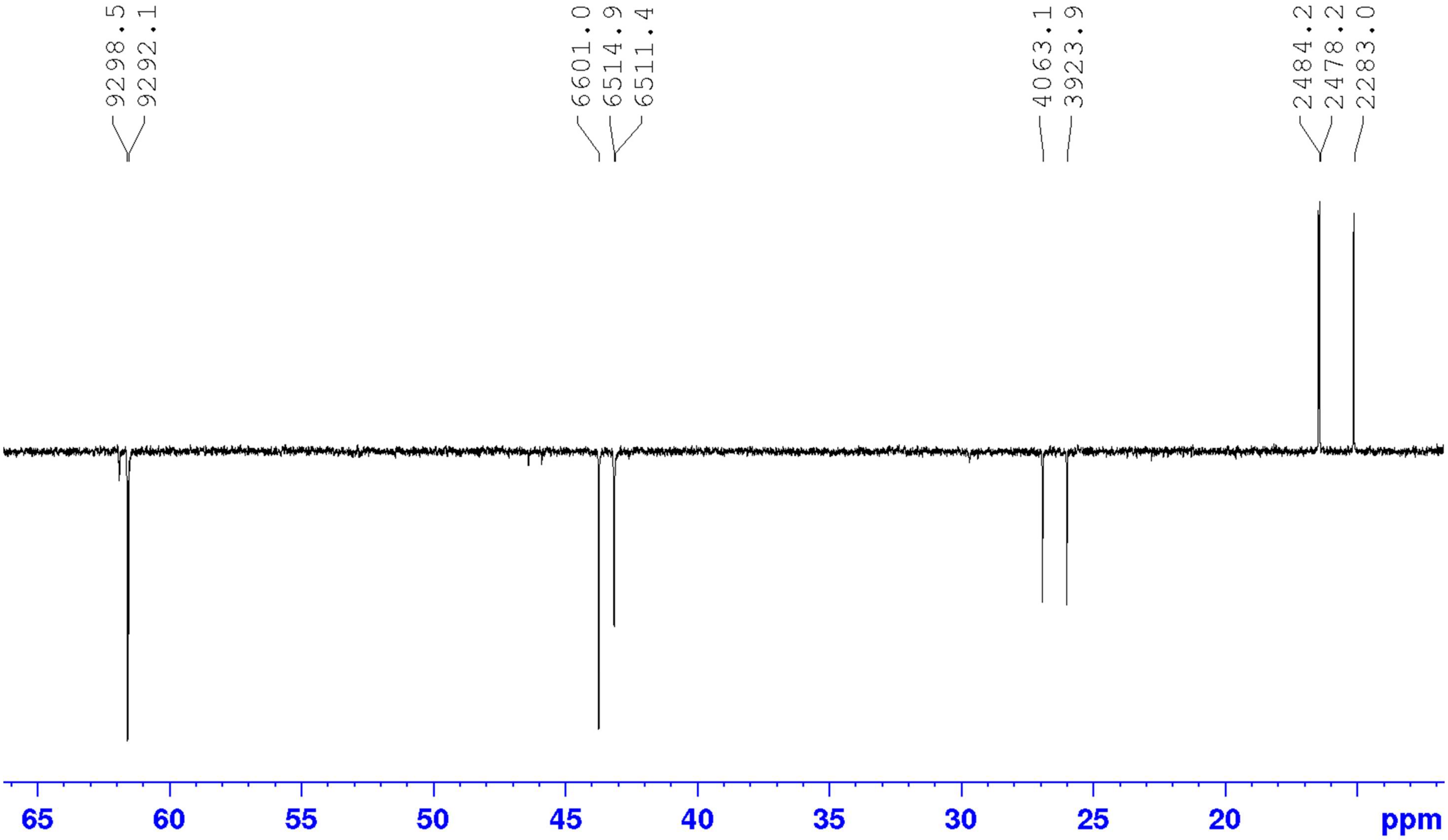

^{31}**P NMR of Diethyl 2-ethylamino-ethylphosphonate** (**3**, partially as its hydrobromide)

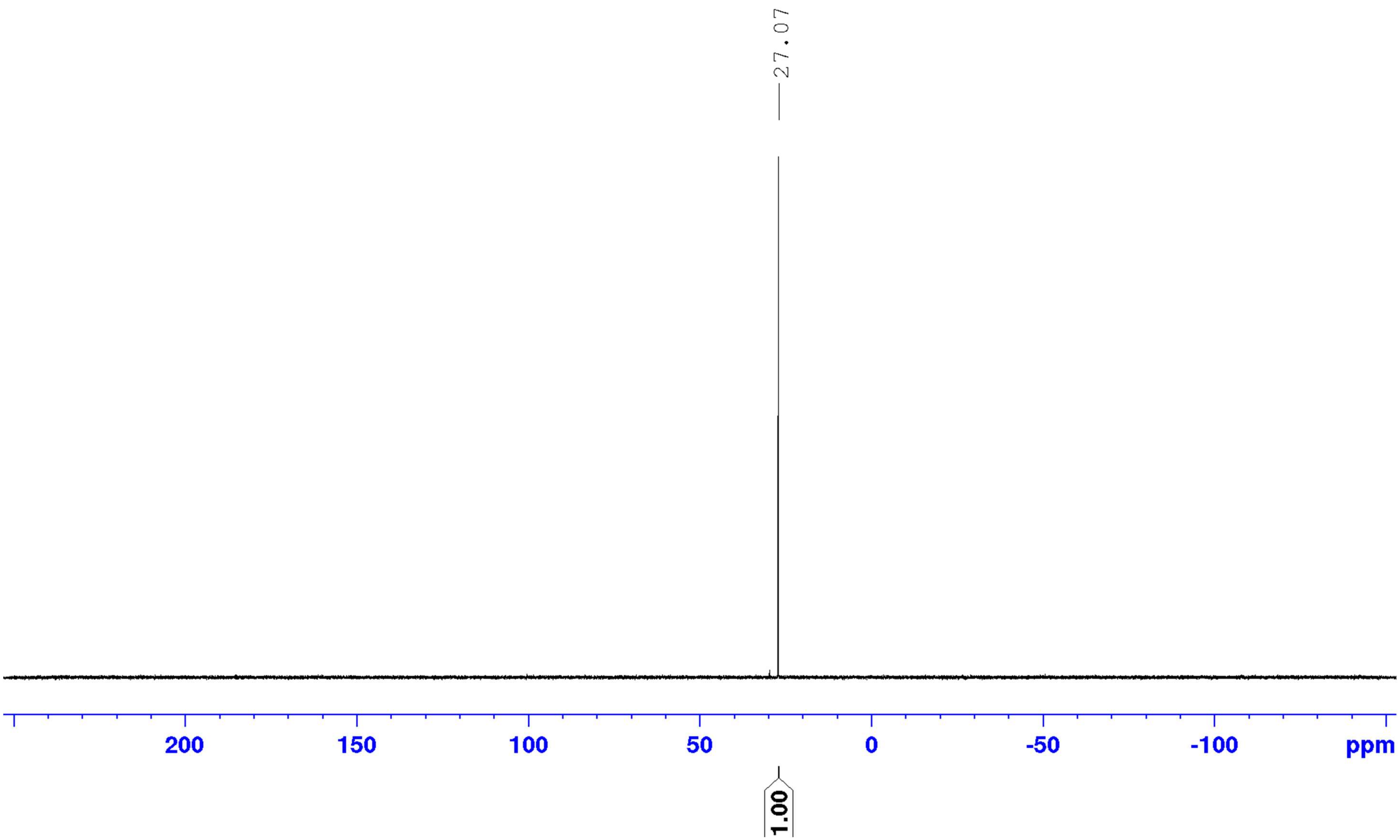

^{1}H NMR of 2-Ethylamino-ethylphosphonic acid (E$_1$-AEP, 6)

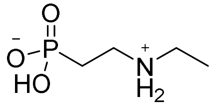

E$_1$-AEP (6)

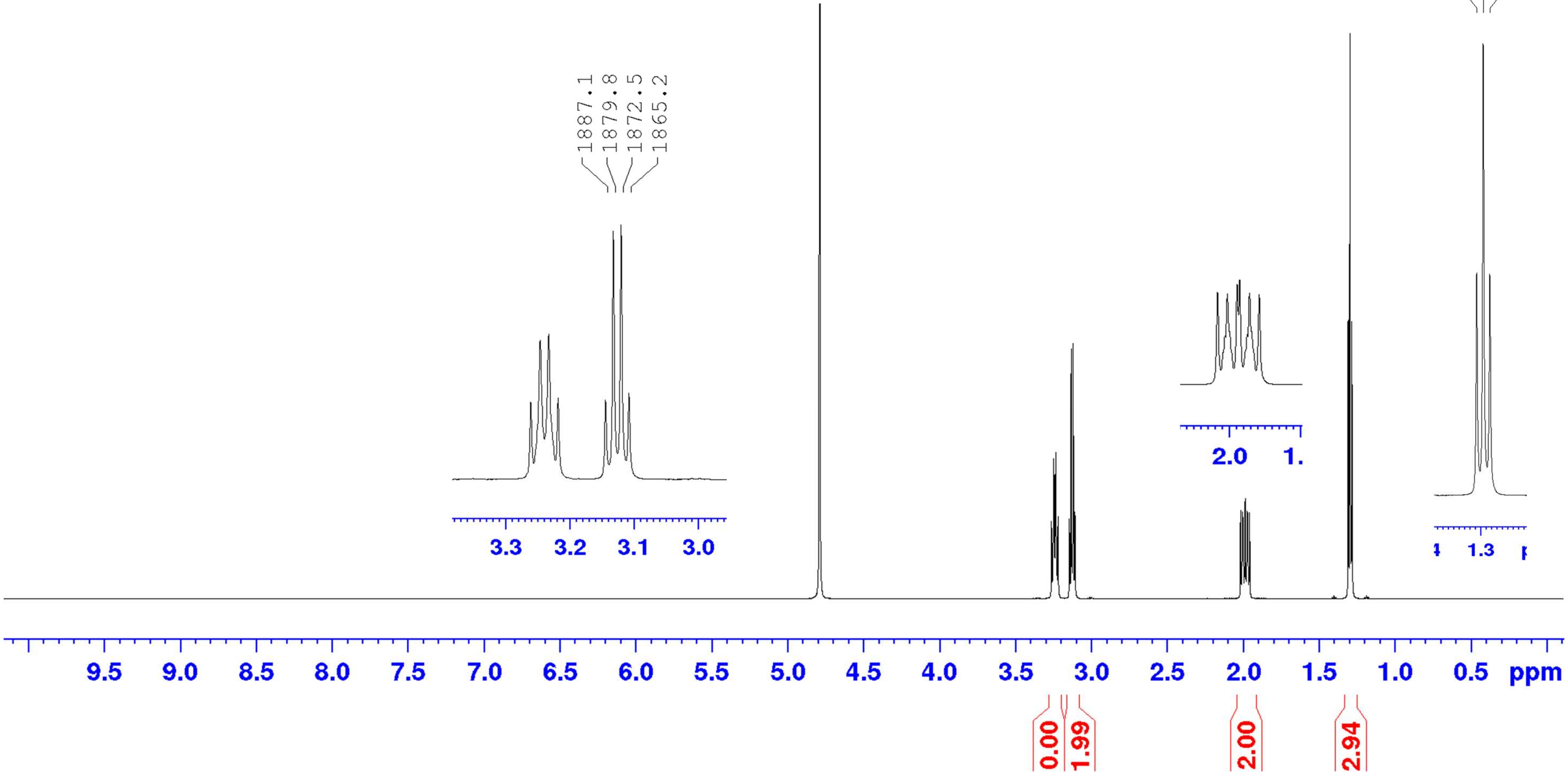

^{13}C NMR of 2-Ethylamino-ethylphosphonic acid (E$_1$-AEP, 6)

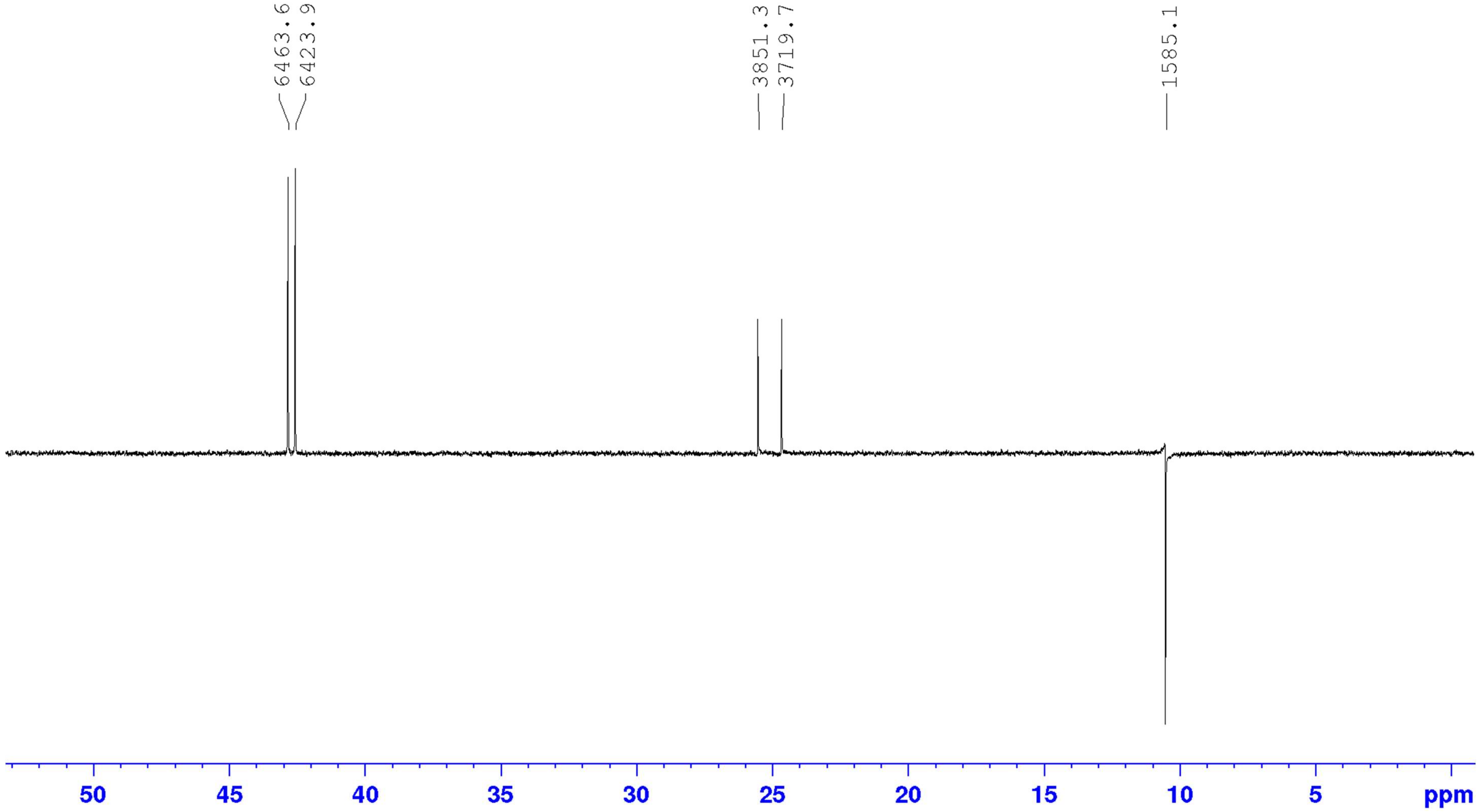

^{31}P NMR of 2-Ethylamino-ethylphosphonic acid (E$_1$-AEP, 6)

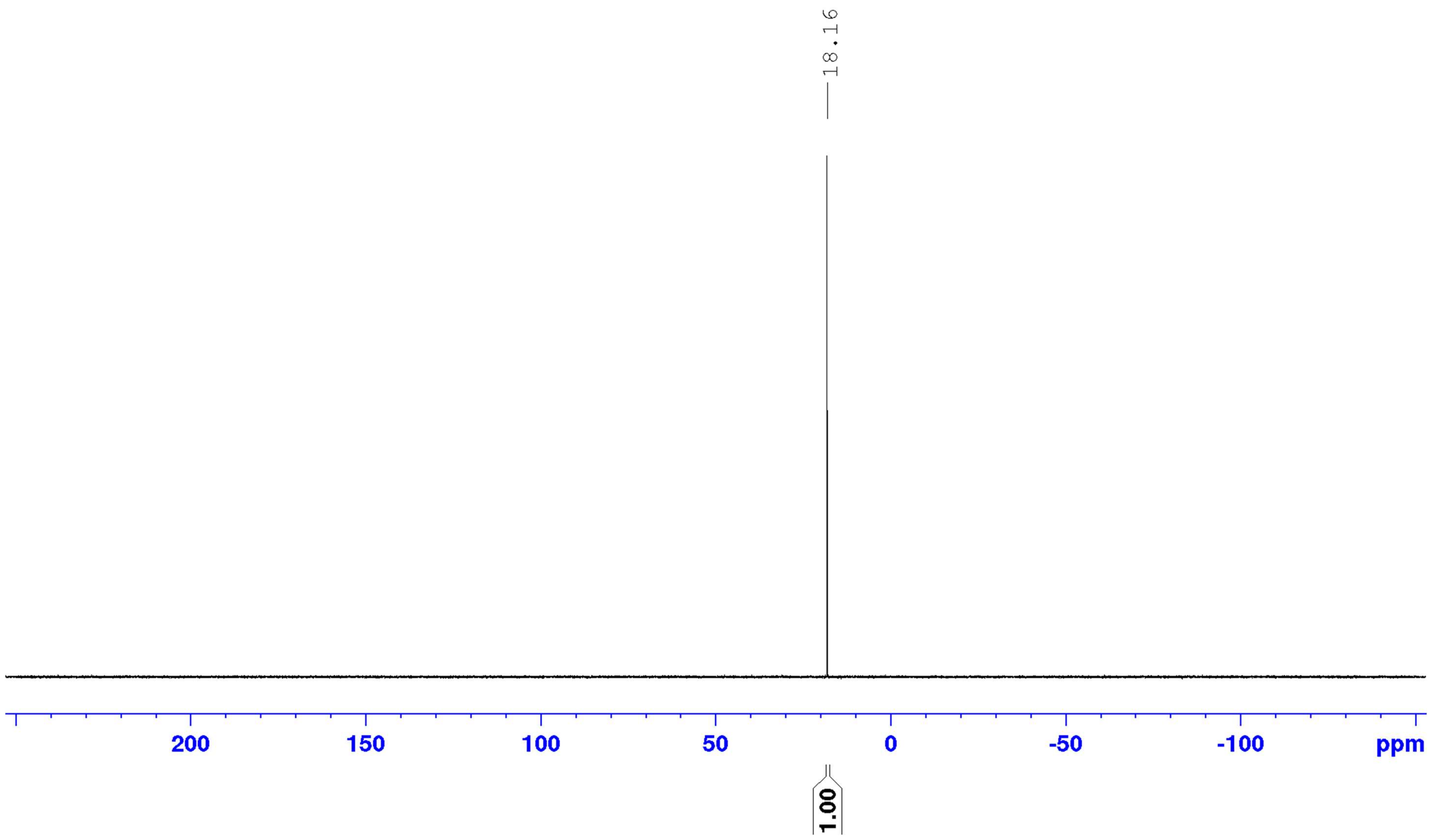

^{1}H NMR of Diethyl 2-propylamino-ethylphosphonate (**4**, partially as its hydrobromide)

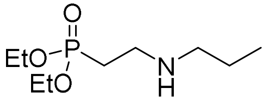

4

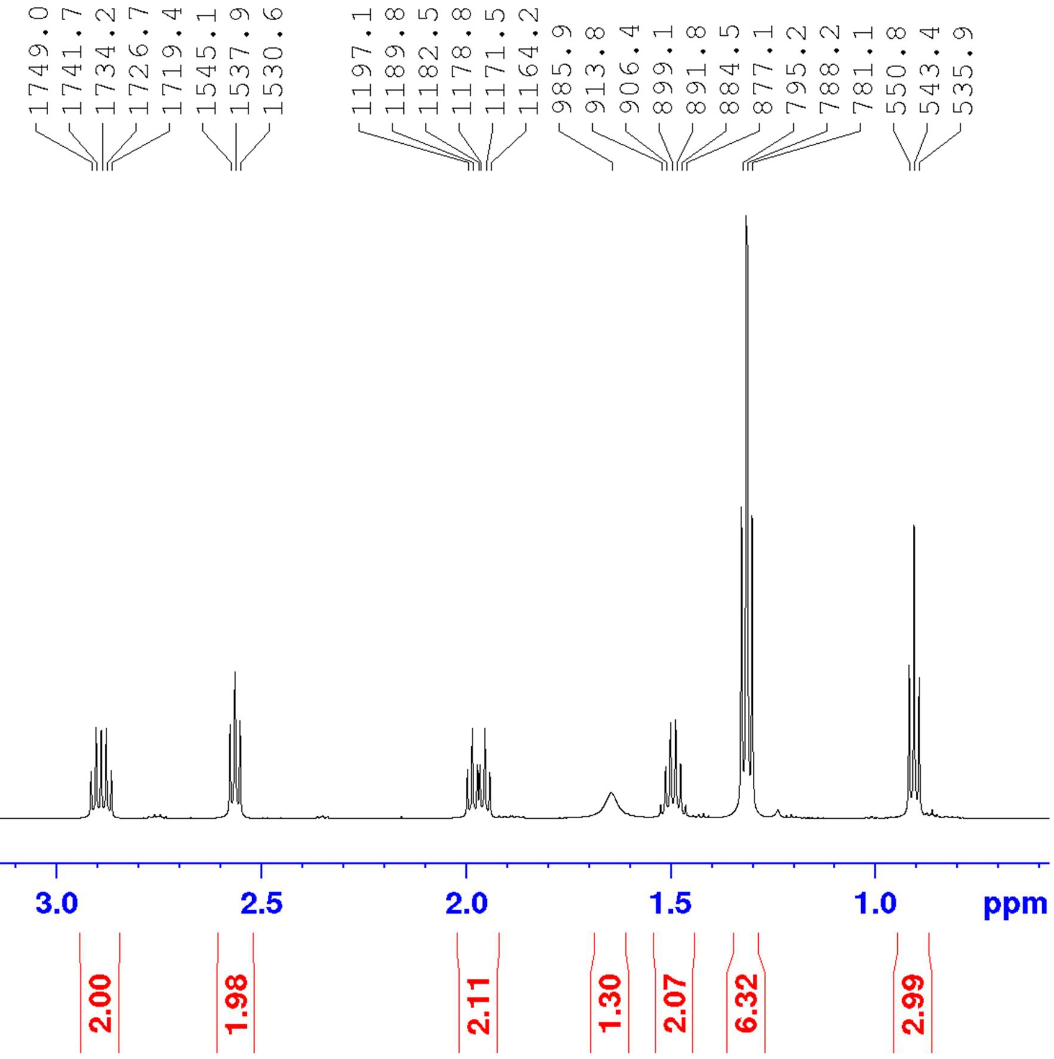

^{13}C NMR of Diethyl 2-propylamino-ethylphosphonate (**4**, partially as its hydrobromide)

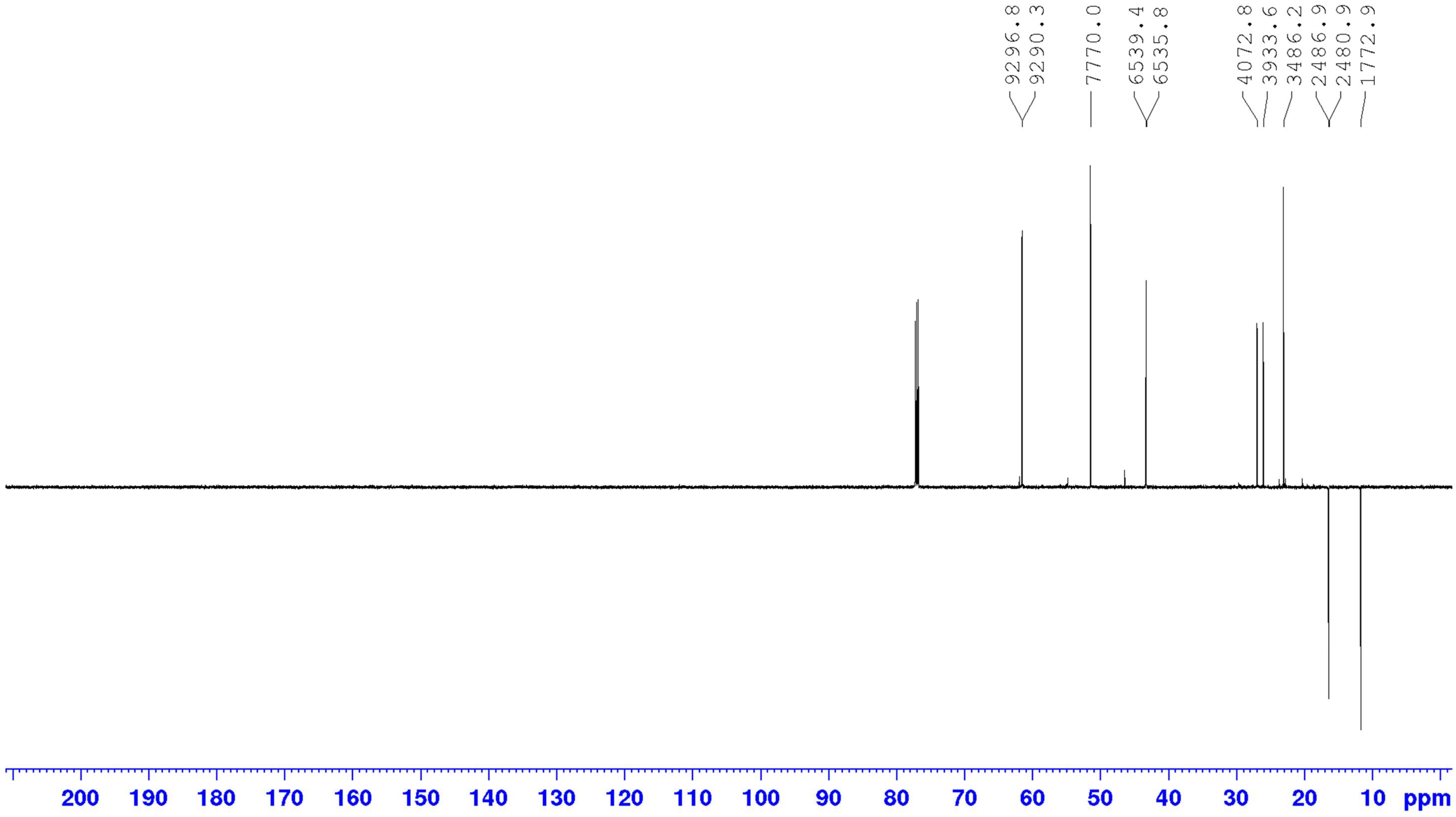

^{31}**P NMR of Diethyl 2-propylamino-ethylphosphonate** (**4**, partially as its hydrobromide)

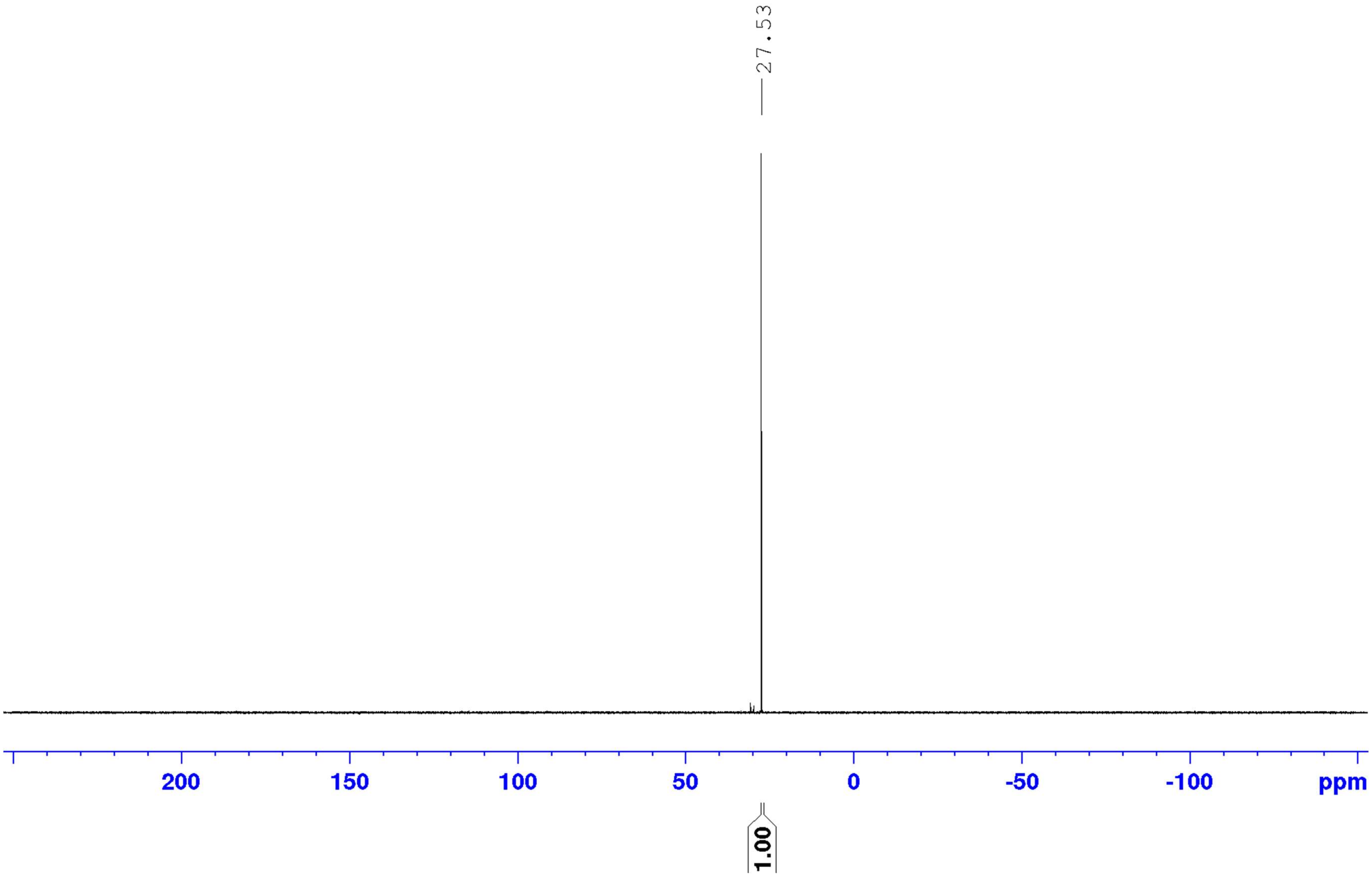

^{1}H NMR of 2-Propylamino-ethylphosphonic acid (P$_1$-AEP, 7)

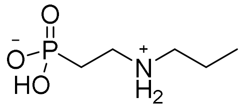

P$_1$-AEP (7)

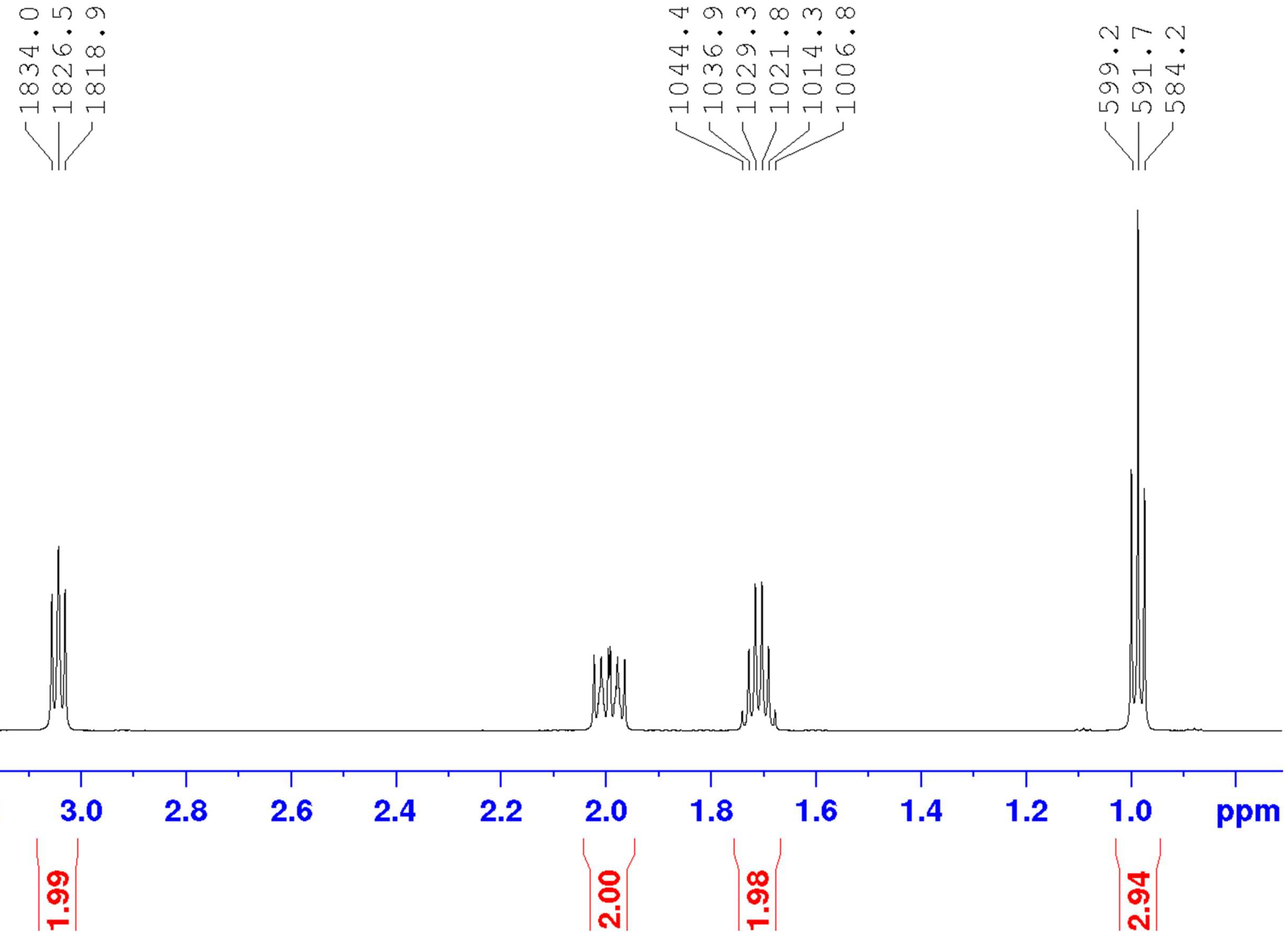

^{13}C NMR of 2-Propylamino-ethylphosphonic acid (P$_1$-AEP, 7)

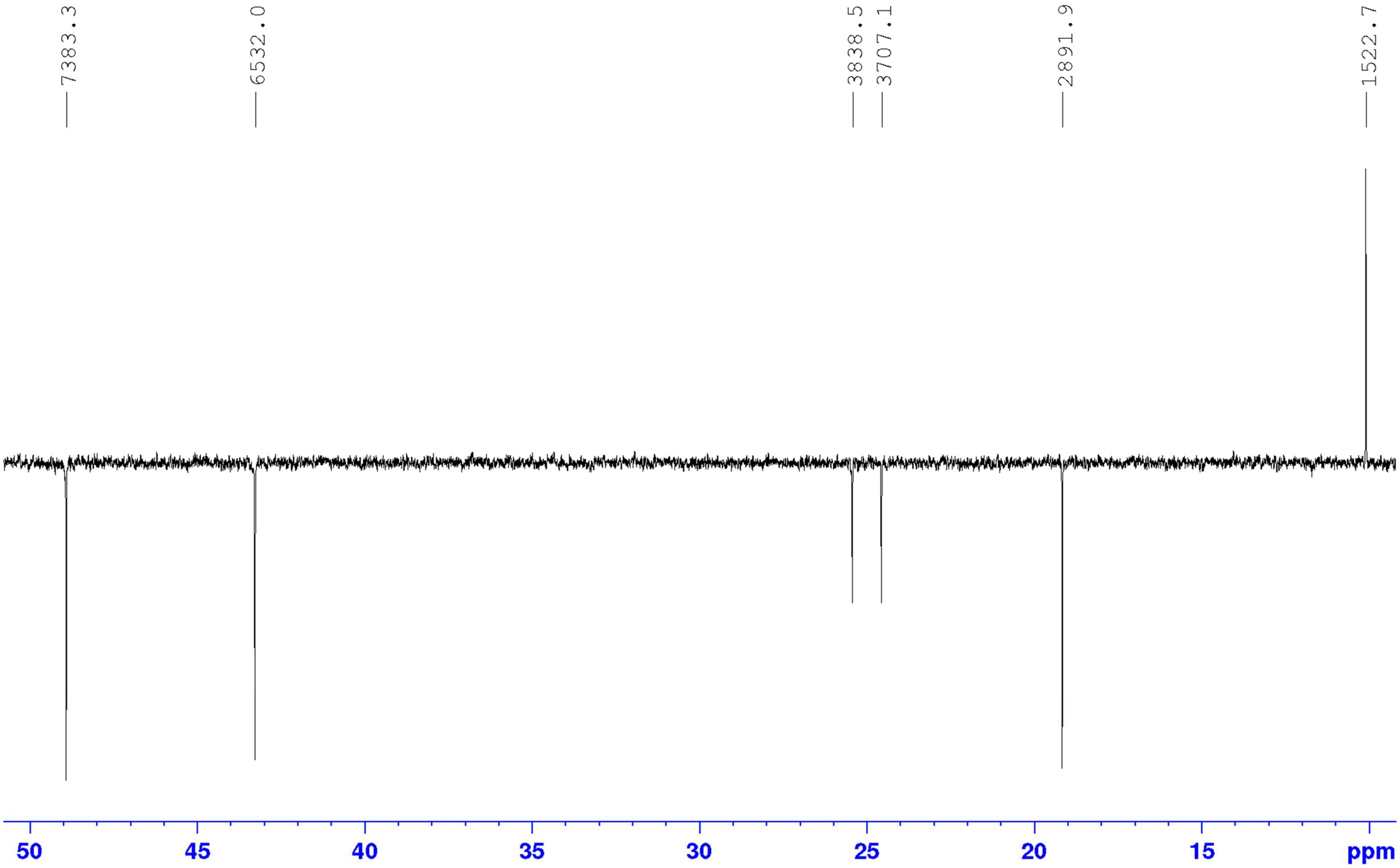

^{31}P NMR of 2-Propylamino-ethylphosphonic acid (P$_1$-AEP, 7)

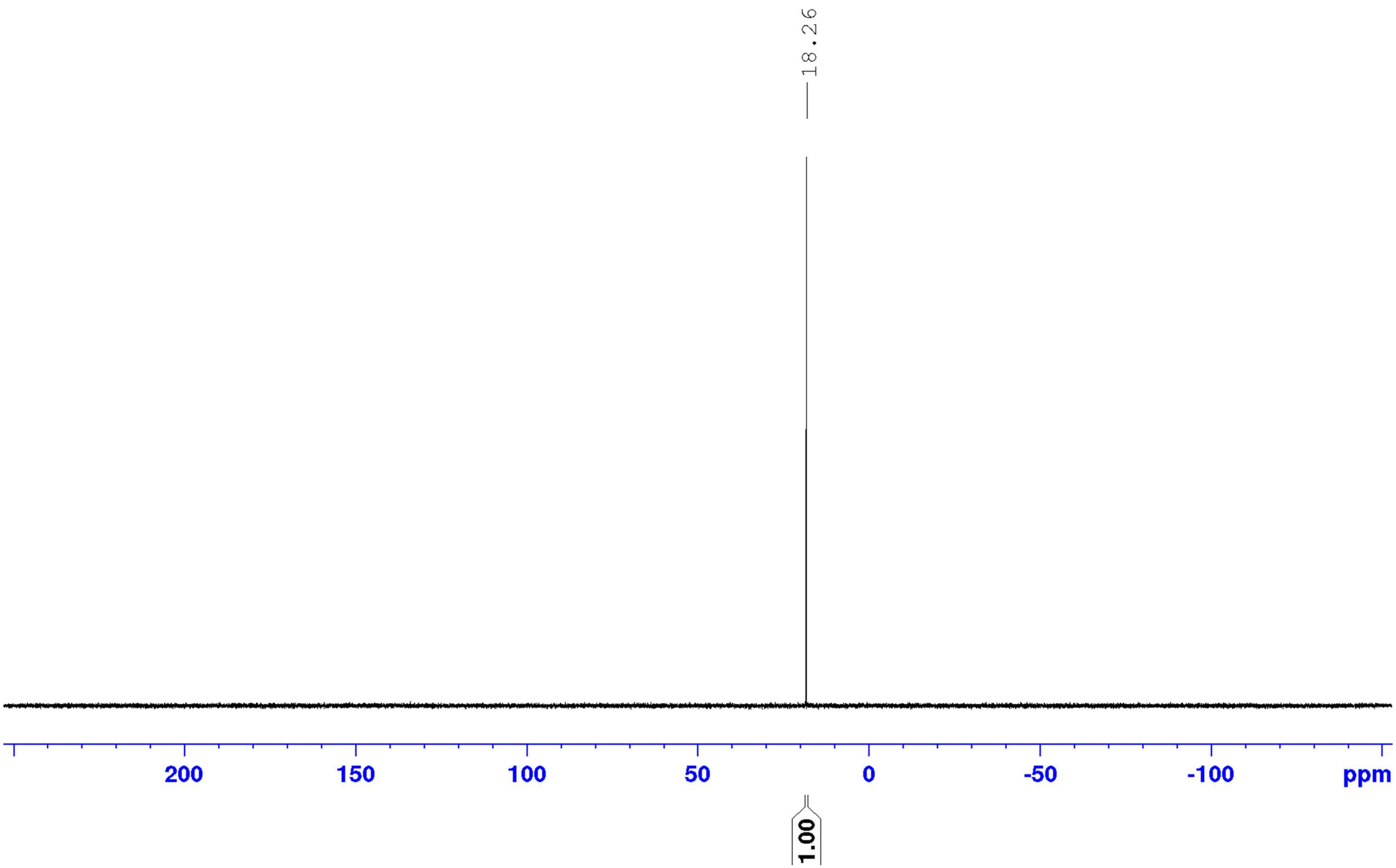

^{1}H NMR of Diethyl 2-dimethylamino-ethylphosphonate (**8**, partially as hydrobromide)

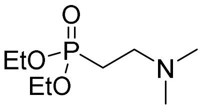

8

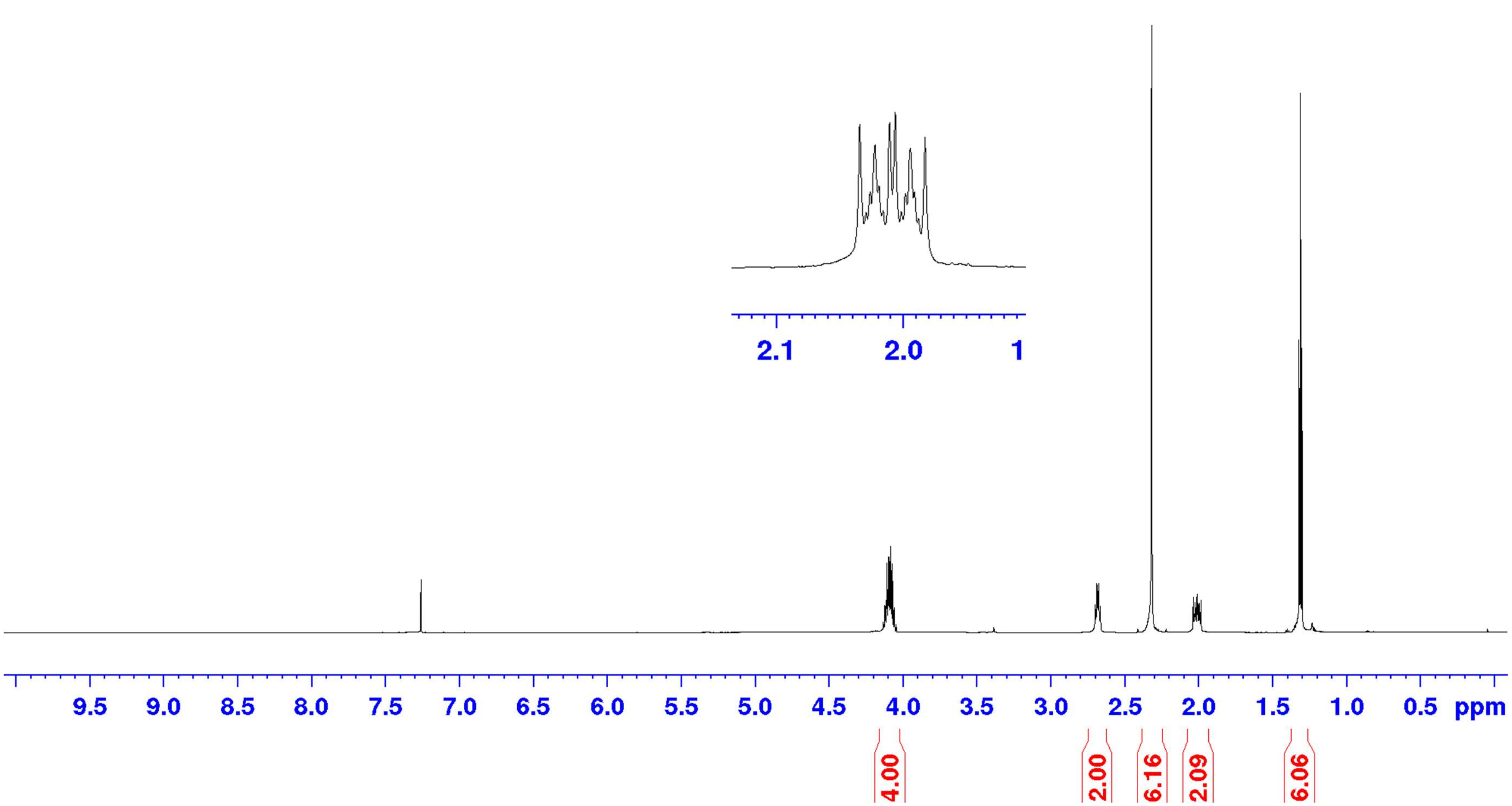

^{13}**C NMR of Diethyl 2-dimethylamino-ethylphosphonate** (**8**, partially as hydrobromide)

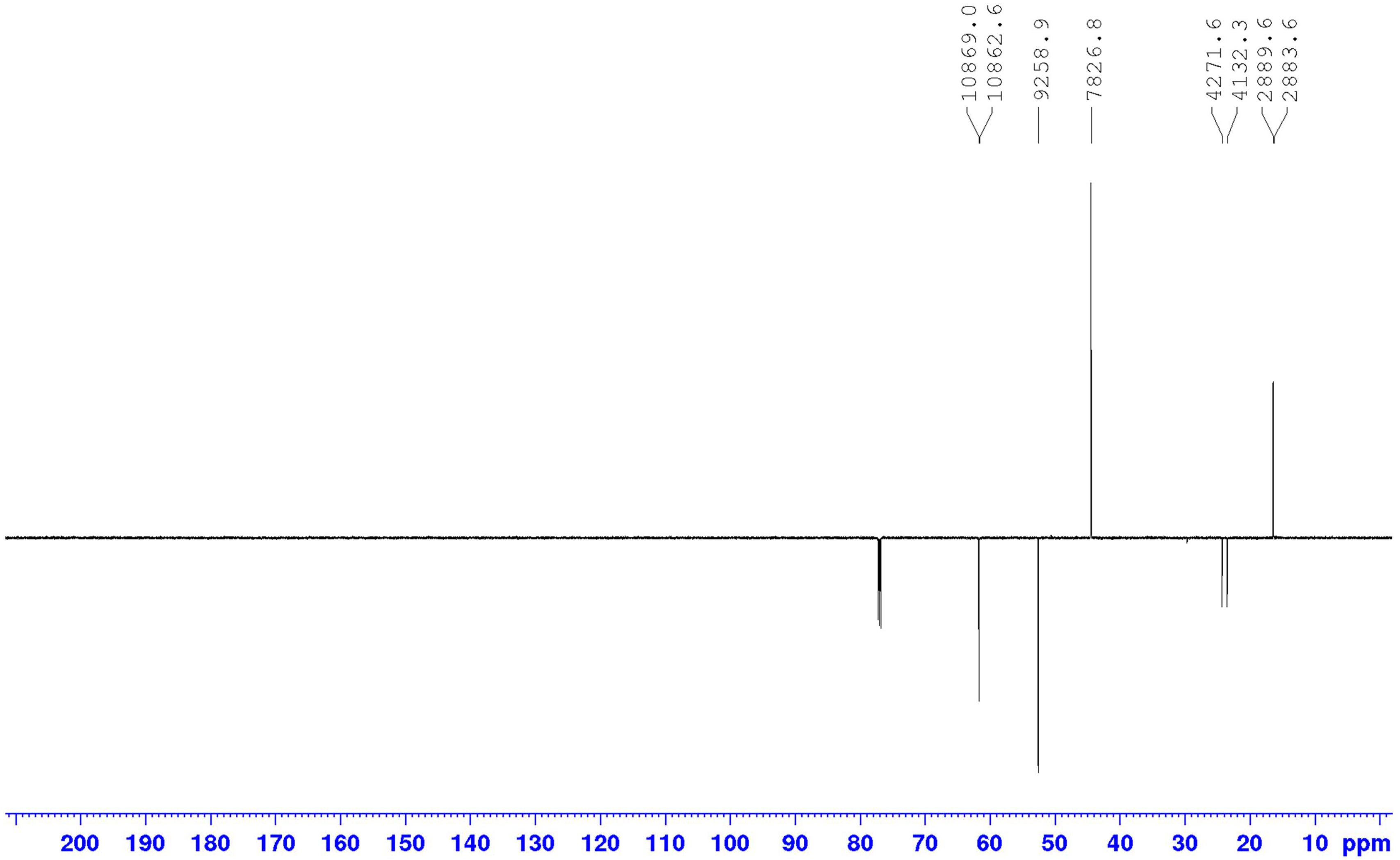

^{31}**P NMR of Diethyl 2-dimethylamino-ethylphosphonate** (**8**, partially as hydrobromide)

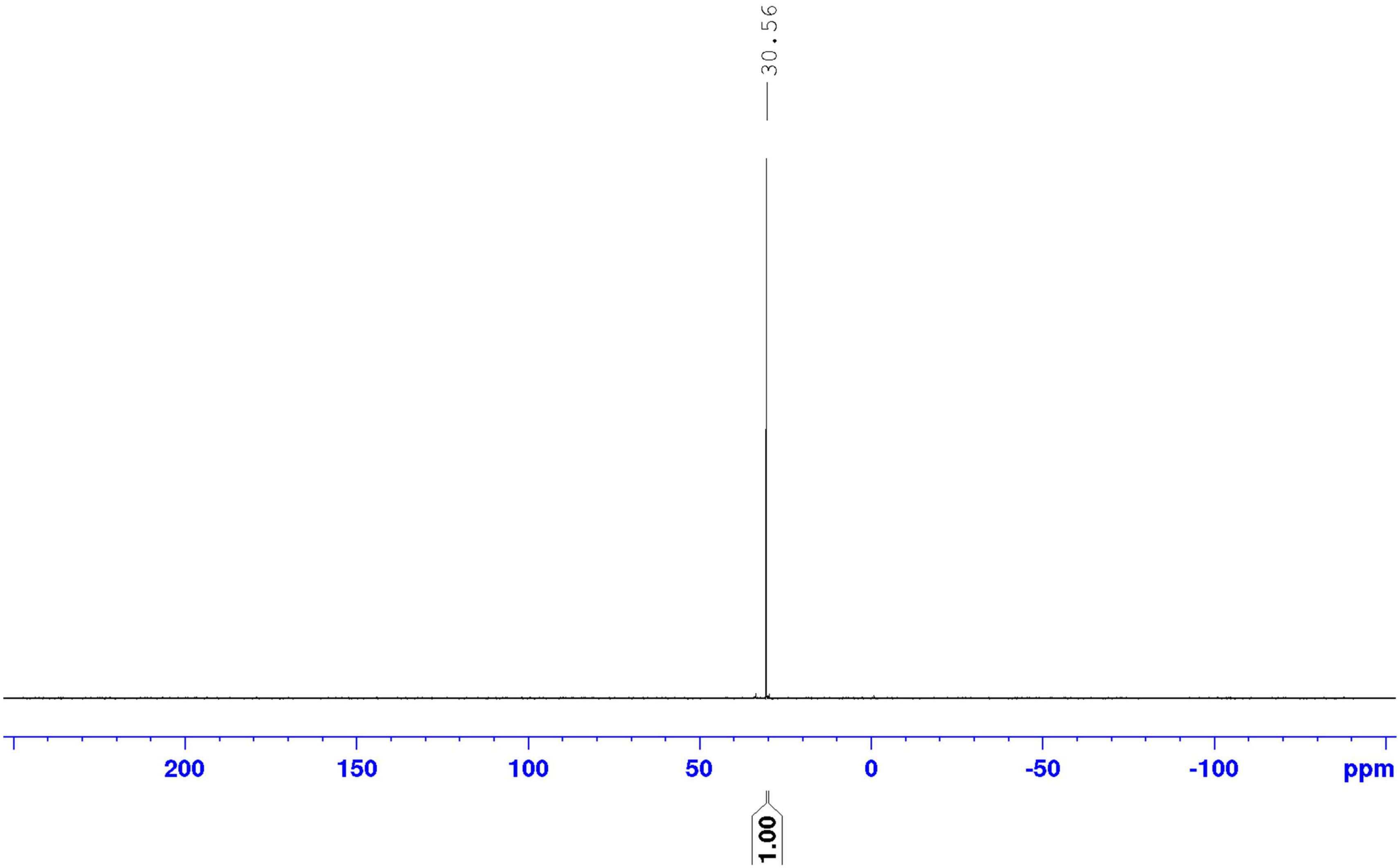

^{1}H NMR of 2-Dimethylamino-ethylphosphonic acid (M$_2$-AEP, 9)

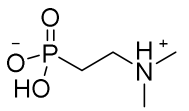

M$_2$-AEP
9

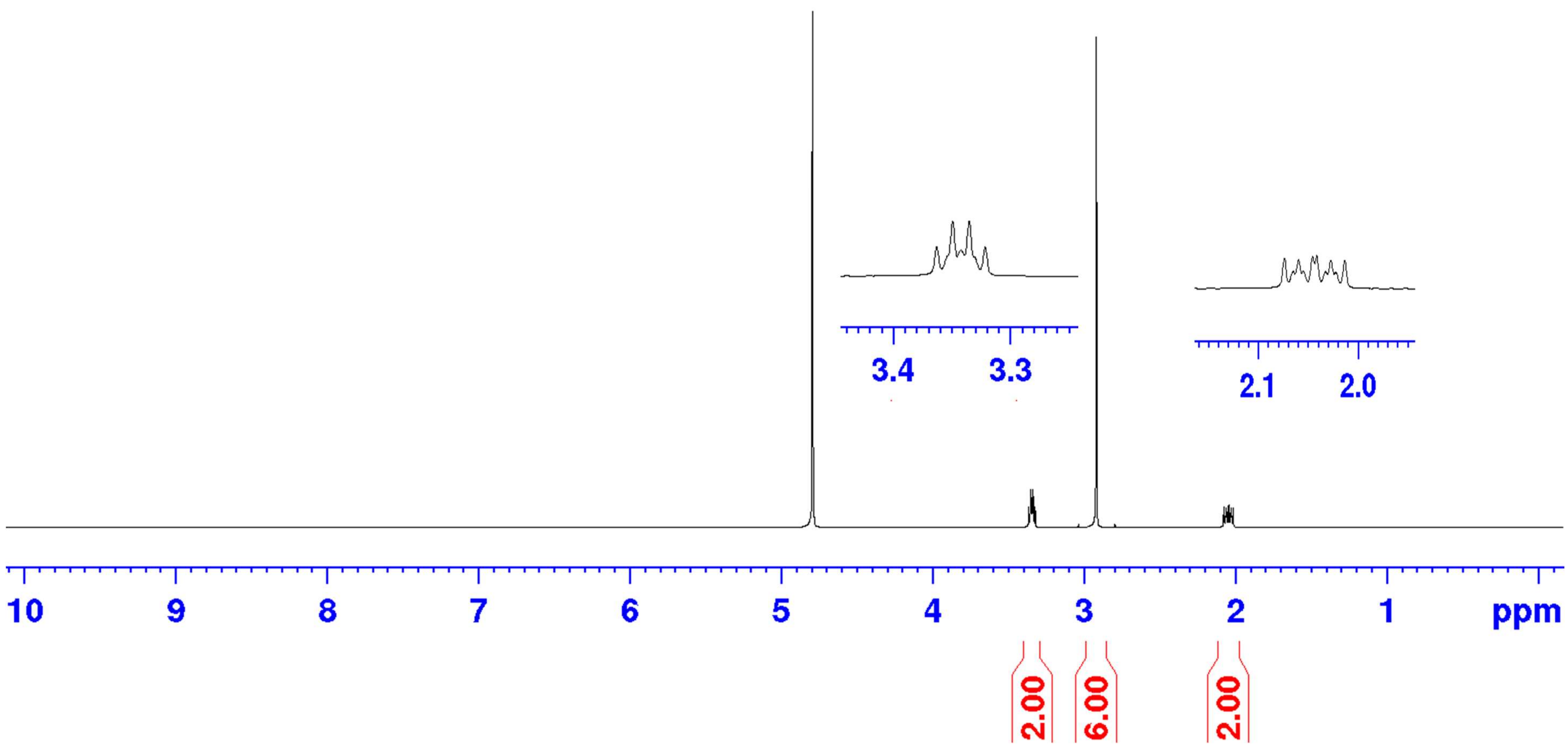

^{13}C NMR of 2-Dimethylamino-ethylphosphonic acid (**M_2-AEP**, **9**)

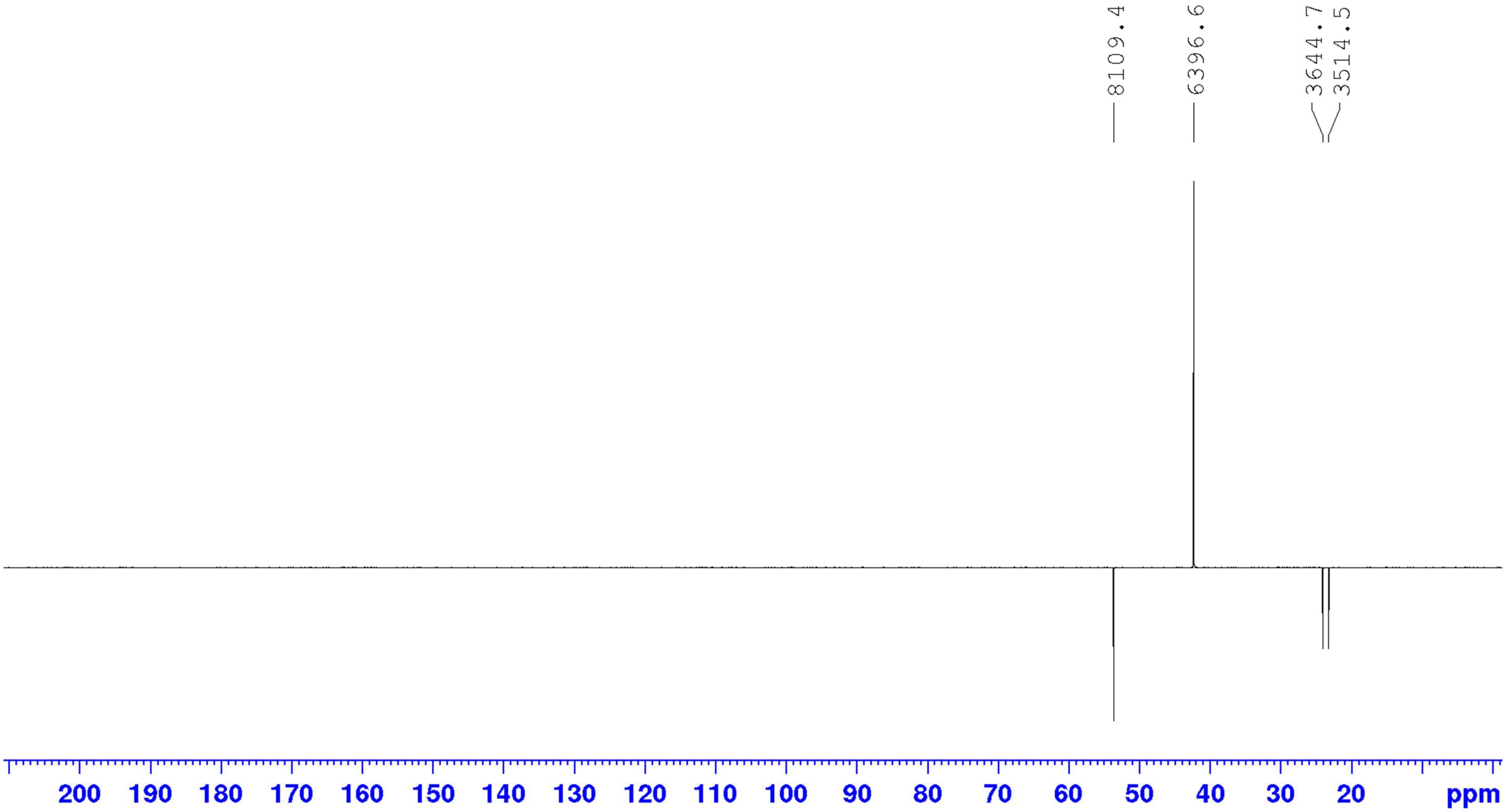

^{31}P NMR of 2-Dimethylamino-ethylphosphonic acid (**M_2-AEP**, **9**)

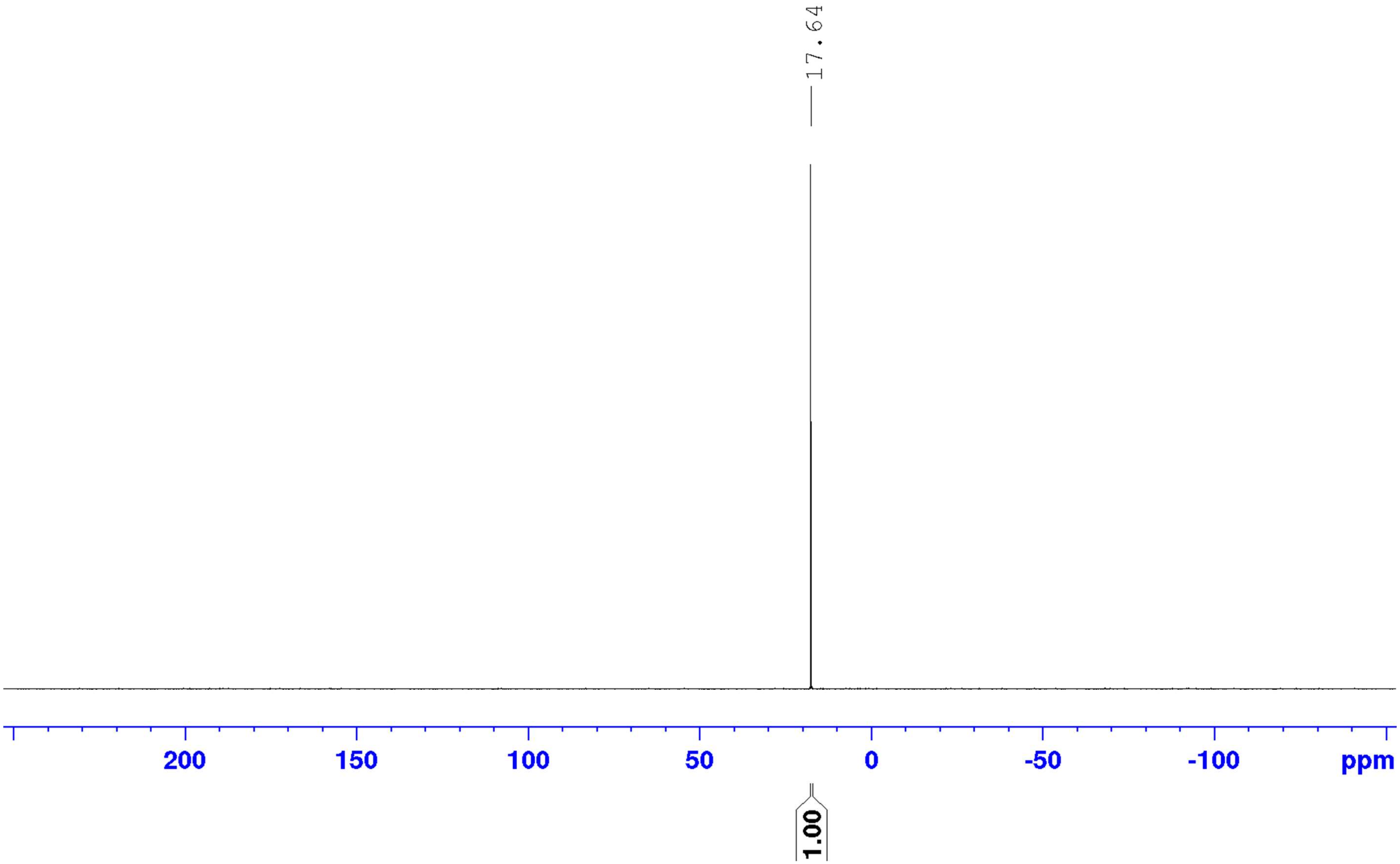

^{1}H NMR of Diethyl 2-trimethylammonium-ethylphosphonate iodide (10)

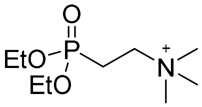

10 as its iodide

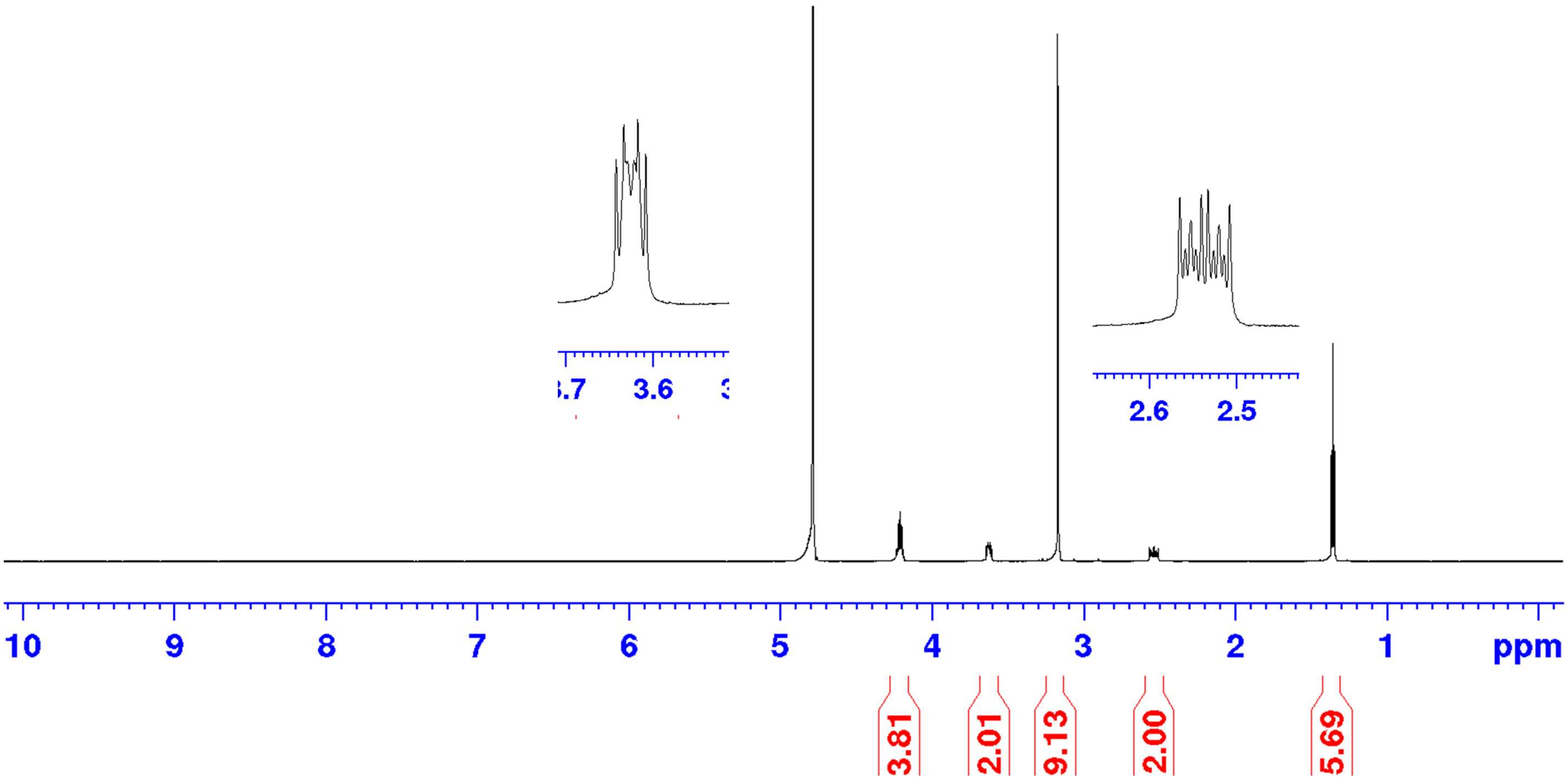

^{13}C NMR of Diethyl 2-trimethylammonium-ethylphosphonate iodide (10)

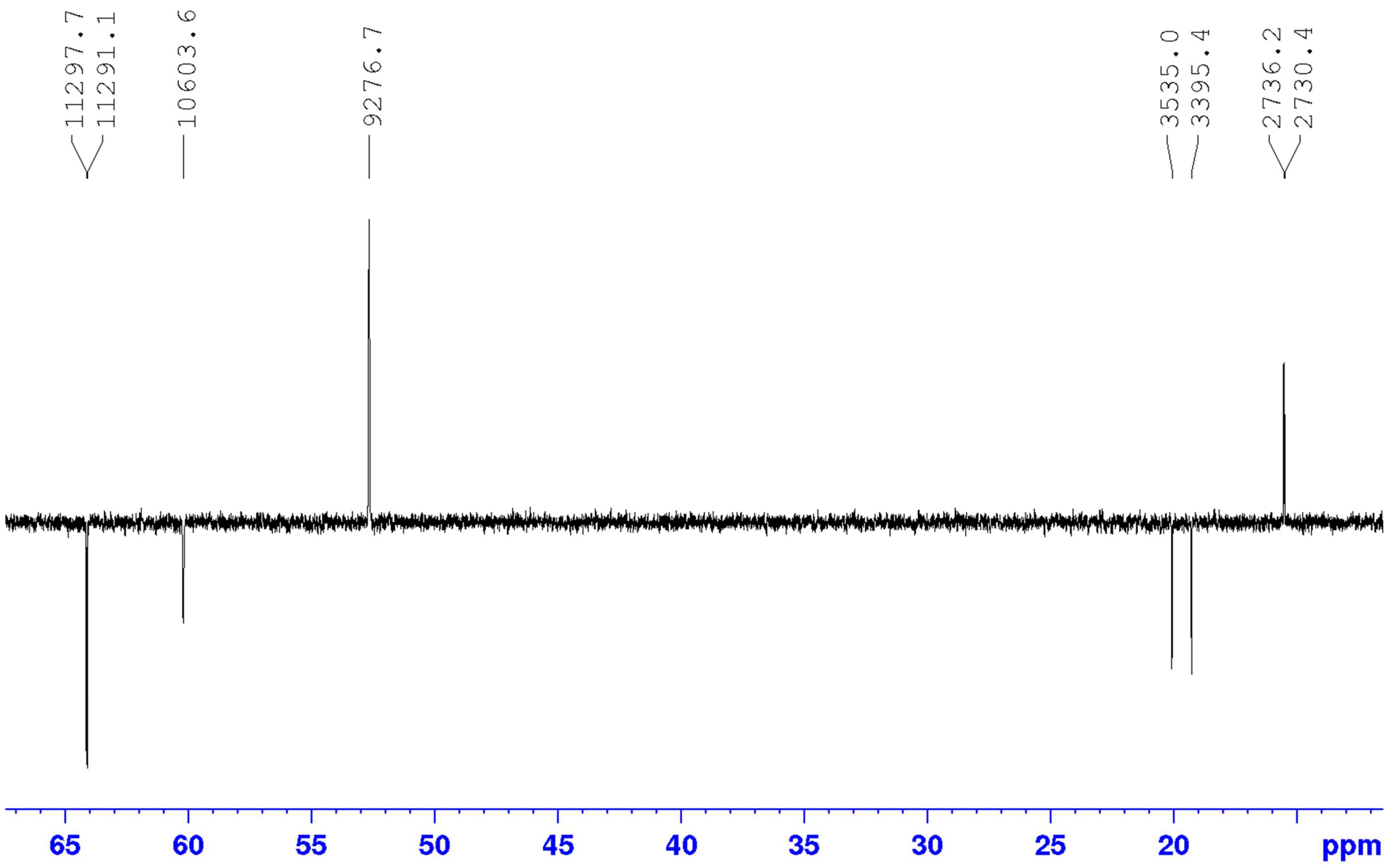

^{31}P NMR of Diethyl 2-trimethylammonium-ethylphosphonate iodide (10))

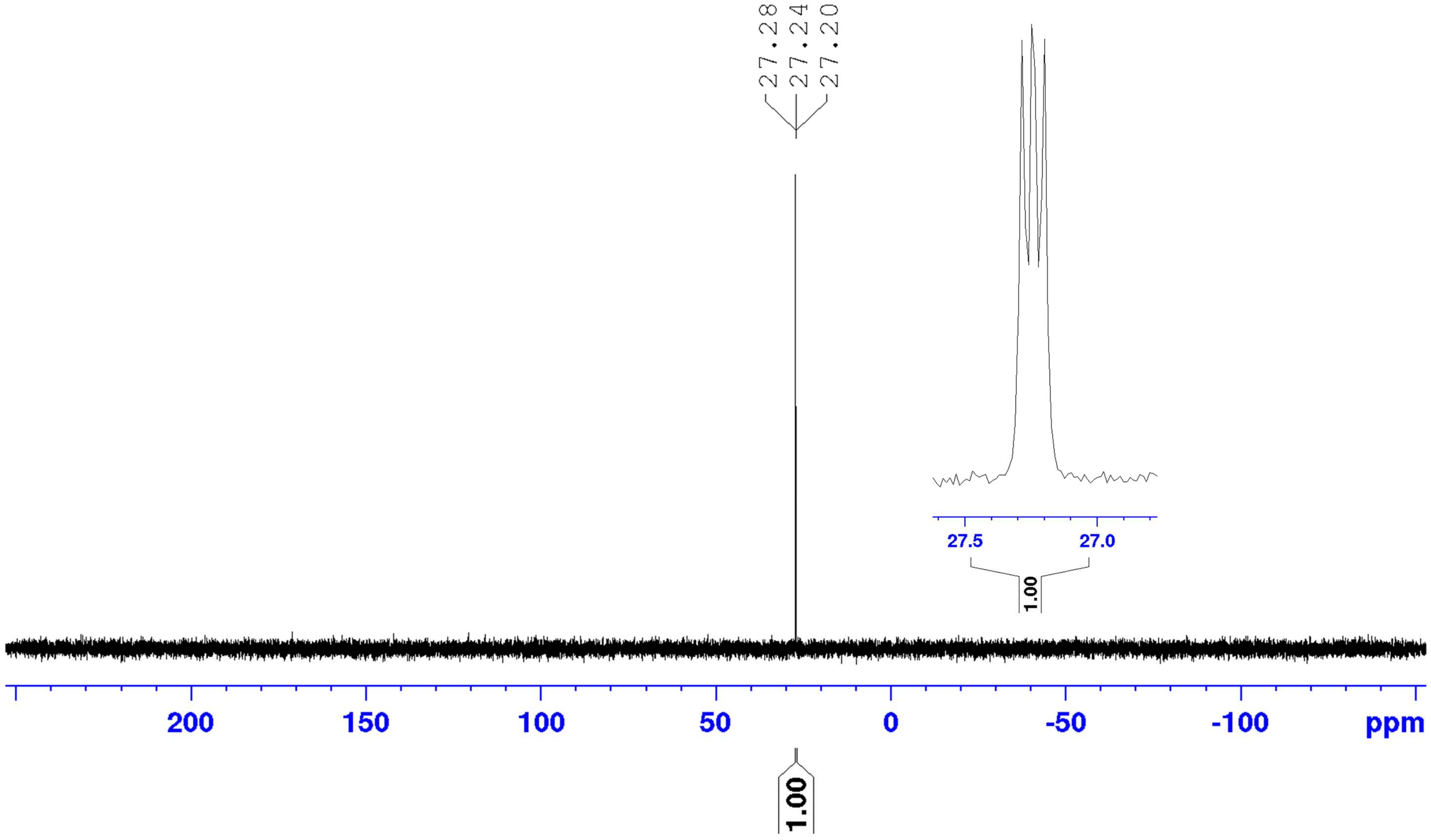

^{1}H NMR of 2-Trimethylammonio-ethylphosphonic acid (M$_3$-AEP, 11)

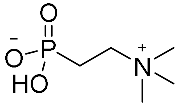

M_3-AEP
11

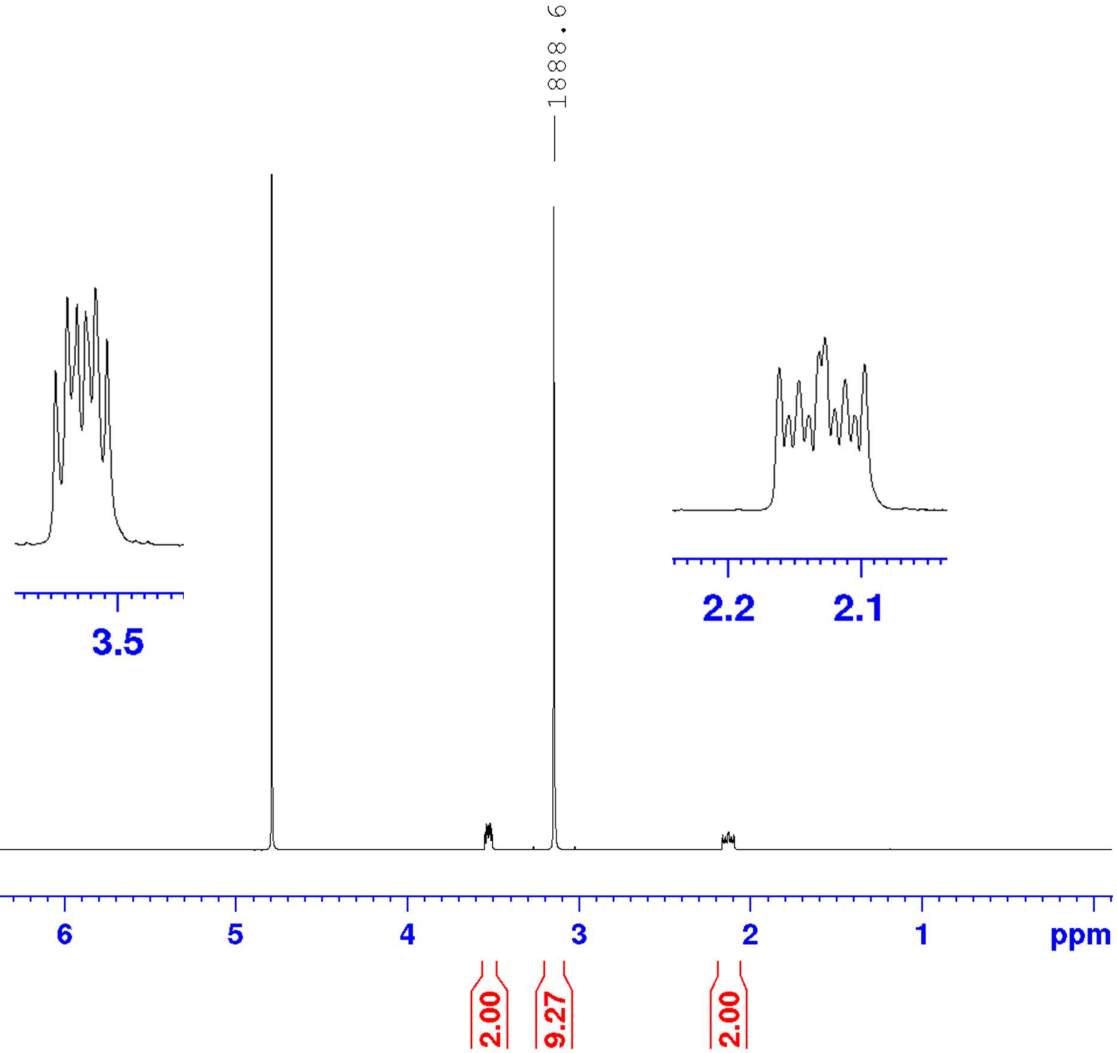

^{13}C NMR of 2-Trimethylammonio-ethylphosphonic acid (M$_3$-AEP, 11)

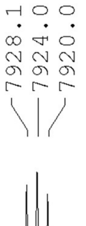

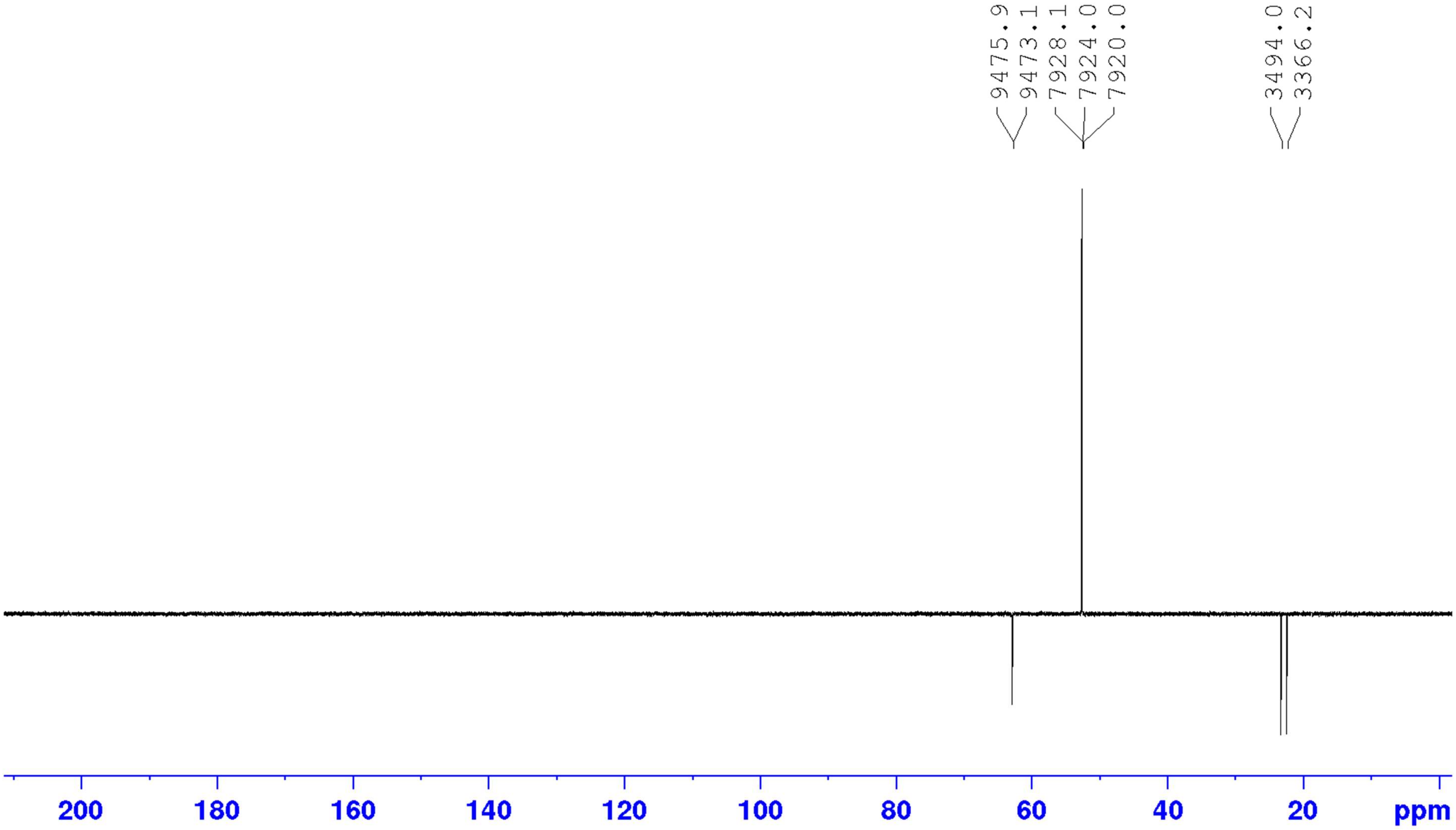
9475.9
9473.1
7928.1
7924.0
7920.0
3494.0
3366.2
200
180
160
140
120
100
80
60
40
20
ppm

^{31}P NMR of 2-Trimethylammonio-ethylphosphonic acid (M$_3$-AEP, 11)

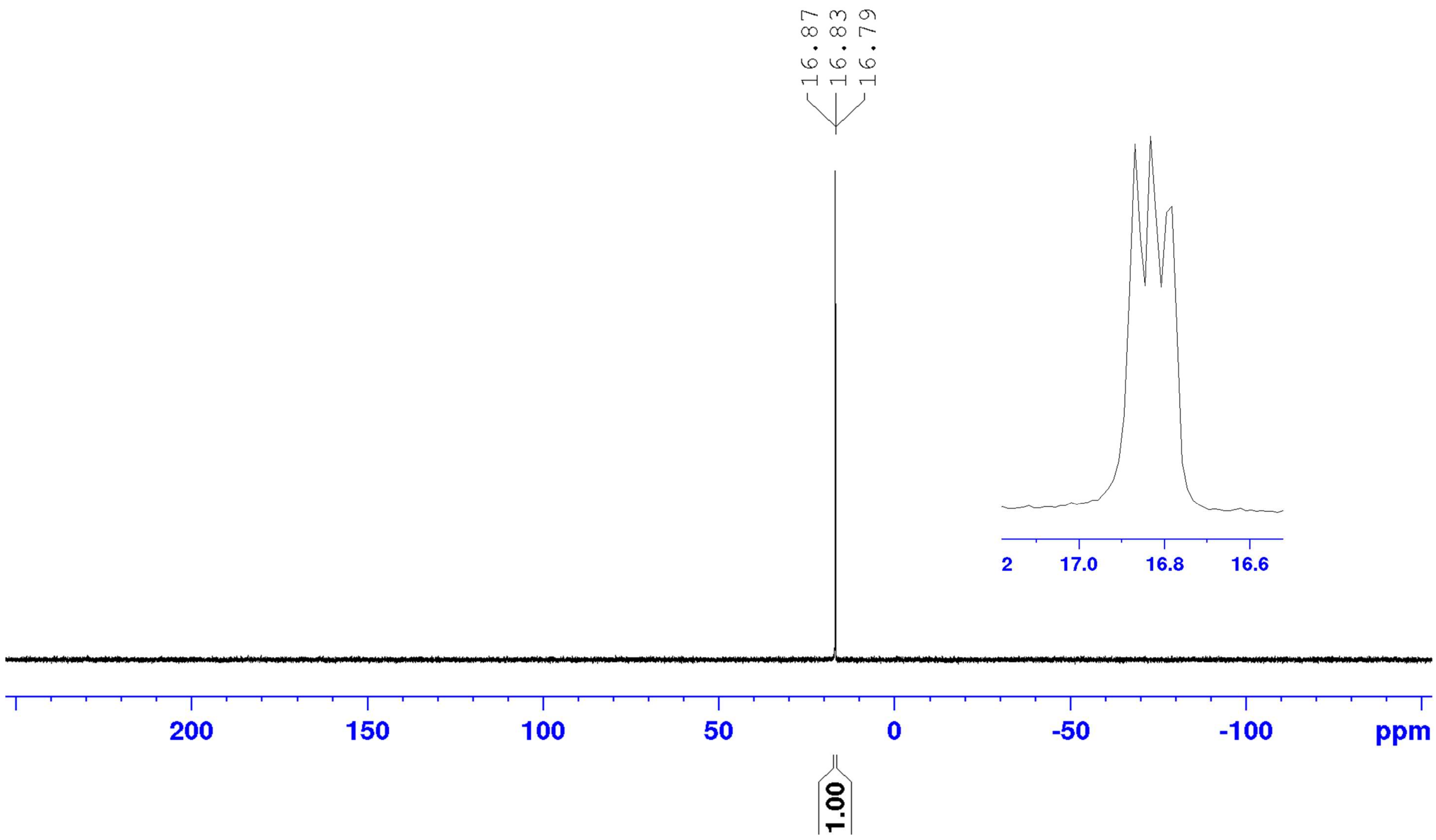

^{1}H NMR of (*R*)-Diisopropyl 1-hydroxy-2-aminoethylphosphonate [(*R*)-**14**]

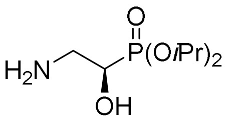

(*R*)-**14**

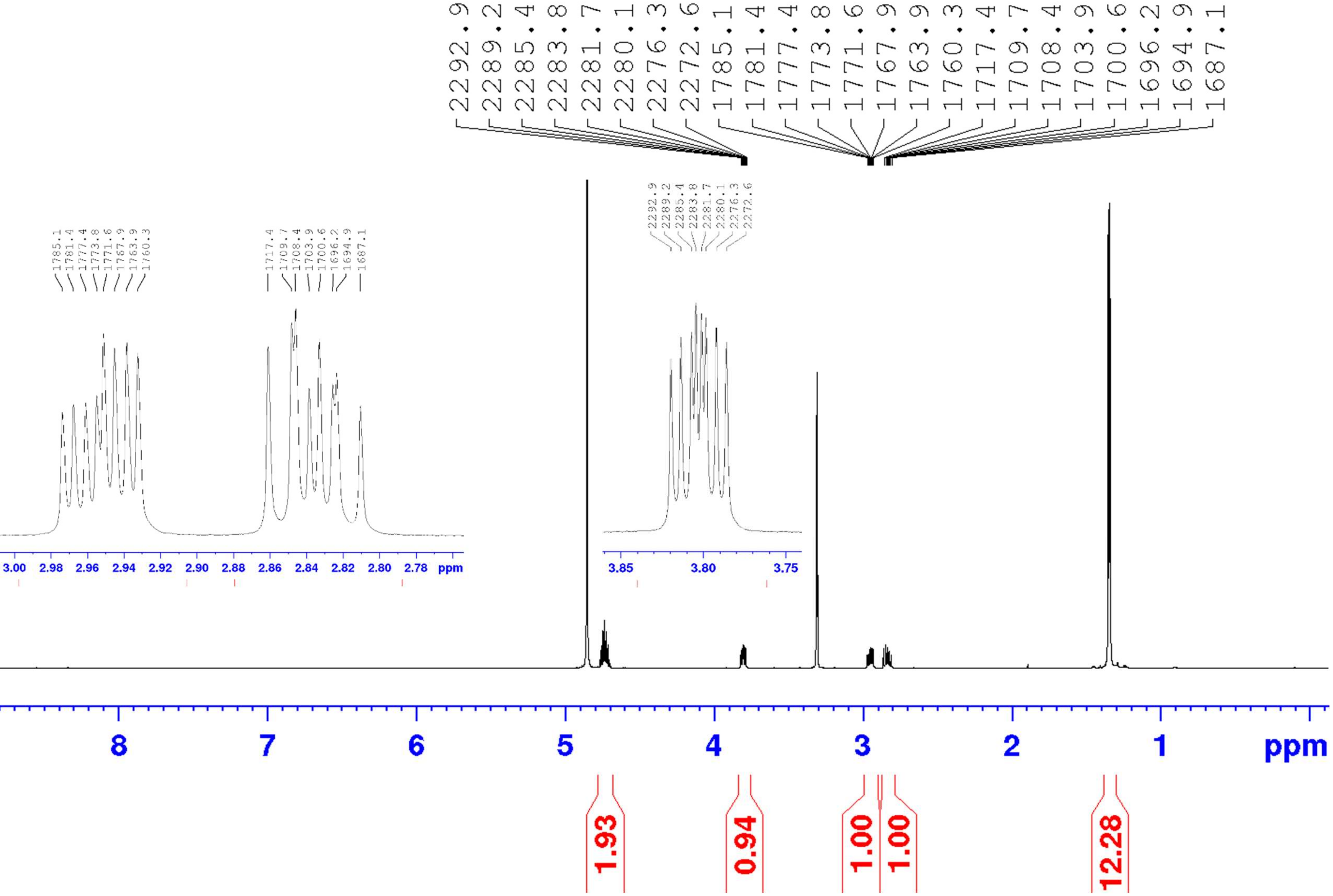

^{13}C NMR of (*R*)-Diisopropyl 1-hydroxy-2-aminoethylphosphonate [(*R*)-**14**]

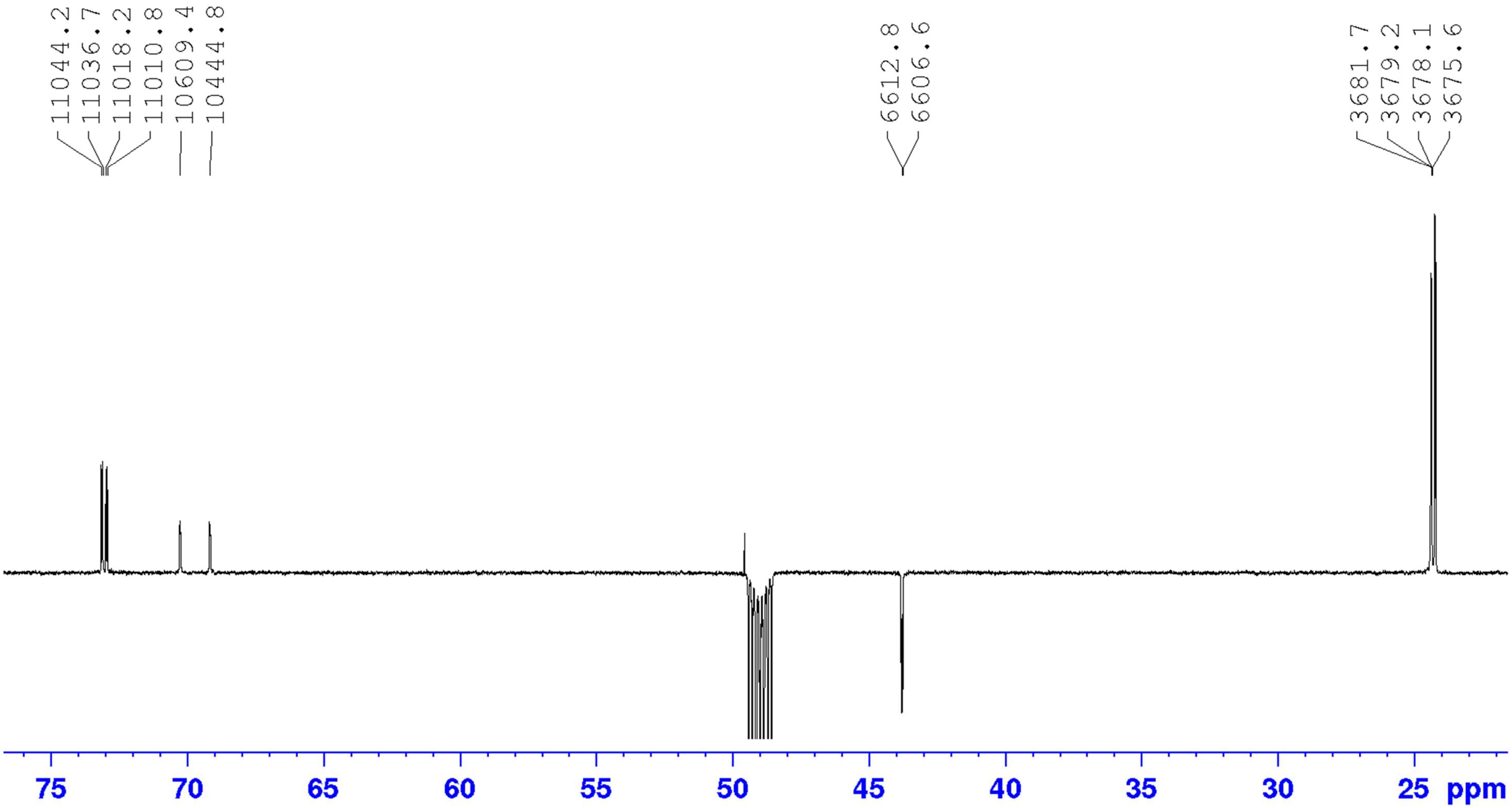

^{31}P NMR of (*R*)-Diisopropyl 1-hydroxy-2-aminoethylphosphonate [(*R*)-**14**]

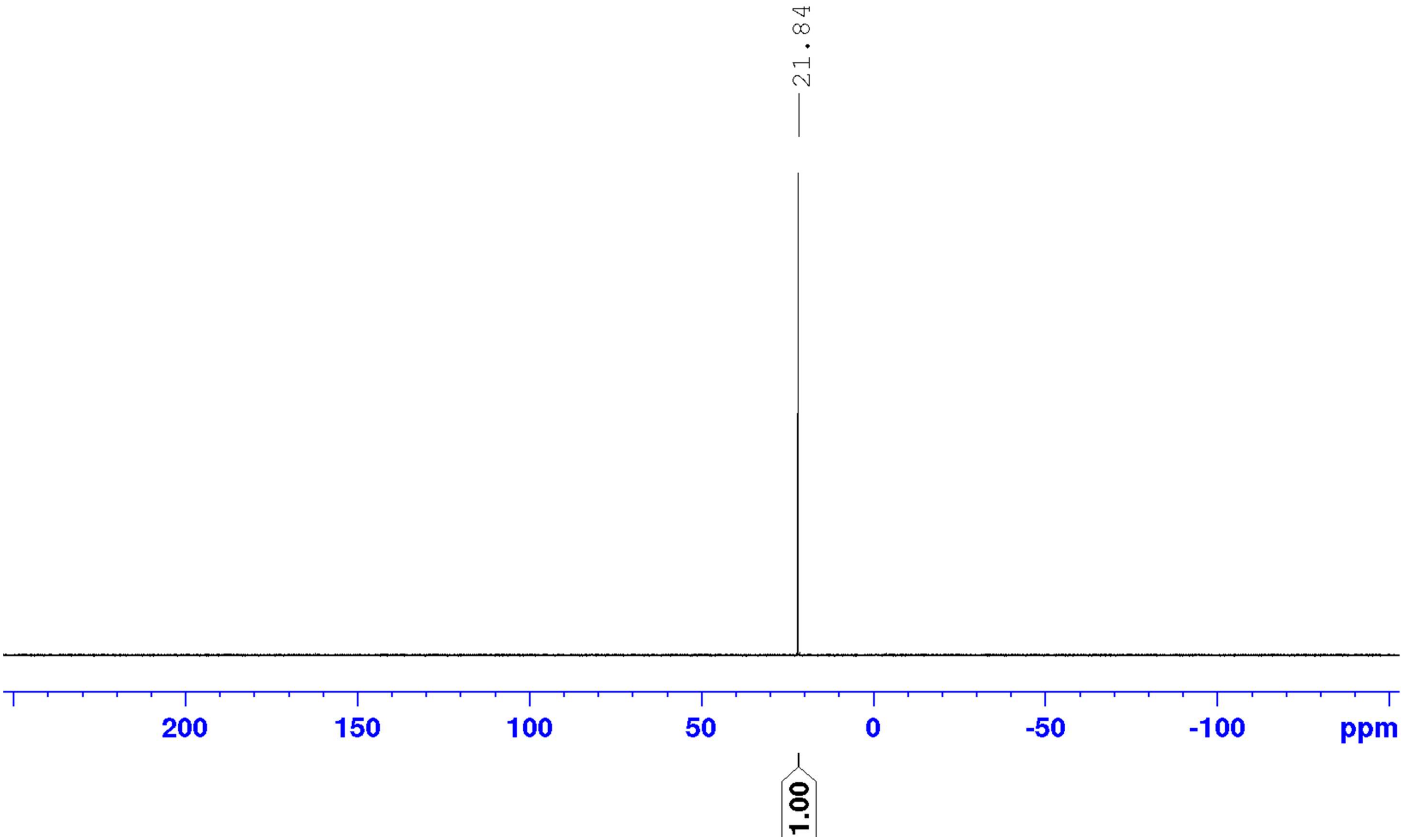

^{1}H NMR of (*R*)-Diisopropyl 1-hydroxy-2-tosylamido-ethylphosphonate [(*R*)-**15**]

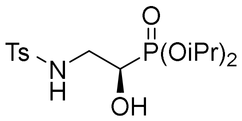

(*R*)-**15**

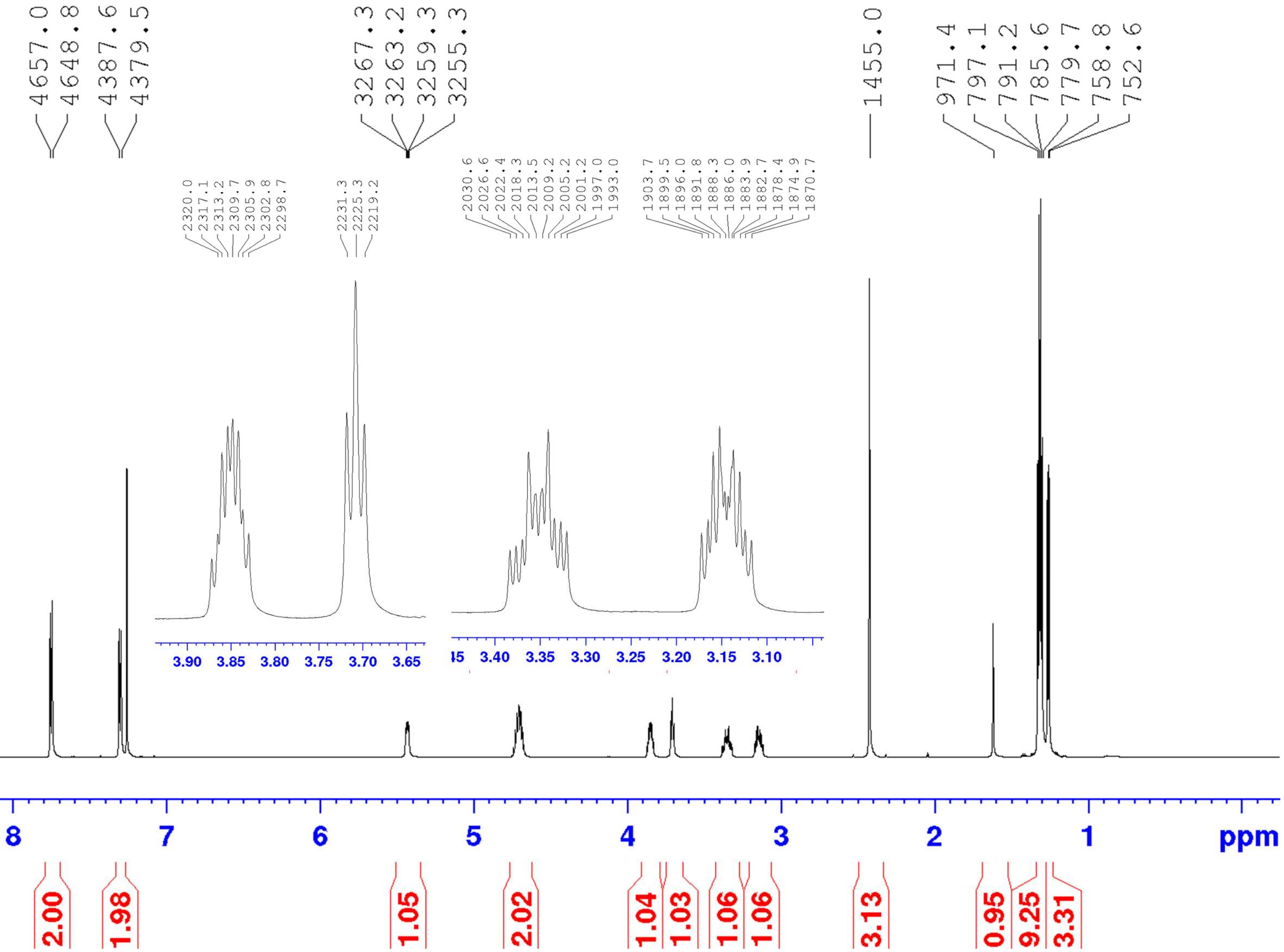

^{13}C NMR of (*R*)-Diisopropyl 1-hydroxy-2-tosylamido-ethylphosphonate [(*R*)-**15**]

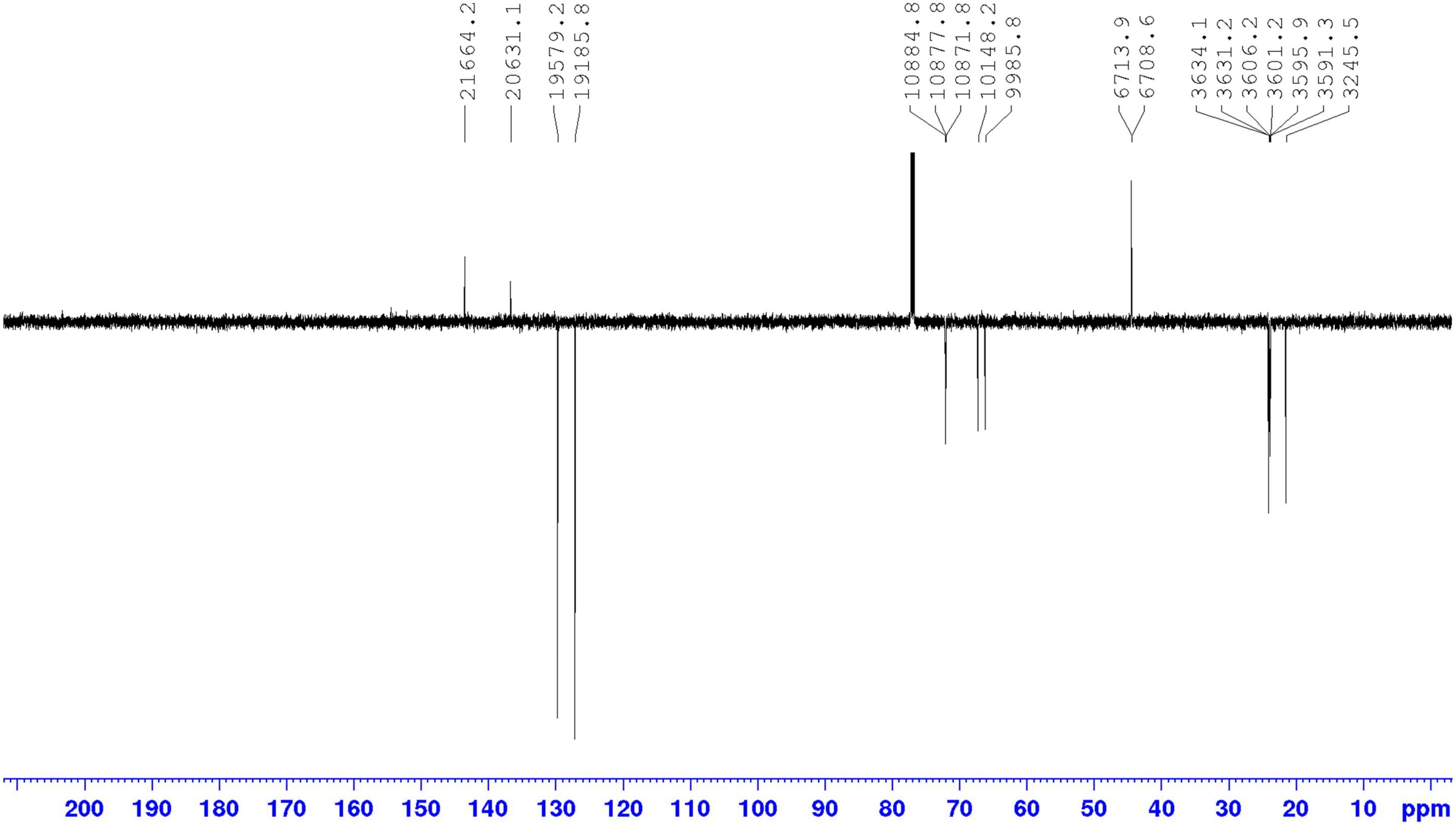

^{31}P NMR of (*R*)-Diisopropyl 1-hydroxy-2-tosylamido-ethylphosphonate [(*R*)-**15**]

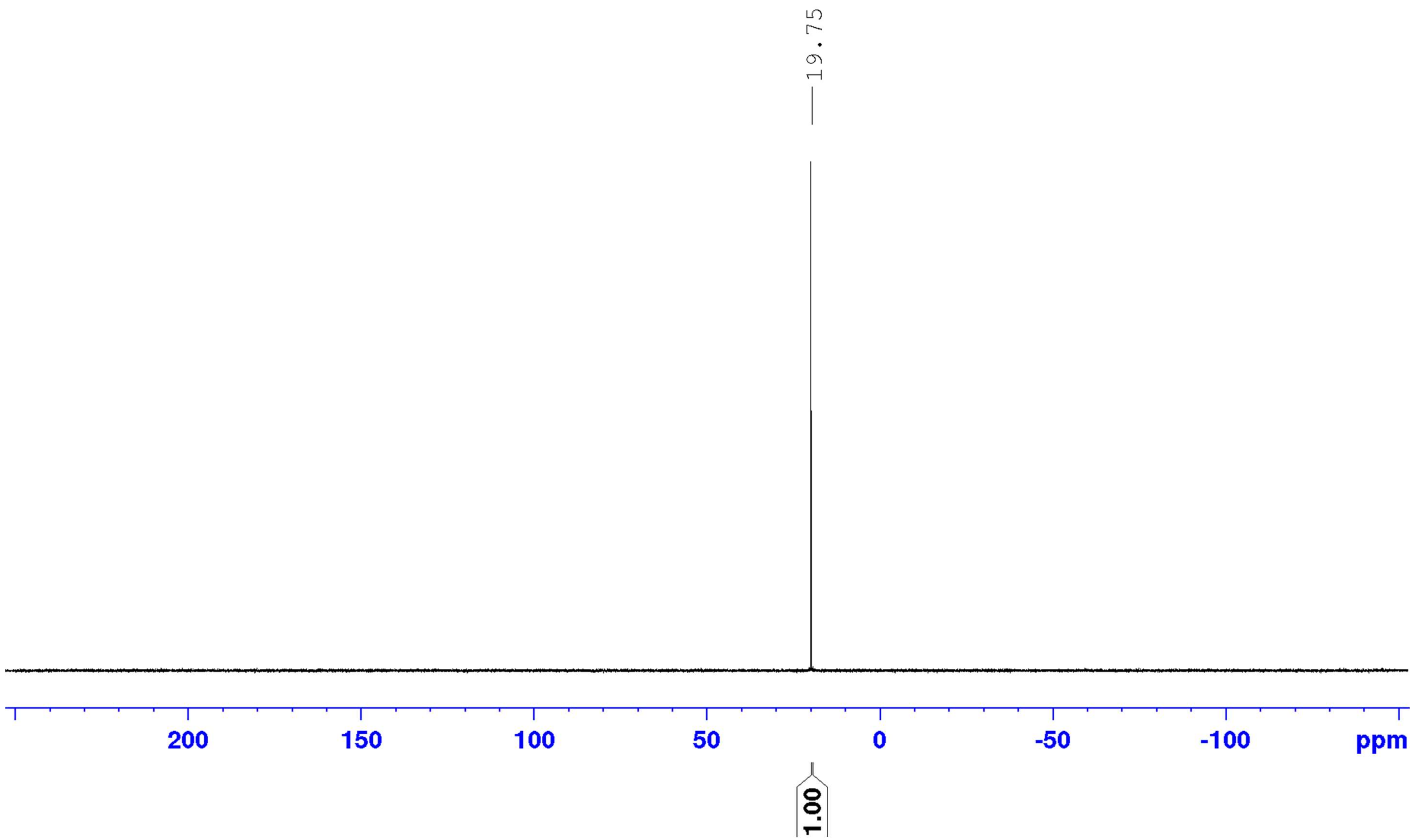

^{1}H NMR of (*R*)-Diisopropyl 1-hydroxy-2-(*N*-methyl-*N*-tosyl-amido)-ethylphosphonate [(*R*)-**16**]

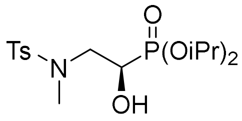

(*R*)-**16**

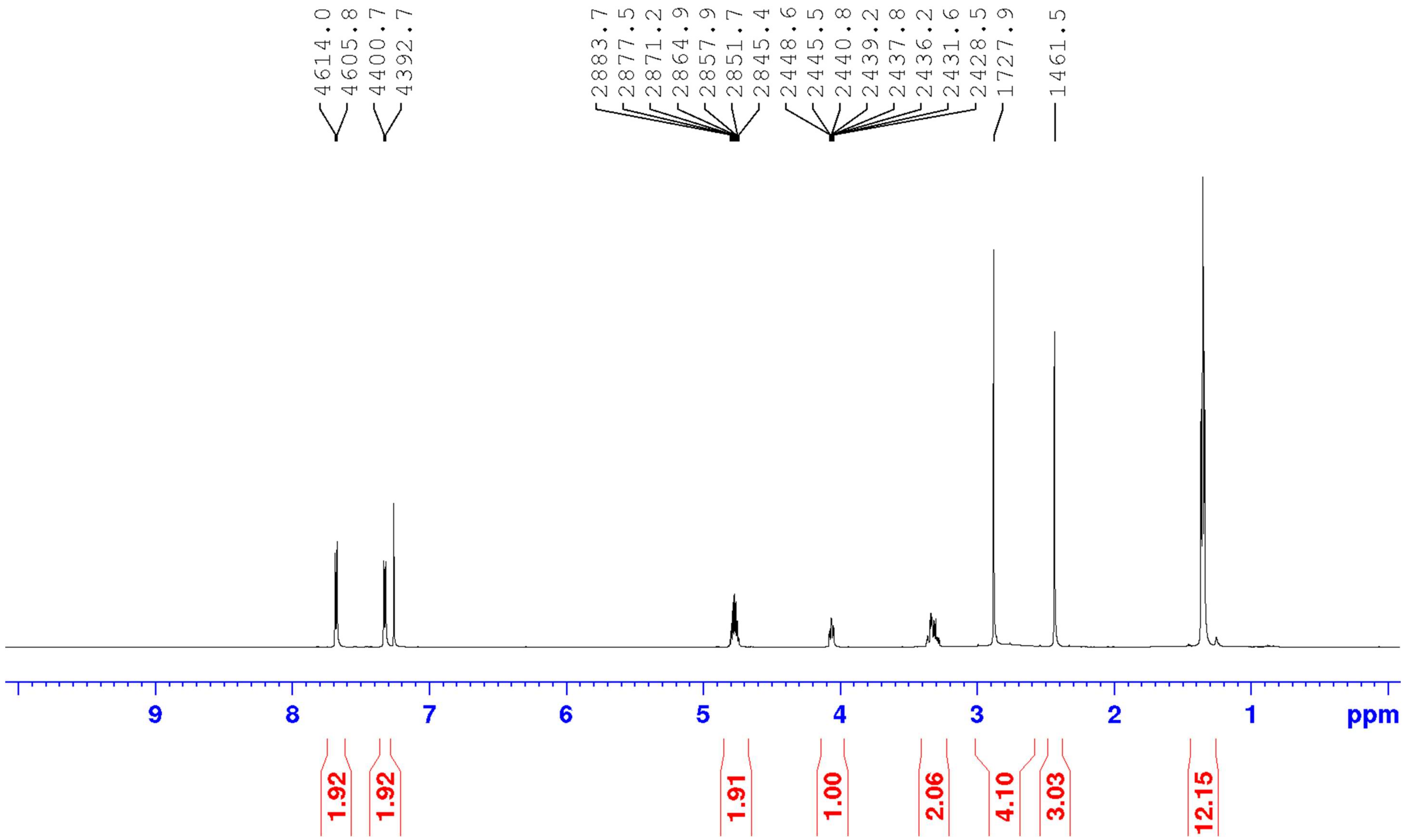

^{13}C NMR of (*R*)-Diisopropyl 1-hydroxy-2-(*N*-methyl-*N*-tosyl-amido)-ethylphosphonate [(*R*)-**16**]

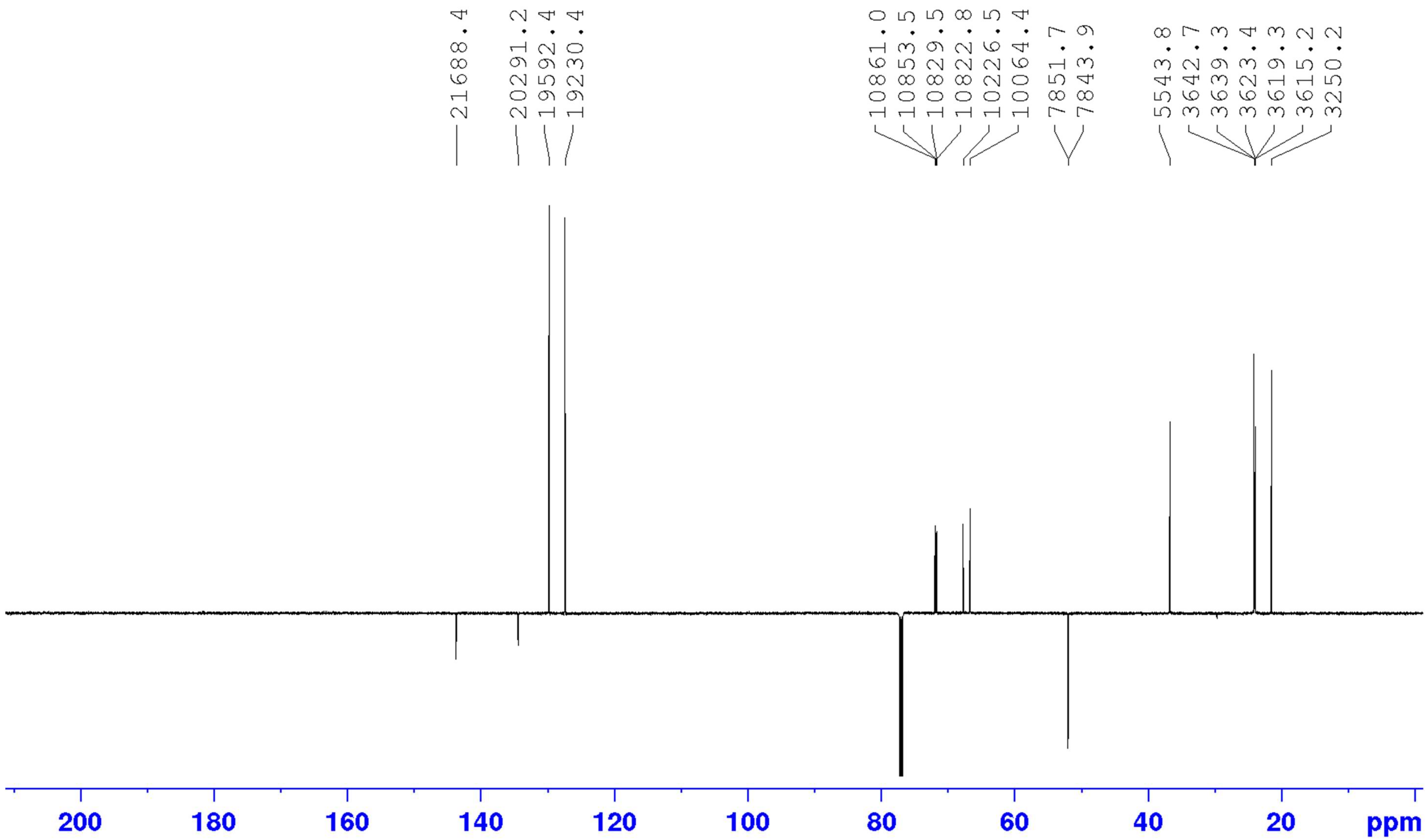

^{31}P NMR of ((*R*)-Diisopropyl 1-hydroxy-2-(*N*-methyl-*N*-tosyl-amido)-ethylphosphonate [(*R*)-**16**]

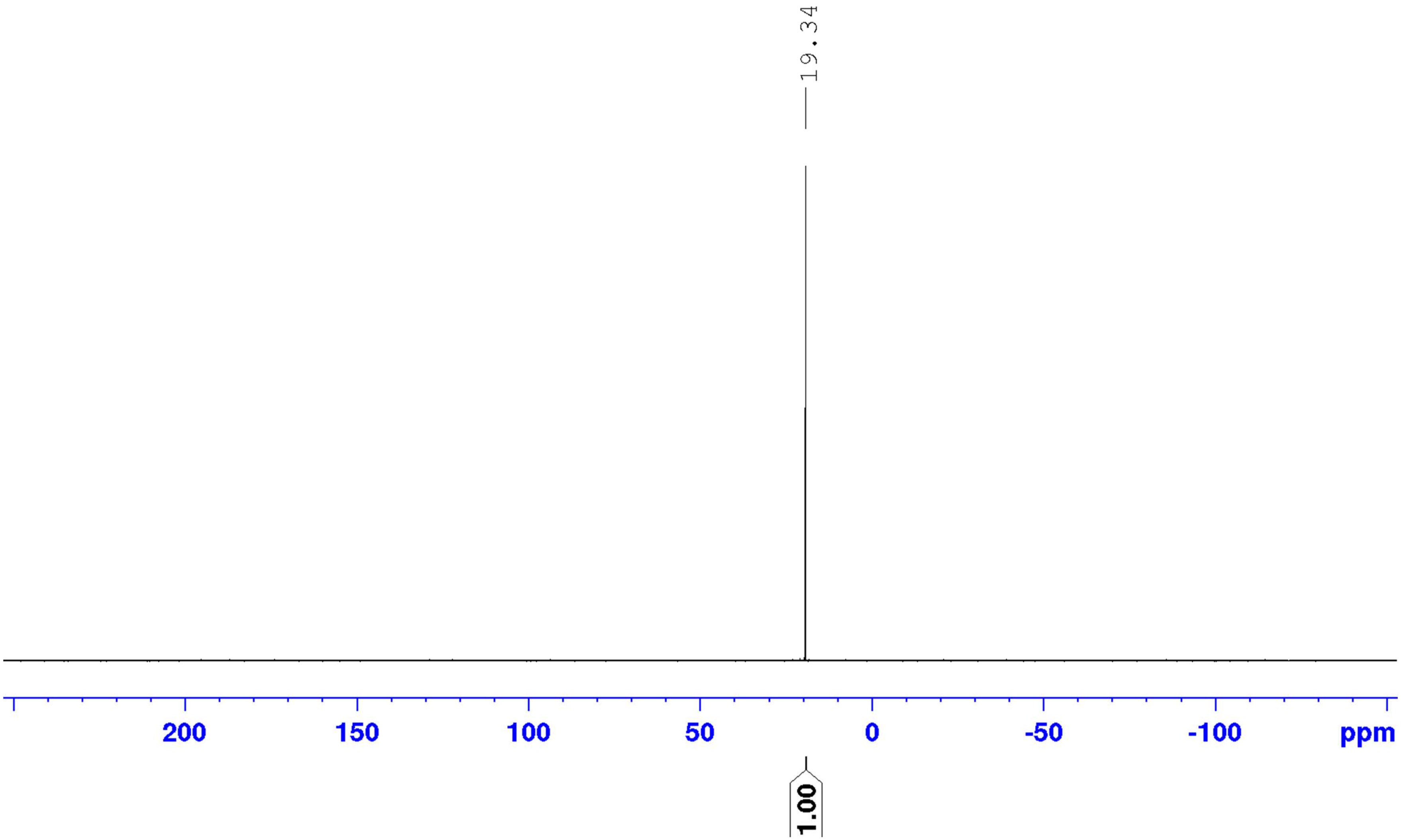

^{31}P NMR of ((*R*)-Diisopropyl 1-hydroxy-2-(*N*-methyl-*N*-tosyl-amido)-ethylphosphonate** [(*R*)-**16**] + chiral solvating agent

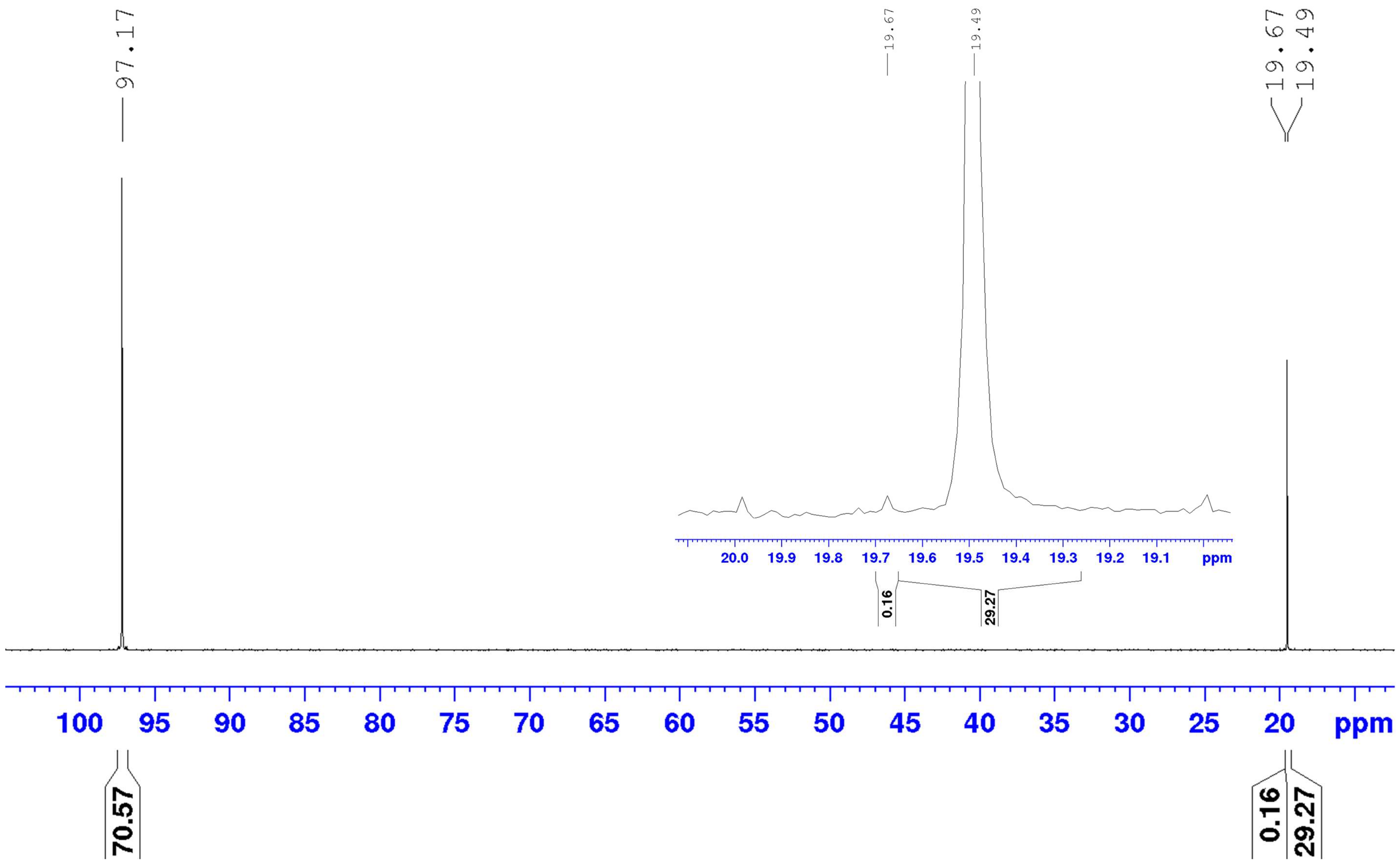
97.17
19.67
19.49
20.0
19.9
19.8
19.7
19.6
19.5
19.4
19.3
19.2
19.1
ppm
0.16
29.27
100
95
90
85
80
75
70
65
60
55
50
45
40
35
30
25
20
70.57

^{1}H NMR of (*R*)-1-Hydroxy-2-(methylammonio)ethylphosphonic acid [(*R*)-**17**, (*R*)-**M$_1$-HAEP**]

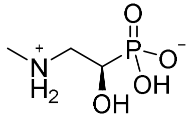

(*R*)-**M$_1$-HAEP**
(*R*)-**17**

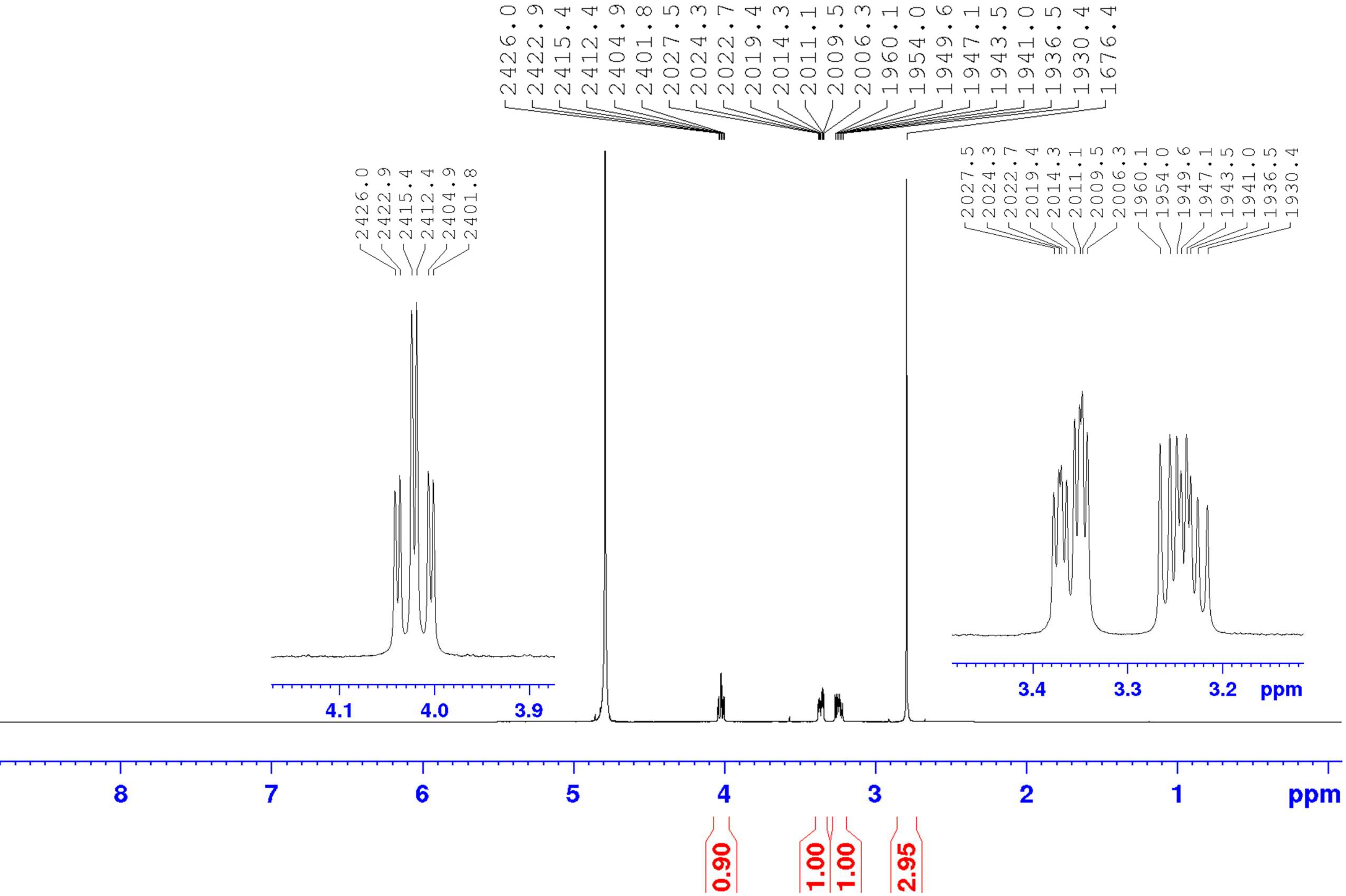

^{13}C NMR of (*R*)-1-Hydroxy-2-(methylammonio)ethylphosphonic acid [(*R*)-**17**, (*R*)-**M$_1$-HAEP**]

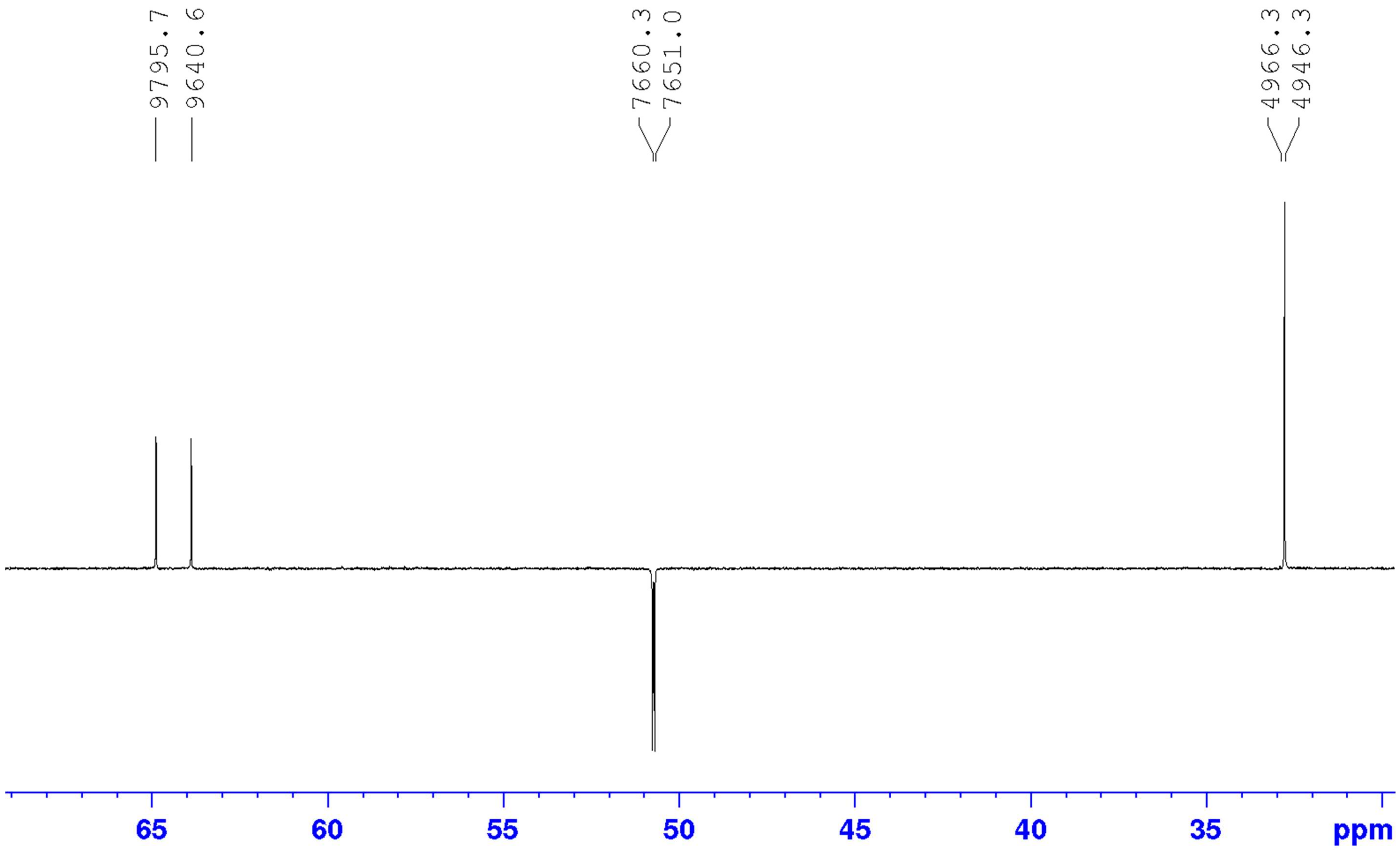

^{31}P NMR of (*R*)-1-Hydroxy-2-(methylammonio)ethylphosphonic acid [(*R*)-**17**, (*R*)-**M$_1$-HAEP**]

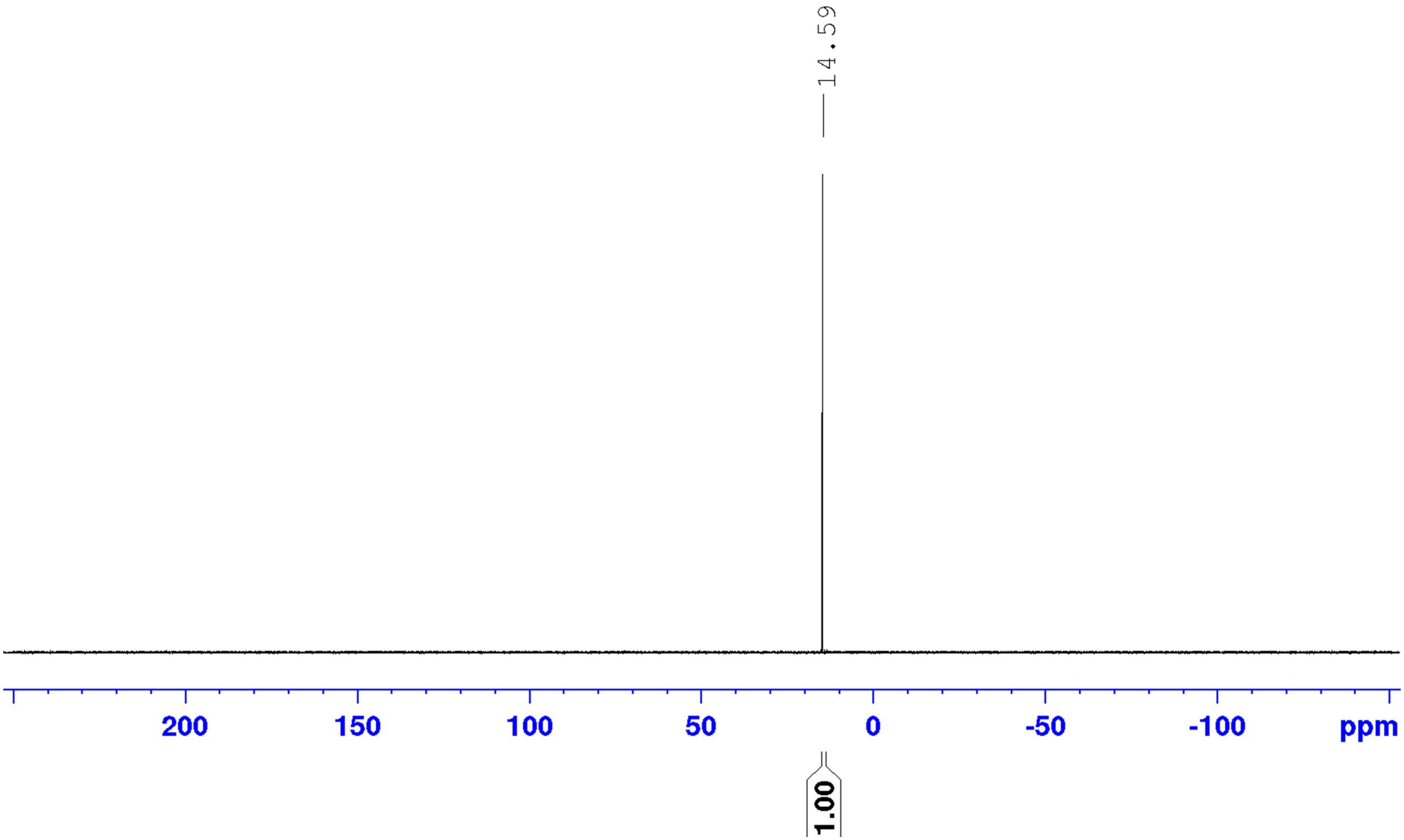

^{1}H NMR of (*R*)-1-Hydroxy-2-(dimethylammonio)-ethylphosphonic acid [(*R*)-**18**, (*R*)-**M$_2$-HAEP**]

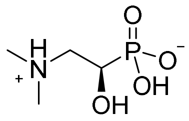

(*R*)-**M$_2$-HAEP**
(*R*)-**18**

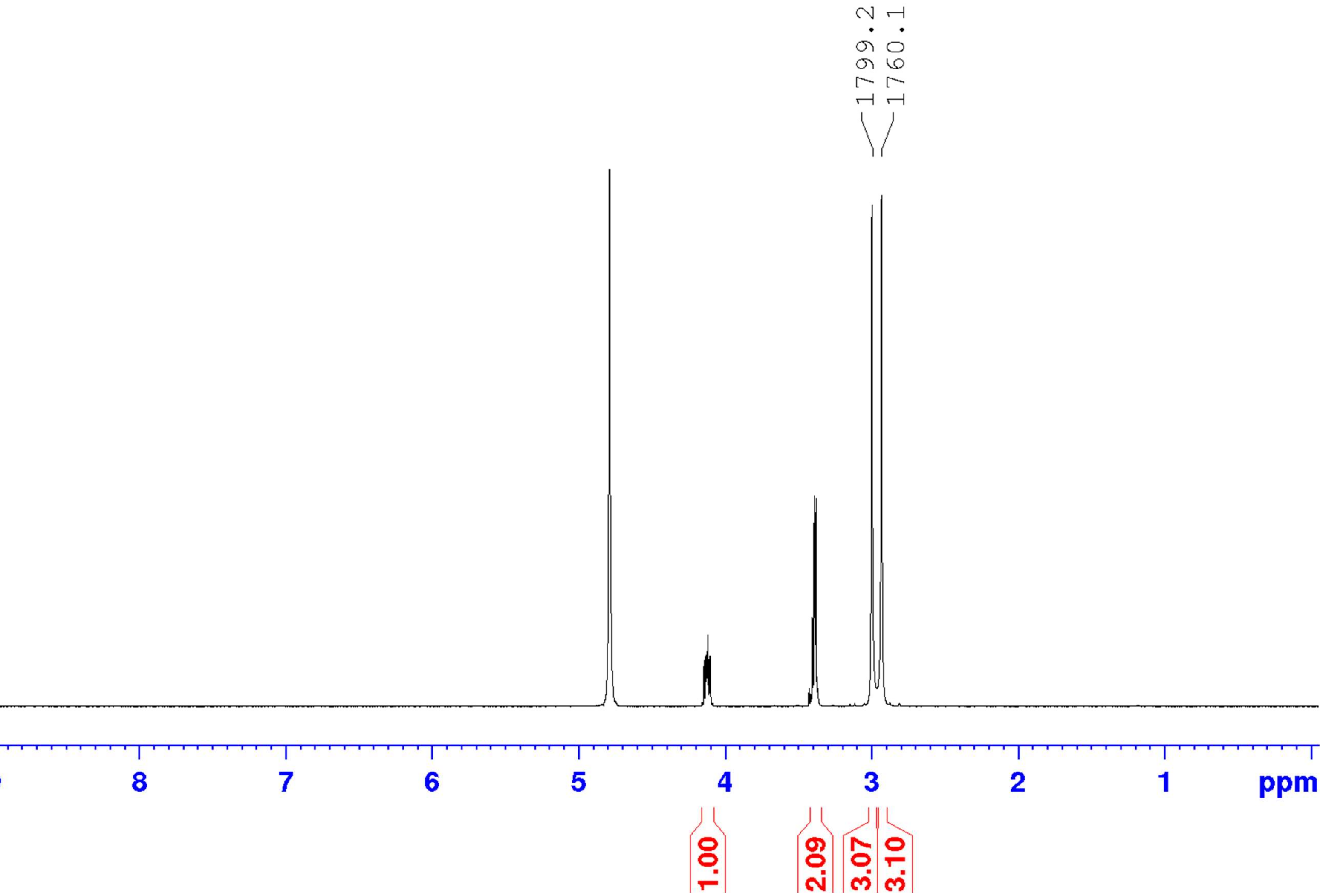

^{13}C NMR of (*R*)-1-Hydroxy-2-(dimethylammonio)-ethylphosphonic acid [(*R*)-**18**, (*R*)-**M$_2$-HAEP**]

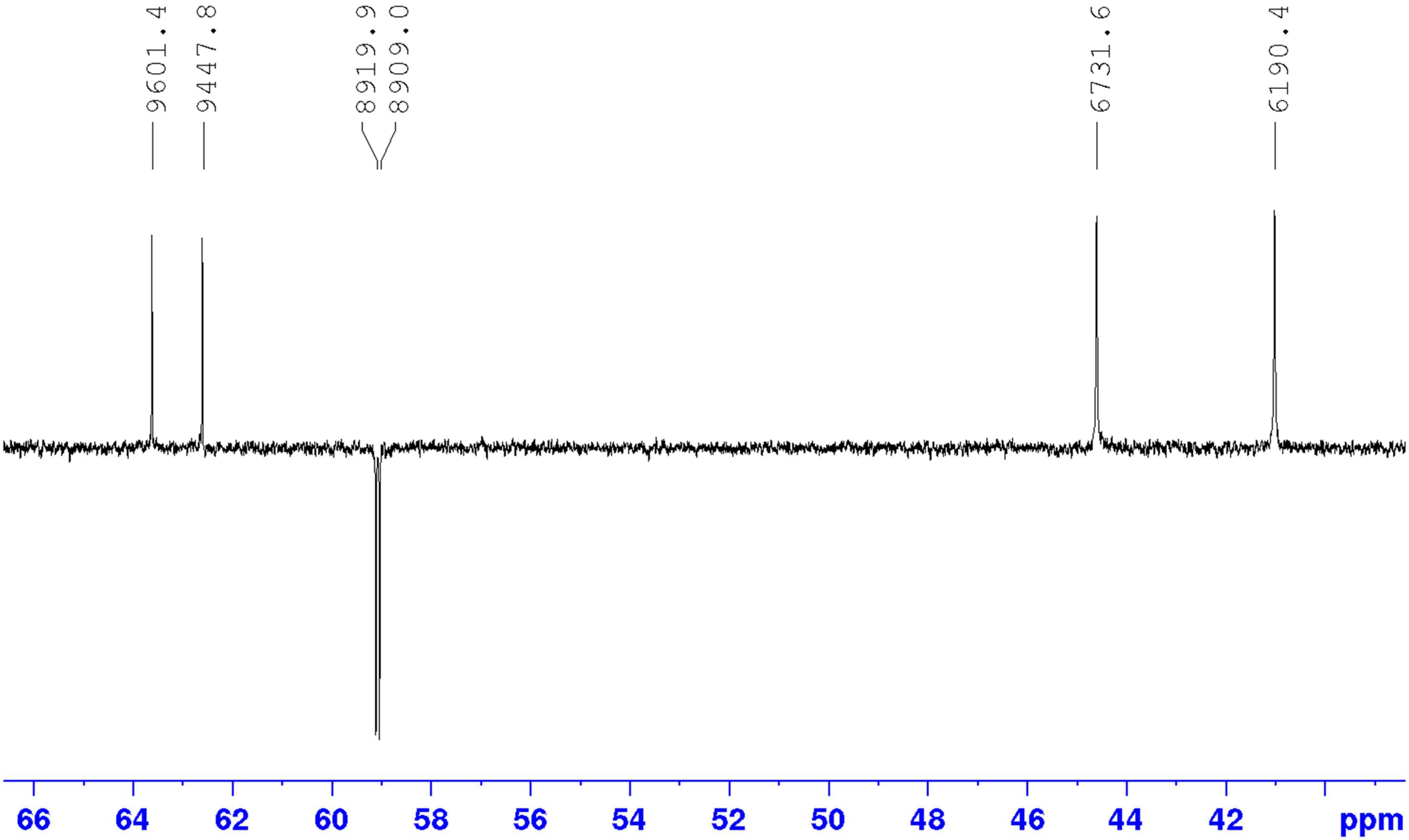

^{31}P NMR of ((*R*)-1-Hydroxy-2-(dimethylammonio)-ethylphosphonic acid [(*R*)-**18**, (*R*)-**M$_2$-HAEP**]

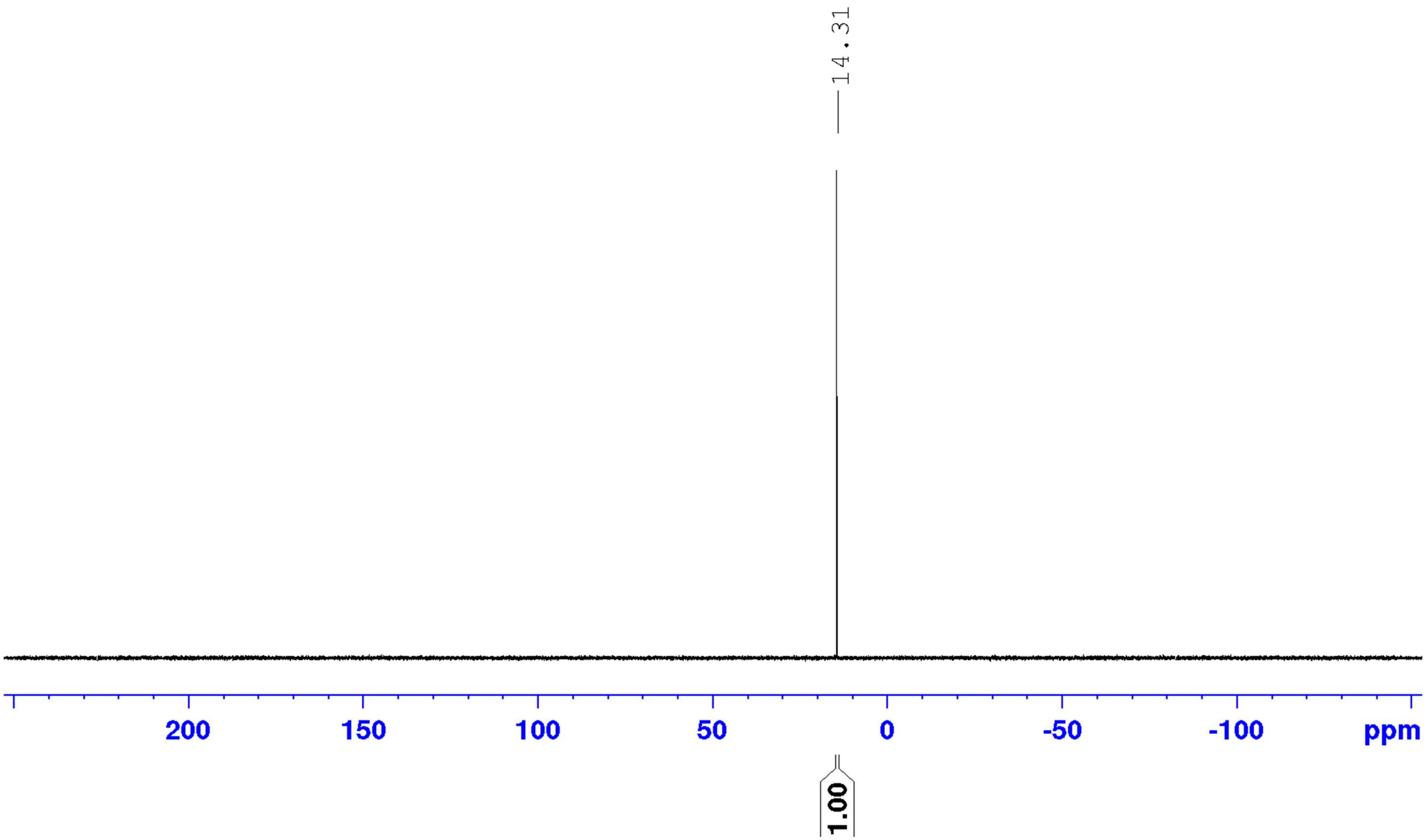

^{1}H NMR of (*R*)-1-Hydroxy-2-(trimethylammonio)-ethylphosphonic acid [(*R*)-**19**, (*R*)-**M$_2$-HAEP**]

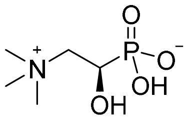

(*R*)-**M$_3$-HAEP**
(*R*)-**19**

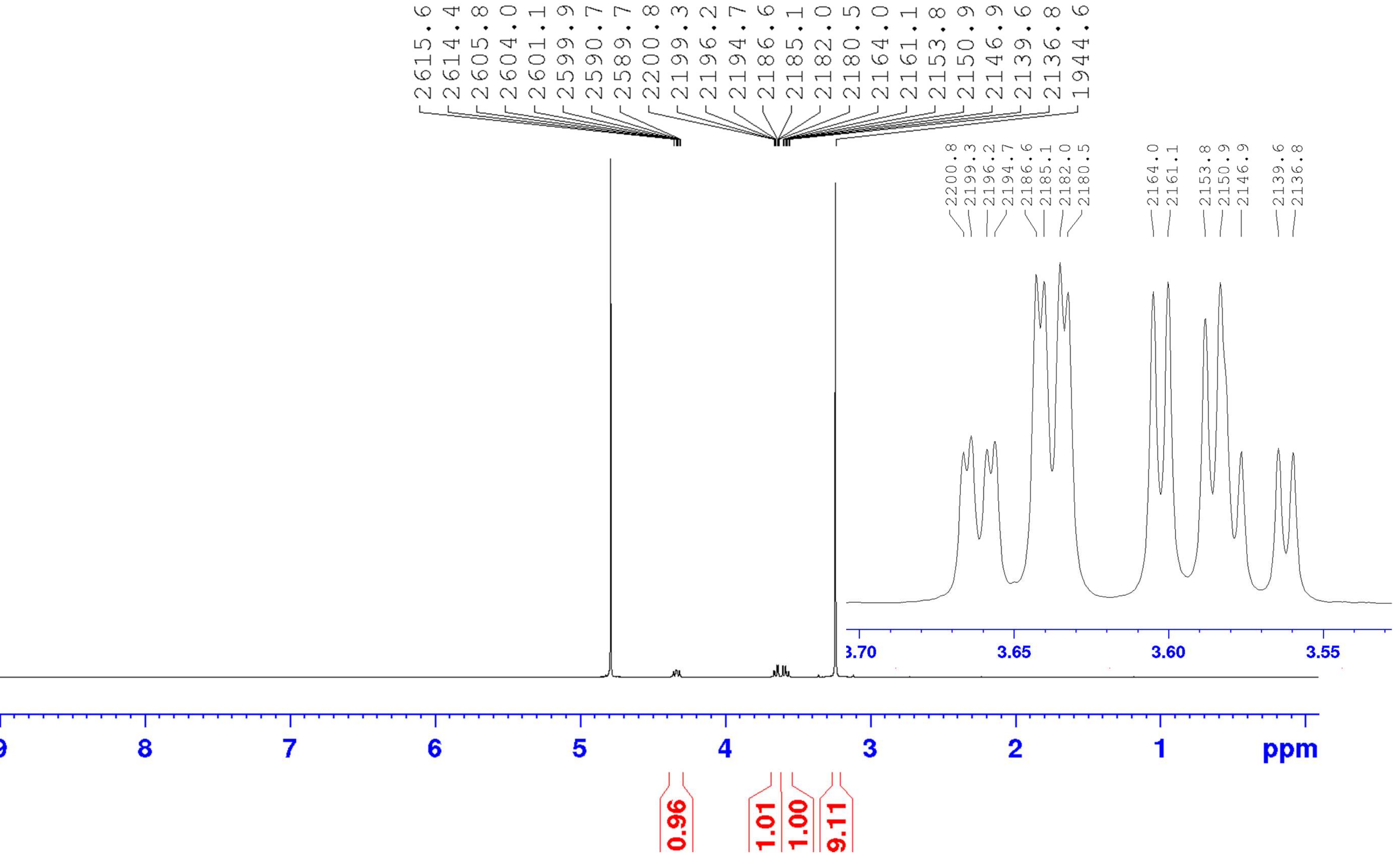

^{13}C NMR of (*R*)-1-Hydroxy-2-(trimethylammonio)-ethylphosphonic acid [(*R*)-**19**, (*R*)-**M$_2$-HAEP**]

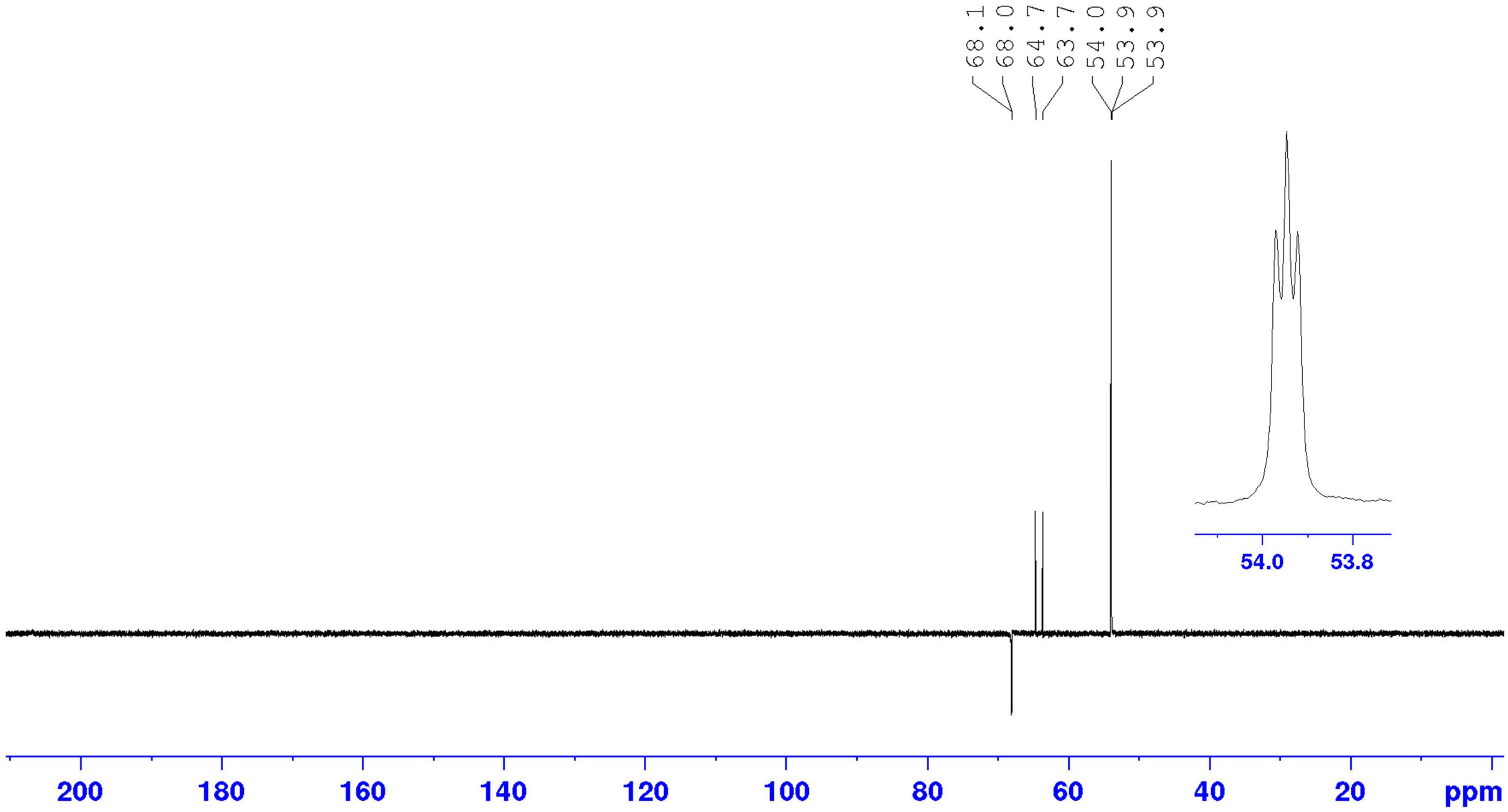

^{31}P NMR of (*R*)-1-Hydroxy-2-(trimethylammonio)-ethylphosphonic acid [(*R*)-**19**, (*R*)-**M$_2$-HAEP**]

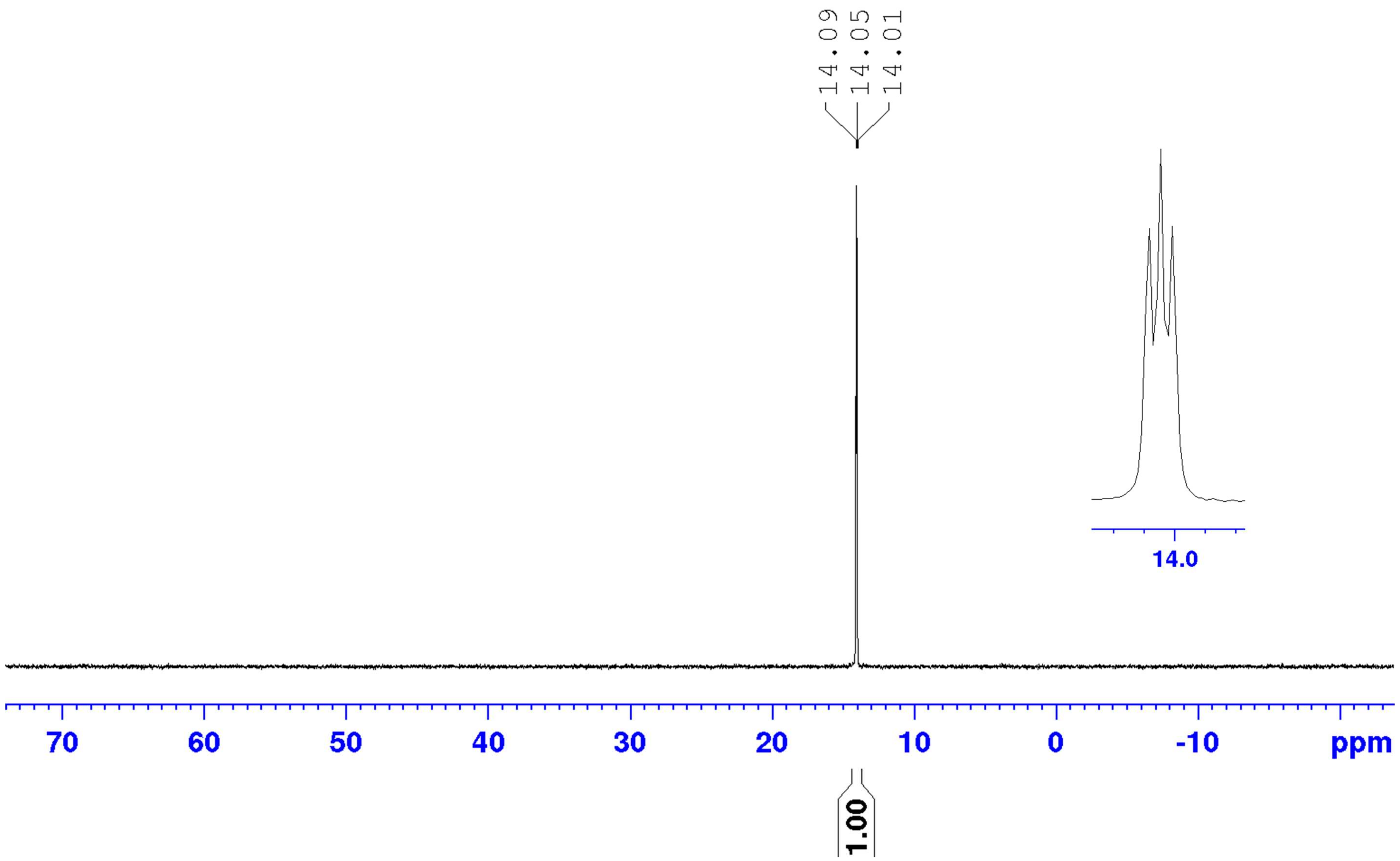

Supplemental References

1. Van Staalduinen, L.M., McSorley, F.R., Schiessl, K., Séguin, J., Wyatt, P.B., Hammerschmidt, F., Zechel, D.L., and Jia, Z. (2014). Crystal structure of PhnZ in complex with substrate reveals a di-iron oxygenase mechanism for catabolism of organophosphonates. Proc Natl Acad Sci U S A *111*, 5171–5176.

2. Arnold, B.J., Huang, I.T., and Hanage, W.P. (2022). Horizontal gene transfer and adaptive evolution in bacteria. Nat Rev Microbiol *20*, 206–218.

3. Borisova, S.A., Christman, H.D., Mourey Metcalf, M.E., Zulkepli, N.A., Zhang, J.K., Van Der Donk, W.A., and Metcalf, W.W. (2011). Genetic and biochemical characterization of a pathway for the degradation of 2-aminoethylphosphonate in *Sinorhizobium meliloti* 1021. J Biol Chem *286*, 22283–22290.

4. Zangelmi, E., Stanković, T., Malatesta, M., Acquotti, D., Pallitsch, K., and Peracchi, A. (2021). Discovery of a new, recurrent enzyme in bacterial phosphonate degradation: (*R*)-1-hydroxy-2-aminoethylphosphonate ammonia-lyase. Biochemistry *60*, 1214–1225.

5. McSorley, F.R., Wyatt, P.B., Martinez, A., DeLong, E.F., Hove-Jensen, B., and Zechel, D.L. (2012). PhnY and PhnZ comprise a new oxidative pathway for enzymatic cleavage of a carbon − phosphorus bond. J Am Chem Soc *134*, 8364−8367.

6. Gama, S.R., Suet, B., Lo, Y., Se, J., Hammerschmidt, F., Pallitsch, K., and Zechel, D.L. (2019). C − H bond cleavage is rate-limiting for oxidative C − P bond cleavage by the mixed valence diiron-dependent oxygenase PhnZ. Biochemistry *58*, 5271−5280.

7. Kurihara, S., Oda, S., Kato, K., Kim, H.G., Koyanagi, T., Kumagai, H., and Suzuki, H. (2005). A novel putrescine utilization pathway involves γ-glutamylated intermediates of *Escherichia coli* K-12. J Biol Chem *280*, 4602–4608.

8. Chiribau, C.B., Sandu, C., Fraaije, M., Schiltz, E., and Brandsch, R. (2004). A novel γ-N-methylaminobutyrate demethylating oxidase involved in catabolism of the tobacco alkaloid nicotine by *Arthrobacter nicotinovorans* pAO1. Eur J Biochem *271*, 4677–4684.

9. Zhang, K., Guo, Y., Yao, P., Lin, Y., Kumar, A., Liu, Z., Wu, G., and Zhang, L. (2016). Characterization and directed evolution of BliGO, a novel glycine oxidase from *Bacillus licheniformis*. Enzyme Microb Technol *85*, 12–18.

10. Khanna, P., and Jorns, M.S. (2001). Characterization of the FAD-containing *N*-methyltryptophan oxidase from *Escherichia coli*. Biochemistry *40*, 1441–1450.

11. Bergeron, F., Otto, A., Blache, P., Day, R., Denoroy, L., Brandsch, R., and Bataille, D. (1998). Molecular cloning and tissue distribution of rat sarcosine dehydrogenase. Eur J Biochem *257*, 556–561.

12. Brizio, C., Brandsch, R., Douka, M., Wait, R., and Barile, M. (2008). The purified recombinant precursor of rat mitochondrial dimethylglycine dehydrogenase binds FAD via an autocatalytic reaction. Int J Biol Macromol *42*, 455–462.

13. Tanigawa, M., Shinohara, T., Saito, M., Nishimura, K., Hasegawa, Y., Wakabayashi, S., Ishizuka, M., and Nagata, Y. (2010). D-Amino acid dehydrogenase from *Helicobacter pylori* NCTC 11637. Amino Acids *38*, 247–255.

14. Wagner, M.A., and Jorns, M.S. (2000). Monomeric sarcosine oxidase: 2. Kinetic studies with sarcosine, alternate substrates, and a substrate analogue. Biochemistry *39*, 8825–8829.
